# Cytotoxicity of Ruthenium(II) Arene Complexes Containing Functionalized Ferrocenyl β-Diketonate Ligands

**DOI:** 10.1021/acs.organomet.2c00553

**Published:** 2023-07-05

**Authors:** Matthew Allison, Pablo Caramés-Méndez, Benjamin J. Hofmann, Christopher M. Pask, Roger M. Phillips, Rianne M. Lord, Patrick C. McGowan

**Affiliations:** † School of Chemistry, University of Leeds, Woodhouse Lane, Leeds LS2 9JT, U.K.; ‡ Department of Pharmacy, University of Huddersfield, Huddersfield HD1 3DH, U.K.; § School of Chemistry, University of East Anglia, Norwich Research Park, Norwich NR4 7TJ, U.K.; ∥ School of Chemistry and Biosciences, University of Bradford, Bradford BD7 1DP, U.K.

## Abstract

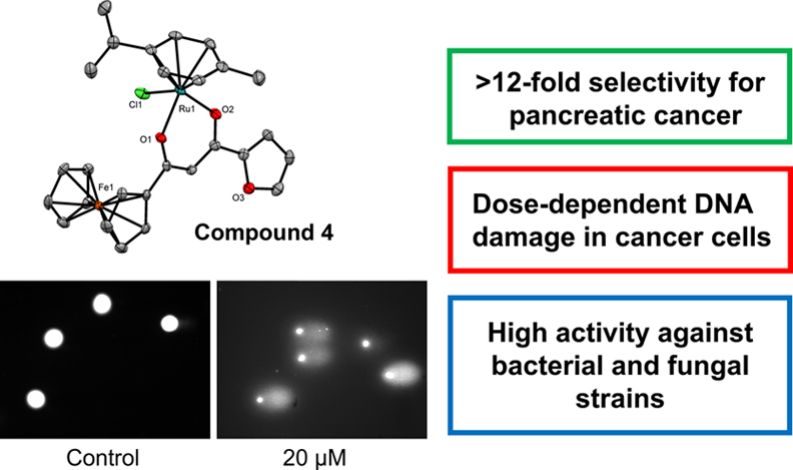

The synthesis and characterization of 24 ruthenium(II) arene complexes of the type [(*p*-cym)RuCl(Fc-acac)] (where *p*-cym = p-cymene and Fc-acac = functionalized ferrocenyl β-diketonate ligands) are reported, including single-crystal X-ray diffraction for 21 new complexes. Chemosensitivity studies have been conducted against human pancreatic carcinoma (MIA PaCa-2), human colorectal adenocarcinoma *p53*-wildtype (HCT116 *p53*^+/+^) and normal human retinal epithelial cell lines (APRE-19). The most active complex, which contains a 2-furan-substituted ligand (**4**), is 5x more cytotoxic than the analogs 3-furan complex (**5**) against MIA PaCa-2. Several complexes were screened under hypoxic conditions and at shorter-time incubations, and their ability to damage DNA was determined by the comet assay. Compounds were also screened for their potential to inhibit the growth of both bacterial and fungal strains.

## Introduction

Ruthenium is relatively well tolerated by the body,^1^ and the rate of ligand exchange from the metal center is slow in comparison to many other transition-metal complexes.^2^ This can lead to high kinetic stability and minimize the possibility of side reactions. Tuning the ligand environment and metal oxidation states can help control the thermodynamic and kinetic properties of the complexes in order to control their biological activity.^3^ Therefore, ruthenium research has produced a plethora of potential therapeutics with a wide range of structures, geometries, and oxidation states and easily tunable ligand environments.

**Figure 1:**
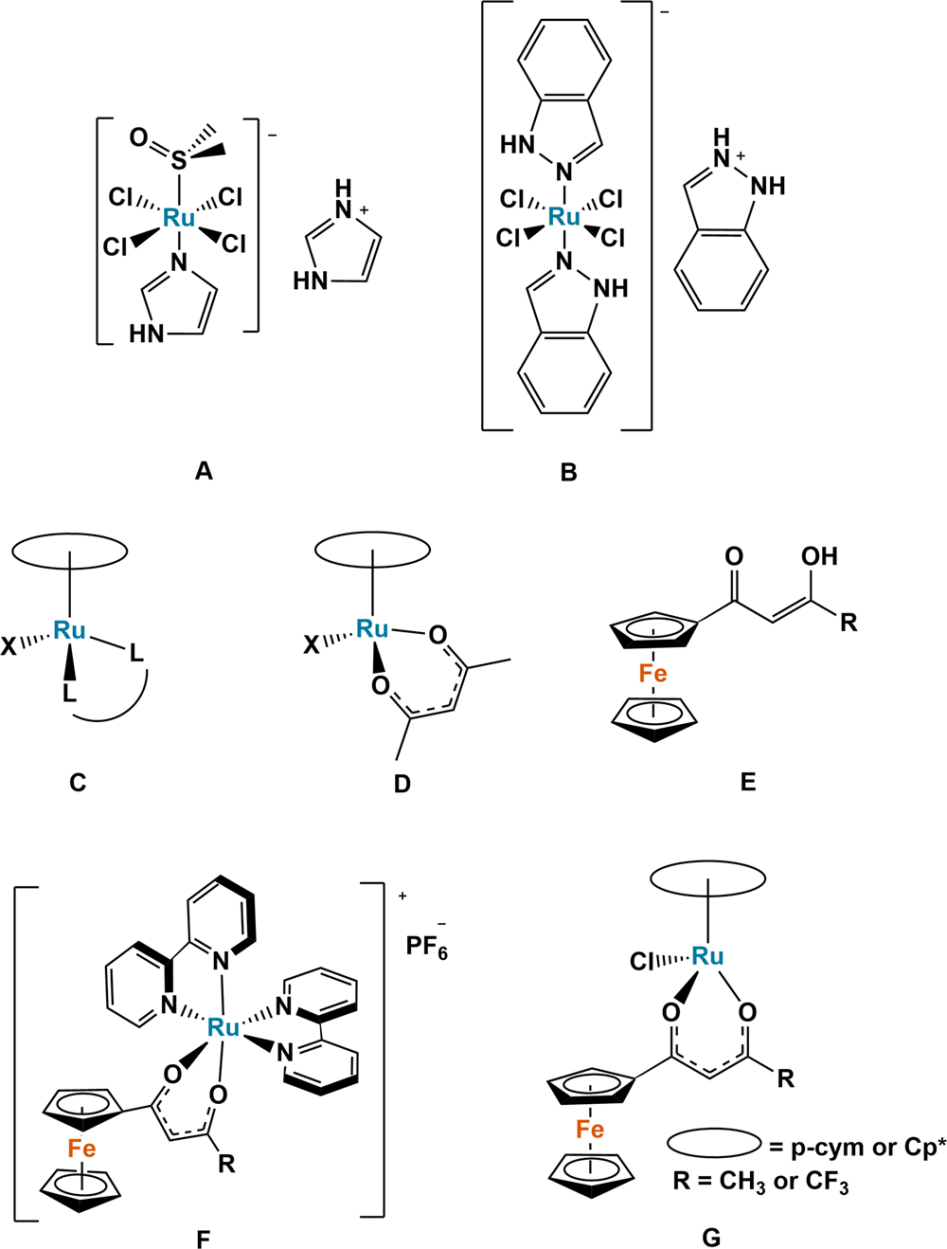
Structures of published and cytotoxic ruthenium complexes NAMI-A (**A**), KP1019 (**B**), piano-stool arenes (**C**, **D**) , ferrocenyl β-diketonates (**E**) and heterobimetallic ruthenium–ferrocenyl β-diketonates (**F**, **G**).

Over the last two decades, there has been a surge in anticancer research for ruthenium coordination compounds^4,5^ and this stemmed from the promising cytotoxicity of Ru(III) compounds NAMI-A (ImH)[*trans*-RuCl_4_(DMSO)(Im)] (Figure 1A; Im = imidazolium) and KP1019 (HInd)[*trans*-RuCl_4_(Ind)_2_], Ind = indazolium) (Figure 1B).^6^ Such ruthenium complexes have different modes of action than cisplatin (CDDP), including the ability to target cancer cells that are resistant to platinum treatment.^7^

To date, Ru(II) organometallic “piano stool” complexes of the type [(arene)RuX(L)]^0/+^ (X = halide; L = bidentate ligand) (Figure 1C) make up a large portion of the ruthenium-based anticancer research.^4^ The effects of the ligand environment on cytotoxic potential have been explored by many research groups. For example, Sadler et al. reported the high in vitro cytotoxicity of [(η_6_-biphenyl)RuCl(en)][PF_6_] (RM175),^8,9^ which has multiple binding modes to nucleic acids,^10^l-cysteine, and methionine.^11^ Complexes were inactive with N,O-chelating ligands, whereas complexes containing an O,O-chelating acetylacetone (e.g., acac Figure 1D) ligand had reasonable cytotoxicity toward ovarian carcinoma (A2780) and weak binding to nucleobases.^12^ We have also reported the cytotoxic potential of Ru(II) arene complexes with different ligand-binding modes whereby β-ketoiminate N,O-bound ligands are considerably more effective than N,N-picolinamide and O,O-acetylacetone-type ligands following the trend N,O > N,N > O,O.^13,14^

Ferrocene has become an increasingly prevalent addition to many already well-established anticancer compounds, as its addition is well documented to increase compounds cytotoxicity.^15,16^ Ferrocenyl-based compounds can act as “redox antennas”, aiding in the formation of reactive oxygen species (ROS) and leading to DNA damage.^17^ We have recently shown that the incorporation of ferrocene into β-diketonate ligands to synthesize functionalized ferrocenyl β-diketonate ligands (Fc-acac; Figure 1E) can significantly increase the compounds’ cytotoxicity by up to 18-fold against human breast adenocarcinomas (MCF-7 and MDA-MB-231).^18^

Heterobimetallic species have the potential benefit of both metals working together, and by combining ruthenium and ferrocene into a singular complex, it is possible to have synergistic effects against cancer cells.^17,19,20^ Alongside other research groups, in 2021, we reported the synthesis of heterobimetallic ruthenium–ferrocenyl complexes, [(bpy)_2_(Fc-acac)Ru][PF_6_] (bpy = 2,2′-bipyridine; Figure 1F)^21^ and highlighted their excellent nanomolar cytotoxicity against both human pancreatic carcinoma (MIA PaCa-2) and human colorectal carcinoma *p53*-wildtype (HCT116 *p53*^+/+^) cell lines with dose-dependent double-strand DNA damage, which is correlated to their cytotoxicity.

In 2022, Manikandan et al. reported Ru(II) piano-stool complexes of the type [(arene)RuCl(Fc-acac)], where arene = *p*-cymene (*p*-cym) or 1,2,3,4,5-pentamethylcyclopentadiene (Cp*) and Fc-acac = a functionalized ferrocenyl β-diketonate ligand with either a methyl or trifluoromethyl group (Figure 1G).^22^ These complexes had moderate to high activity against a range of cell lines with the activity of the trifluoromethyl complex being up to 19-fold higher than that of the methyl complex against A2780 ovarian carcinoma. However, the activity did not improve when the *p*-cym ligand was exchanged for Cp*.

**Scheme 1:**
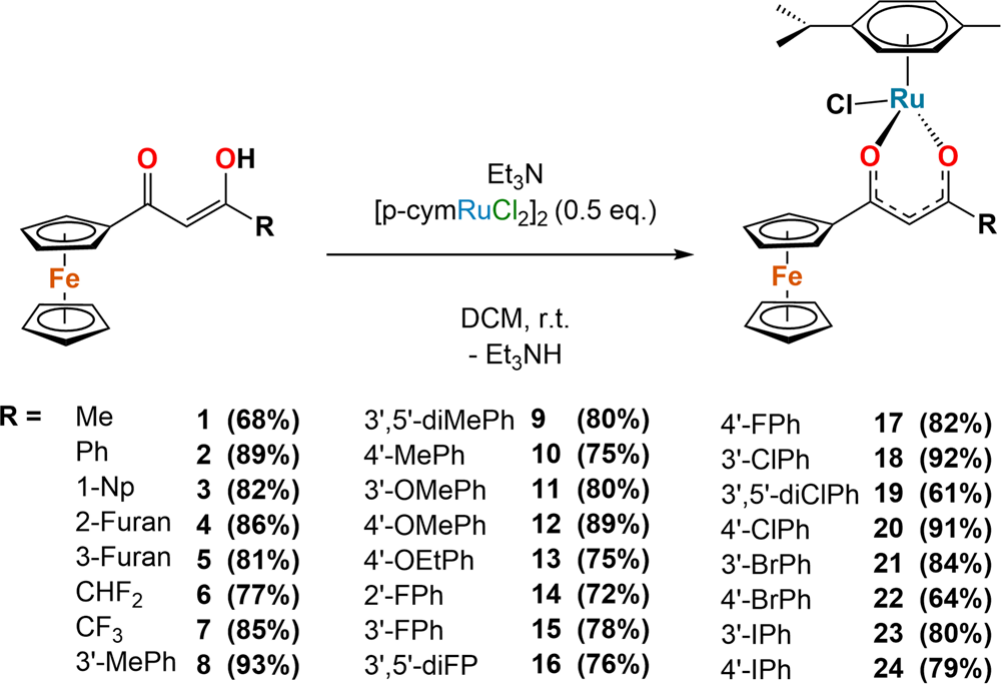
General Synthetic Pathway for the Synthesis of *p*-Cymene Ru(II) Ferrocenyl β-Diketonato Complexes [(*p*-cym)RuCl(Fc-acac)] **1**–**24**

We have extended the library of Ru(II) piano-stool complexes to include 22 new complexes and the two recently reported complexes by Manikandan et al. (Scheme 1, **1** and **7**).^22^ This includes the analysis of 21 new molecular structures via single-crystal X-ray diffraction (sc-XRD). All compounds have been screened for their cytotoxic potential using the MTT assay against MIA PaCa-2 and HCT116 *p53*^+/+^ cancer cell lines and a normal human retinal epithelium cell line, ARPE-19. Additional studies were conducted on the ligand and complex stability by NMR spectroscopy, intracellular metal uptake by ICP-MS, cyclic voltammetry to assess accessible redox potentials, cytotoxicity under severe hypoxic (0.1% O_2_) conditions at shorter exposure times, and double-strand breakages (DSB) of DNA using the comet assay. The compounds have also been screened for their potential to inhibit the growth of several bacterial and fungal strains.

## Results and Discussion

### Synthesis and Characterization

The ferrocenyl β-diketonate ligands (Fc-acac) were synthesized using a Claisen condensation reaction and our previously reported literature methods.^18,21^ Ligands **L1-L4**, **L7**, **L9-L10**, **L12**, **L15-L20**, **L22** and **L24** have been previously reported,^18^ whilst the remaining ligands were obtained as analytically pure compounds from column chromatography in yields of 23–97% (Scheme S1). The p-cymene Ru(II) ferrocenyl β-diketonate complexes, [(p-cym)RuCl(Fc-acac)] **1–24**, were prepared by adaptations of previously published methods,^14^ whilst **1** and **7** have been recently reported.^22^ A functionalized ferrocenyl β-diketonate ligand (2 eq.) was stirred at room temperature overnight with triethylamine (2 eq.) and [(p-cym)RuCl_2_]_2_ (1 eq.) in dichloromethane (Scheme 1). The complexes were purified by column chromatography and obtained as orange microcrystalline solids in yields of 67–91%. All ligands and complexes have been fully characterized by ^1^H NMR and ^13^C{^1^H} NMR spectroscopy, mass spectrometry and elemental analysis. The ^1^H NMR spectra show all [(*p*-cym)RuCl(Fc-acac)] complexes have a shift in the ferrocenyl protons to lower frequencies. Due to the introduction of a chiral center and a loss of symmetry caused by the restricted rotation of the top Cp ring (e.g., Figure S8), the resonances for free ligand appear as two 2H broad triplets (*ca.* 4.75 and 4.50 ppm) which move to two 1H doublet of triplets (*ca.* 4.80 and 4.58 ppm) and two 1H triplet of doublets (*ca.* 4.35–4.30 ppm).

Red/orange single crystals of ligands **L5**, **L8**, **L11**, **L13**, **L14**, and **L23** (Figure S1) were obtained from slow evaporation of acetonitrile. Molecular structures were determined by sc-XRD analysis (Tables S1 and S2) with structural solutions performed in a tetragonal (**L5**), monoclinic (**L8** and **L13** and **L14**), orthorhombic (**L11**), or tetragonal (**L23**) space groups. All molecules display a planar structure with angles of 119–122° around the enol/keto center (Tables S3 and S4) and the ferrocenyl group adopts an eclipsed geometry, as discussed in our previous work.^18^ In all cases, intramolecular hydrogen bonding is observed between enol–keto with O–H···O distances ranging between 2.46 and 2.50 Å (D–A), thus restraining the geometry to a planar orientation.

**Figure 2:**
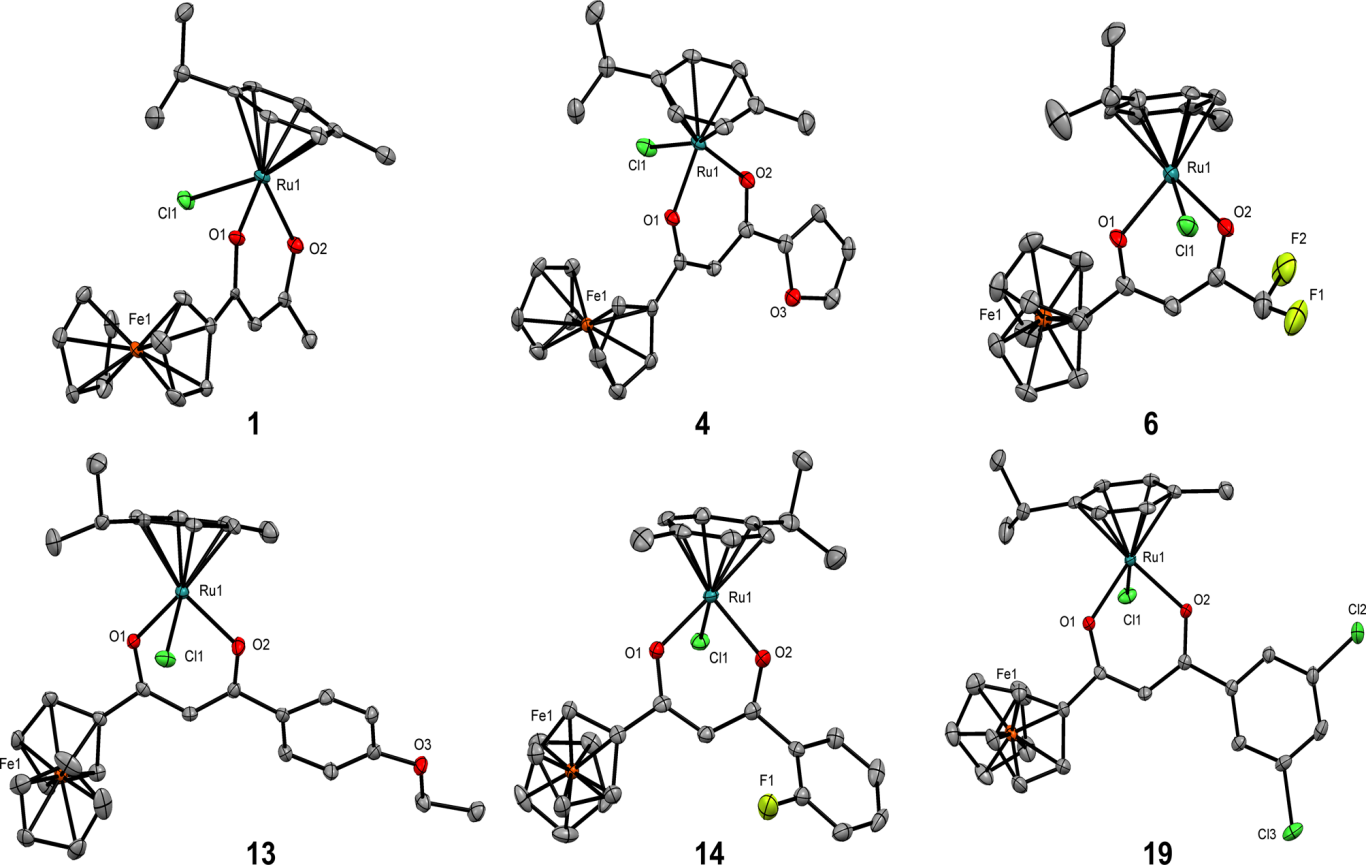
Examples of the molecular structures for complexes **1**, **4**, **6**, **13**, **14**, and **19**. Hydrogen atoms and disordered atoms are omitted for clarity. Displacement ellipsoids are placed at the 50% probability level.

Red/orange single crystals of complexes **1**, **2**, **4**–**6**, and **9**–**24** (examples in Figure 2 and Figures S2–S7) were obtained from either the slow evaporation of acetonitrile or the vapor diffusion of dichloromethane/pentane at 4 °C. Sc-XRD analysis (Tables S5–S10) was obtained, and solutions were performed in monoclinic cells except for complexes **6** (orthorhombic) and **16** (triclinic). All ruthenium arene complexes adopt the expected *pseudo*-octahedral “piano stool” geometries with the angles around the ruthenium metal center in the range of 84–89° (Tables S11 and S12). Intramolecular interactions (D···A = 3.4–4.0 Å) are seen between the *p*-cymene isopropyl group and the chloride bound to the ruthenium center (C10/11–H···Cl1) in all cases (except **6**) with additional intermolecular interactions (D···A = 3.2–4.0 Å) observed in all complexes. These interactions could explain the shift to a lower frequency for the *p*-cymene hydrogens, which was also observed in our previous arene-Ru(II) work.^14^

### Chemosensitivity Studies

**Figure 3:**
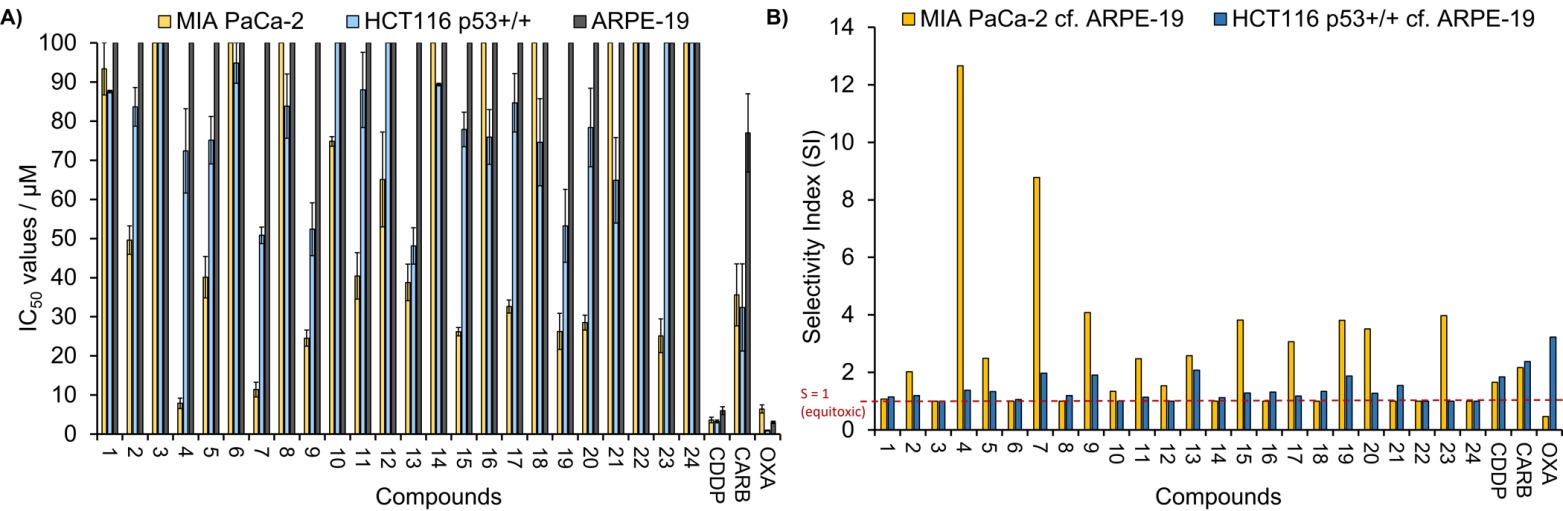
(A) IC_50_ values (μM) ± SD of complexes **1**–**24** and cisplatin (CDDP), carboplatin (CARB), and oxaliplatin (OXA) when screened against MIA PaCa-2, HCT116 *p53*^+/+^, and ARPE-19 cell lines. (B) Selectivity index (SI) for complexes **1**–**24**, CDDP, CARB, and OXA when the IC_50_ values against the normal cell line ARPE-19 are compared to that of the cancer cell lines. SI > 1 shows selectivity for the cancer cell lines, SI = 1 shows equitoxicity (red dotted line), and SI < 1 shows selectivity for the normal cell line.

To deduce any structural–activity relationships (SARs), chemosensitivity studies were performed for complexes **1**–**24**, cisplatin (CDDP), carboplatin (CARB), and oxaliplatin (OXA) using a 96 h MTT assay (Figure 3A and Table S13). All compounds were screened against human pancreatic carcinoma (MIA PaCa-2) and human colorectal adenocarcinoma *p53*-wildtype (HCT116 *p53*^+/+^). The results show that complexes display varying cytotoxicity toward both cancer cell lines tested with a general increase in activity observed against MIA PaCa-2. The 2-furan Fc-acac complex **4** exhibited the highest cytotoxicity (IC_50_ = 8 ± 2 μM, cf. CDDP = 3.6 ± 0.7 μM) against MIA PaCa-2 followed by the trifluoromethyl Fc-acac complex **7** (IC_50_ = 11 ± 1 μM). Although **4** exhibits the highest IC_50_ value against MIA PaCa-2, it has a significantly lower potency than the Ru(II) coordination analogues we have previously reported (e.g., Figure 1F; IC_50_ value = 0.11 ± 0.01 μM).^21^

Complex **1**, which contains an unfunctionalized Fc-acac ligand, has low activity against all cell lines (IC_50_ = 92–93 μM), and highlights that functionalizing the methyl with electron-withdrawing groups improves the cytotoxicity. The addition of a phenyl substituent (**2**) increases the activity by ca. 2-fold against MIA PaCa-2 (IC_50_ = 50 ± 6 μM); however, by increasing the hydrophobicity further from phenyl (**2**) to naphthyl (**3**), the IC_50_ values decrease and complex **3** is inactive against all cell lines (IC_50_ > 100 μM), confirming that the IC_50_ values do not correlate with the hydrophobicity. The hydrophobicity of each complex were obtained from an octanol–water shake-flask method, and the data is shown in Table S14. It should be noted that, when comparing the recent work of Manikandan et al., complexes **1** and **7** follow the same trend^22^ whereby the activity of **7** > **1**. However, their activities are significantly lower than those reported against HeLa (cervical), A2780 (ovarian) and A2780cisR (cisplatin resistant ovarian). This highlights potential selectivity toward these cell lines and highlights the need for further screening of our library against a wider range of cancerous cells.

Poor cancer cell selectivity is one of the major contributing factors associated with the harmful side effects of chemotherapy drugs and therefore restricts the dosage that can be administered. Not only does this dose-limiting toxicity cause adverse effects, but it also impedes the effectiveness of the treatment. Comparing the response of cancer cell lines to the normal cells can give a good indication of preferential selectivity. Chemosensitivity studies against normal epithelial cell line ARPE-19 were performed for complexes **1**–**24** (Figure 3A and Table S13) whereby all compounds were non-toxic toward this cell line at the maximum tested concentrations (>100 μM), demonstrating excellent chemoselectivity toward the cancer cell lines MIA PaCa-2 and HCT116 *p53*^+/+^. This contrasts with CDDP and OXA, which remain cytotoxic toward normal cell lines; IC_50_ = 6 ± 1 μM and 3.0 ± 0.3 μM, respectively. The IC_50_ values have been expressed as a selectivity index (SI), which are calculated by dividing the IC_50_ value against the normal cell line by the IC_50_ value against the cancer cell line (Table S13). SI > 1 indicates selectivity for the cancer cell line over the normal cell line ARPE-19. Unlike OXA, which is selective for HCT116 *p53*^+/+^ (SI = 3.2), complexes **1**–**24** have low selectivity toward this cell line with SI values ranging from 1.0 to 2.1. When analyzing the SI values for all complexes against MIA PaCa-2, there is greater selectivity than both CDDP (SI = 1.7) and OXA (SI = 1.0) with SI values of up to 12.7 for complex **4** (Figure 3B). This compound is also >7.5× and >27× more selective than CDDP and OXA, respectively.

### Modes of Action

#### Complex Stability

To understand the lack of activity in our complexes, the stability has been assessed by UV–vis spectroscopy in 10% H_2_O over 96 h (Table S16 and Figures S15–S18). Several changes were observed for all complexes, which includes the darkening of the samples from red to brown and bathochromic or hypo/hyperchromic shifts. The UV–vis spectra are assigned tentatively from TD-DFT calculations on similar structures.^23−25^ In most complexes (except **6** and **7**), there is a hyperchromic shift of a newly formed MLCT band in the region of 330–370 nm and ligand-based absorbance at 220–230 nm. These changes in the spectral properties of the complexes strongly suggest that there are changes to the Ru–Cl bond but they are not conclusive. To assign the structural changes, ^1^H NMR spectroscopy was measured first in DMSO-*d*_6_ and then in 90% DMSO-*d*_6_/10% D_2_O (Figures S19–S32 for ligands and Figures S33–S48 for complexes). Attempts were made to increase the water content (>10%) of the samples; however, a significant amount of complex precipitation was observed, affecting the overall concentrations and impeding a full analysis.

Complexes **1**, **2**, **4** and **7** and the corresponding ferrocenyl β-diketonate ligands **L1**, **L2**, **L4** and **L7** were analyzed after initial (ca. 5 min), 20 and 40 min and then between 1 and 96 h at 293 K (ca. 5 mg/mL). On analysis of the ferrocenyl ligands in both 100% DMSO-*d*_6_ and 90% DMSO-*d*_6_/10% D_2_O, complete decomposition is observed by ca. 12 h to give free Cp (6.5 ppm) and a paramagnetic species. The speed at which decomposition happens is faster in the presence of 10% D_2_O. When comparing complexes **1**, **2**, **4**, and **7** to the corresponding ligands **L1**, **L2**, **L4**, and **L7** after 96 h, the stability of the complexes is enhanced, where although some free Cp is generated, it is slower than the ligand alone.

In 100% DMSO-*d*_6_, complexes **2** and **4** are the most stable and do not change over 96 h, whereas complexes **1** and **7** show some decomposition to free *p*-cymene, which is observed in the new signals at 7.10, 2.95, 2.22, and 1.16 ppm (Figure S49). When analyzed in the presence of 10% D_2_O, all complexes decompose by 96 h to free *p*-cymene, free Cp, and a paramagnetic species. It should be also noted that, unlike the work reported by Manikandan et al.,^22^ we do not observe an aqua species, which is likely due to rapid exchange. To further assess this, we have conducted NMR studies of complex **1** in the presence of 100 mM NaCl (Figures S50–S52), and decomposition to free *p*-cymene and Cp still occurs but to a lower degree.

#### Cellular Uptake

**Figure 4:**
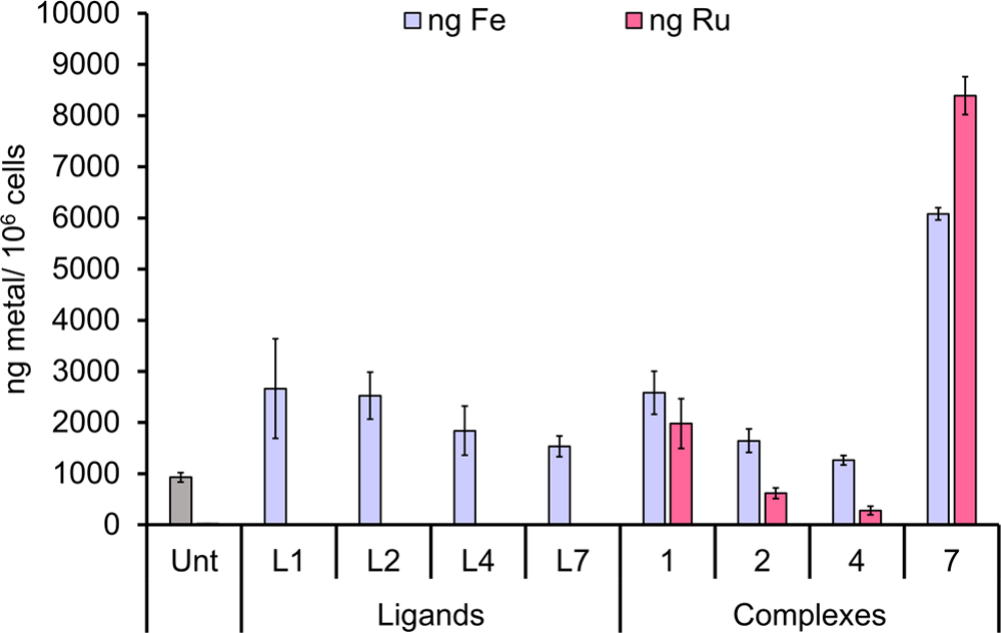
Whole cell uptake of ligands **L1**, **L2**, **L4**, and **L7** (Fe only) and complexes **1**, **2**, **4**, and **7** (Fe and Ru). The data is shown in ng of metal per million cells after MIA PaCa-2 were treated with 10 μM of the compounds for 48 h.

It has been reported that cytotoxicity can be related to the uptake of compounds into the cell. The uptake of complexes **1**, **2**, **4** and **7** and their corresponding ferrocenyl β-diketonate ligands **L1**, **L2**, **L4** and **L7** have been assessed after MIA PaCa-2 cells were treated for 48 h with 10 μM of each compound. To understand the cytotoxicity relationships, MTT assays of all compounds were conducted after 48 h of incubation (Table S15). The cytotoxicity of the ligands follows the order **L7** > **L4** > **L1** ≈ **L2**, while the uptake of Fe follows the opposite trend whereby **L7** has the lowest uptake of 1536 ± 202 ng Fe/10^6^ cells (Figure 4; ca. 1.7× increase compared to the control = 929 ± 93 ng Fe/10^6^ cells). The least active ligands **L1** and **L2** have a higher uptake of 2661 ± 974 ng Fe/10^6^ cells and 2524 ± 458 ng Fe/10^6^ cells, respectively.

When considering the cytotoxicity of the complexes after 48 h, complexes **4** (IC_50_ = 23 ± 2 μM) and **7** (IC_50_ = 30 ± 1 μM) have similar activity; however, their uptake is very different, suggesting different modes of action. Complex **4** has overall the lowest uptake of 1264 ± 229 ng Fe/10^6^ cells and 281 ± 82 ng Ru/10^6^ cells, which is not significant when compared to the control alone. It should be noted that the uptake of complex **7** (6077 ± 119 ng Fe/10^6^ cells and 8388 ± 369 ng Ru/10^6^ cells) is significantly enhanced when compared to the corresponding ligand, where the intracellular Fe content is increased by approximately 4-fold. While there is no correlation between the cytotoxicity and uptake of these compounds, the binding of the ferrocenyl β-diketonate ligands to the Ru(II) center does increase the cellular uptake, and this is possibly linked to the complexes increased stability, which we also observed in the NMR studies.

#### Redox Chemistry

**Figure 5:**
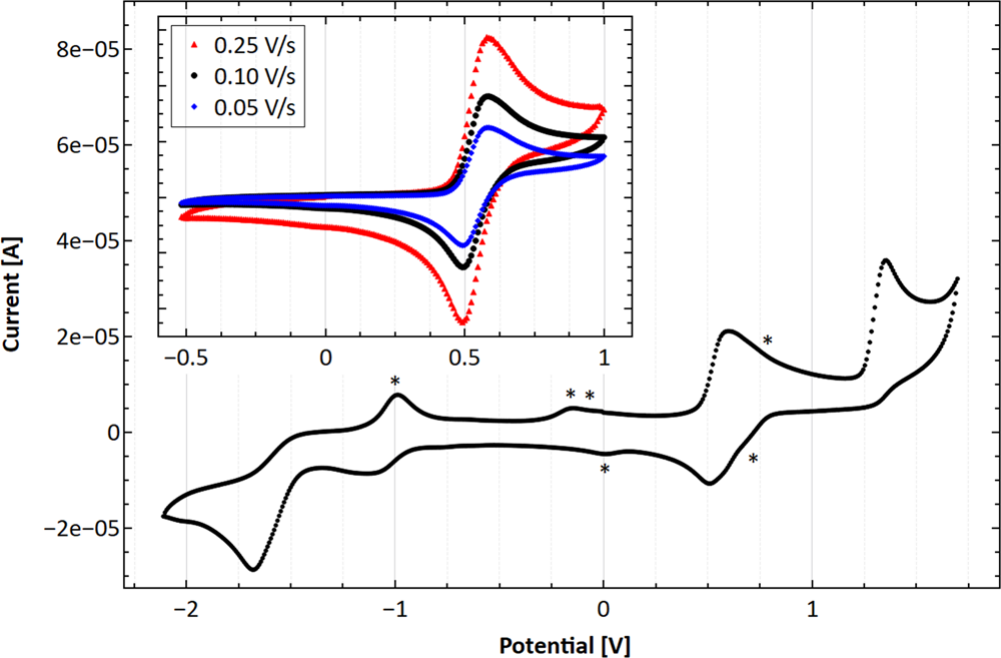
Cyclic voltammograms of the most active complex **4** in a solution of 0.1 M TBAPF_6_ in acetonitrile using a scanning speed of 0.1 V/s and referenced to ferrocene *E*^0^′ (Fc/Fc^+^ = 0.40 V against SCE) as an internal standard. *Signals derived from electrochemically produced decomposition products. Inlet: scan of the fully reversible Fc*/Fc*^+^ redox couple of **4** using scanning speeds between 0.05 and 0.25 V/s.

Ferrocene-containing compounds are well known to exhibit cytotoxicity due to the formation of ROS, due the Fe/Fe^+^ redox couple.^17^ To test that the redox potential is within a biological relevant region, cyclic voltammetry (CV) experiments are conducted on complexes **1**, **2**, **4**, and **7** and their corresponding ferrocenyl β-diketonate ligands **L1**, **L2**, **L4**, and **L7** for reference. All complexes exhibit a rich redox chemistry with several oxidation and reduction peaks in the scanned region between −2.1 and 1.65 V (Table 1, Figure 5, Figures S53–S57, and Tables S17 and S18). The discussion is focused on the metal-based redox processes, and a full analysis of the CV can be found in the Supporting Information.

**Table 1:** Electrochemical Data for Complexes**1**, **2**, **4**, and **7** and Their Respective Ligands; Potentials Are Corrected Using Ferrocene as an Internal Standard with *E*^0^′(Fc/Fc^+^) = 0.40 V against the SCE

compound	*E*^0^′ (Fc*/Fc*^+^) [V]	*E*_P_^Ox^ [V]	*E*_P_^Red^ [V]
**L1**	0.63		–1.98
**L2**	0.63		–1.73
**L4**	0.63		–1.70
**L7**	0.71		–1.42
**1**	0.53	1.29	–1.93/–1.66
**2**	0.54	1.30	–1.62
**4**	0.54	1.32	–1.63
**7**	0.63	1.48	–1.52

All compounds exhibit a reversible single-electron oxidation between 0.53 and 0.71 V, which is assigned to the Fc*/Fc*^+^ redox couple. When compared to ferrocene (0.40 V against SCE), the redox potential is shifted to more positive values due to the electron-withdrawing effect of the β-diketonate functionality, which is in line with previously reported ferrocenyl functionalized ruthenium arene complexes.^26^ Substitution of the methyl group (**1**) with phenyl (**2**) or 2-furan (**4**) has only a negligible effect on the potential in both the ligands and complexes. However, the introduction of a trifluoromethyl moiety (**7**) shifts the potential to more positive values in line with its strongly electron-withdrawing character. The potentials shift by 0.08 and 0.10 V for the ligand and complex, respectively.

The CVs of all complexes exhibit an irreversible oxidation in the region of 1.29–1.48 V with a similar trend as discussed for the ferrocenyl redox couple assigned to the irreversible oxidation of Ru(II) to Ru(III). Therefore, electronic communication between the substituents on the ferrocenyl β-diketonate ligands and the Ru center exists; hence, the electron-withdrawing substituent in **7** increases the redox stability of the Ru(II) center to a higher degree.

Irreversible reductions tentatively assigned to the ferrocenyl β-diketonate ligands are observed in the region between −1.42 and 1.98 V. Since these reductions are too far shifted to lower potentials to be relevant in both normoxic^27^ and hypoxic cellular environments, these are only discussed in the Supporting Information.^28^ A similar conclusion is drawn for the irreversible Ru(II) to Ru(III) oxidation. In contrast, the reversible Fc*/Fc*^+^ redox couples are within a reasonable region to induce ROS, suggesting that such a mode of action might contribute to the overall cytotoxicity. No correlation between the complexes’ CV’s and their cytotoxicity is found, indicating that other possible modes of actions highly likely contribute significantly to the overall toxicity.

### Influence of Hypoxia

Cancer cells have areas with extremely low oxygen concentrations, which is referred to as hypoxia, which leads to a reducing environment inside the cells. This environment is due to the poor formation of new blood vessels during the rapid growth phase of the tumor.^29^ Hypoxic cells are well known to be resistant toward chemotherapy and radiotherapy treatments, leading to great challenges in finding suitable cancer therapeutics.^30^ In particular, reducing environments associated with hypoxia can cause difficulties for transition metals as a change in their oxidation state can lead to a change in their structure, binding mode, cellular drug uptake, and metabolism and even reduce the effectiveness of their cellular mechanism of action or change it completely.^31^

The influence of the oxygen concentration upon the complex’s potency was assessed after 96 h of MTT assay in severe hypoxic conditions (0.1% O_2_). Complexes **4**, **7**, CDDP, and OXA were screened against MIA-PaCa-2 (Figure S14), and all show a decrease in cytotoxicity, including the clinically approved compounds CDDP and OXA. Complex **7** experienced a 4-fold loss of activity from normoxic to hypoxic conditions (IC_50_ values = 11 ± 3 μM, cf. 44 ± 7 μM), while CDDP exhibited a significant loss of activity that was >14-fold (IC_50_ values = 3.6 ± 0.7 μM, cf. >50 μM). All compounds are considered inactive, and this may be due to no accessible reduction available in the cellular environment. Possible reasons for the reduced activity under hypoxic conditions are the absence of a reduction peak close to −0.241 V against the SCE (0 V against the SHE), ruling out a redox-dependent activation as observed for other ruthenium compounds like NAMI-A or KP1019^32^ and no targeting of hypoxia-inducible factors as described previously for Ru(II) arene complexes.^33,34^ A loss of such activity has also been observed for the clinical platinum complexes CDDP and OXA under hypoxic conditions against a range of cell lines.^35−38^

#### DNA Damage via the Comet Assay

**Figure 6:**
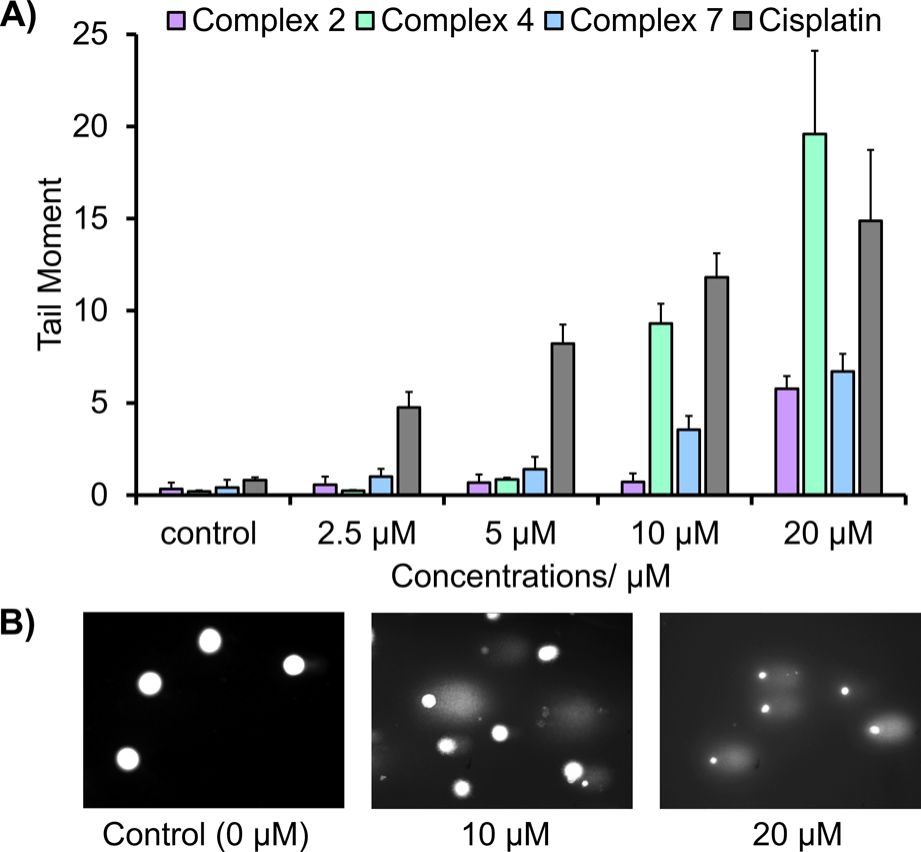
(A) Comet assay analysis of complexes **2**, **4**, and **7** (and cisplatin) when incubated for 48 h with MIA PaCa-2 cells and (B) images of “comets” observed using the comet assay when MIA PaCa-2 cells were incubated for 48 h with complex **4** (IC_50_ = 23 ± 2 μM), showing dose-dependent single-strand breakage (SSB).

As the ICP-MS data showed high accumulation of Fe and Ru inside the cell, interactions with DNA were assessed as a potential mode of action. Complexes **2**, **4**, and **7** were chosen due to their range in cytotoxicity, where complex **2** is moderately active and complexes **4** and **7** have the highest activities. Their ability to induce single-strand breakage (SSB) of DNA with varying concentrations of compound was studied after incubation with MIA PaCa-2 cells for 48 h. After harvesting the cells, quantification of the levels of SSB of DNA were assessed by using the alkaline comet assay. Compounds **2** and **7** only show a small degree of SSB when incubated for 48 h but increase in a dose-dependent manner with respect to an increase in the concentration (Figure 6A). Compound **4**, which has the highest cytotoxicity, shows a significant degree of SSB after 48 h and dose-dependent with respect to an increase in the concentration.

The SSB values complement the IC_50_ values obtained from the 48 h exposure times (Table S15) in which complex **4** exhibits the lowest IC_50_ value and exhibits the highest degree of SSB in DNA. Figure 6B shows an example of microscope images during the scoring of the “comets”. Using the IC_50_ value after 48 h, the following trend in activity was observed: **4** > **7** > **2**, which is the same trend observed in the degree of DNA damage and highlights a strong correlation between the two assays. The same trend is not observed for CDDP, which exhibits low cytotoxicity after 48 h (IC_50_ = 76 ± 3 μM); however, exhibits dose-dependent SSB similar to that of complex **4**. While other mechanisms are likely to be involved, the induction of SSB provides a possible cause of the apoptotic phenotype induced by these compounds. Induction of SSB is common for cisplatin; however, we cannot confirm the modes of action of our compounds without a full in-depth analysis, including double-strand breakage (DSB) and cross-linking assays. Complex **4** shows impressive DNA interactions when compared to the low cellular uptake measured by ICP-MS (Figure 4), highlighting that improvements to its cellular uptake could lead to a significant improvement in cytotoxicity.

### Antimicrobial and Antifungal Agents

**Figure 7:**
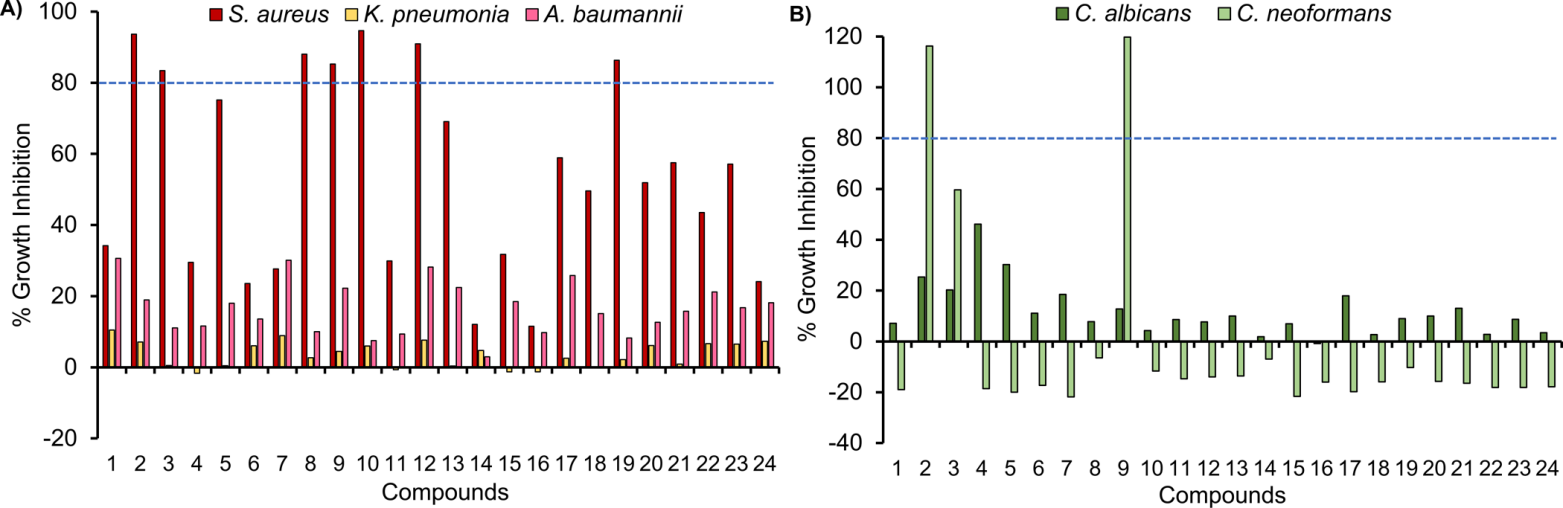
Growth inhibition for complexes **1**–**24** at 32 μg/mL when screened against (A) bacterial strains *S. aureus*, *K. pneumonia*, and *A. baumannii* and (B) antifungal strains *C. albicans* and *C. neoformans*.

Ruthenium and ferrocene compounds are well-documented to have antimicrobial properties,^39−41^ and we previously highlighted the bis(bypyridyl)ruthenium ferrocenyl β-diketonato complexes (Figure 1F) to have moderate to high growth inhibition against *Staphylococcus aureus* (*S. aureus*).^21^ We have screened complexes **1**–**24** for their antibacterial activity against ESKAPE pathogens (Figure 7). This work was kindly conducted by The Community for Antimicrobial Drug Discovery (CO-ADD) at The University of Queensland’s Institute for Molecular Bioscience.^42^

All complexes were initially screened at 32 μg/mL and exhibit significantly high activity toward Gram-positive *S. aureus* (86–95%), moderate to low activity against Gram negative *K. pneumoniae* and *A. baumanni* (Figure 7A) and no activity against Gram negative *E. coli* or *P. aeruginosa* (Table S19). Although there are few distinct trends, generally, the most active complexes against *S. aureus* contain a Fc-acac ligand with neutral inductive aromatic ring systems (**2**, **3**, and **8**–**10**), or increasing the number of halides in the structure (e.g., di-Cl **19** vs mono-Cl **18** and **20**) can increase the activity by approximately 2-fold. Similar trends are observed for the position of the F-substituted complexes, where **17** (*para*) > **15** (*meta*) > **14** (*ortho*) > **16** (di-*meta*). Interestingly, decreasing electronegativity of the halogen atoms F > Cl > Br > I causes a decrease in the activity of the complexes when the halogen atom is located at the *para* position, yet an increase in activity of the complexes when the halogen atom is located at the *meta* position. The opposite observation is true for electron-donating substituents (i.e., R = Me) and suggests that the inductive effects around the aromatic ring may be responsible to some degree in imparting bacterial inhibition properties to the complexes. Complexes that were classified as active underwent HIT confirmation to determine their minimum inhibitory concentration (MIC; parentheses of Table S19), and complexes **2** and **9** are classified as active with MIC values of 16 μg/mL.

When addressing the fungal growth inhibition after incubation with complexes **1**–**24**, complexes **2** and **9** were also the only compounds found to be active against the *C. neoformans* strain, with inhibition concentrations of 116 and 120%, respectively (Figure 7B).Complexes **2**, **3**, **8**–**10**, **12**, and **19** underwent additional HITconfirmation to determine their MIC (parentheses of Table S19), yet they were all inactive with MIC values of >32 μg/mL. The complexes have varying toxicities toward normal kidney cells (Hk, Table S19); however, hemolysis (Hm) results were extremely positive, and all complexes exhibited no potency toward human blood at the maximum tested concentration of 32 μg/mL, which is important for the distribution of these complexes in the bloodstream.

## Conclusions

A library of 24 *p*-cymene Ru(II) complexes, [(*p*-cym)RuCl(Fc-acac)], containing functionalized ferrocenyl β-diketonate ligands (Fc-acac), are reported, including sc-XRD determination for 21 of the complexes. The complexes have been screened against MIA PaCa-2, HCT116 *p53*^+/+^, and ARPE-19 cell lines. The complexes are generally more active toward MIA PaCa-2 than HCT116 *p53*^+/+^ and exhibit no cytotoxicity against the normal cell line (IC_50_ > 100 μM). This is contrary to the clinical platinum drugs CCDP and OXA, which remain cytotoxic; IC_50_ values of 6 ± 1 μM and 6 ± 3 μM, respectively. Complex **4** (R = 2-furan) exhibits the highest cytotoxicity of this library (IC_50_ = 8 ± 2 μM) with a selectivity index (SI) of 12.5 against MIA PaCa-2.

UV–vis and NMR studies highlight the complexes’ change over 96 h, and while UV–vis data show changes to the Ru–Cl bond, the NMR studies show decomposition to free *p*-cymene and free Cp. This process is faster in the presence of water but slowed when 100 mM NaCl is added. Shortened-time exposure MTT and ICP-MS were used to study the uptake of the compounds into MIA PaCa-2 after 48 h. The highest uptake is not observed for the most active complexes and could be due to a lack of compound stability.

Cyclic voltammetry studies are conducted revealing a reversible Fc*/Fc*^+^ redox couple between 0.53 and 0.63 V (against SCE), indicating that the formation of ROS is a reasonable mode of action. A correlation between the redox potential and cytotoxicity is not observed pointing out that further studies are necessary to assess the relevance of ROS to the overall toxicity. Complexes were also tested under severely hypoxic (0.1% O_2_) conditions against MIA PaCa-2; however, like CDDP, these complexes decrease in activity and this could be due to inaccessible reduction potentials. Complexes **2**, **4**, and **7** were further tested for their ability to induce single-strand breakage (SSB) of DNA within MIA PaCa-2 cells with complex **4** exhibiting the highest degree of damage, which is dose-dependent with respect to the concentration. The work highlights that complex **4** exhibits an impressive amount of DNA damage despite the lower concentration of intracellular metal which was observed by ICP-MS. Further modifications to this complex to improve cell uptake could therefore lead to significantly enhanced cytotoxicity.

Finally, the growth inhibition of bacterial and fungal strains, human embryonic kidney (HEK-293) and hemolysis assays were conducted. The complexes have increased activity toward Gram-positive strains, for example, *S. aureus* (83–95%), but no or low activity against Gram-negative strains. While the complexes showed varying degrees of cytotoxicity toward HEK-293 cells, they are all non-toxic towards human blood (HC_10_ > 32 μg/mL), which is important for the distribution of these complexes in the bloodstream.

## Experimental Section

The general methods, instrumentation, synthesis/characterization of the ligands, X-ray crystallography, and biological assays can be found in the Supporting Information. Ligand **L6** (±0.54%) and complexes **8** (±0.59%), **16** (±0.60%), **18** (±0.79), and **19** (±0.43) have elemental analysis values slightly higher than expected, and although these results are outside the range viewed as establishing analytical purity, they are provided to illustrate the best values obtained to date. To support the results, we have provided high-resolution mass spectrometry data, which are within range, and single-crystal X-ray diffraction analysis for complexes **16**, **18**, and **19** to prove that products were achieved successfully.

### General Procedure for the Ruthenium Complexes

A functionalized ferrocenyl ligand (2 equiv) was dissolved in dichloromethane (20 mL) followed by addition of triethylamine (2 equiv) and [*p-*cymRuCl_2_]_2_ (1 equiv). The mixture was stirred at room temperature overnight. The solvent was removed in vacuo and purified by column chromatography using 3:2 *v*/*v* petrol/ethyl acetate to yield orange solids.^14^

#### Complex **1**

Yield: 0.14 g, 68%. ^1^H NMR (500 MHz, (CD_3_)_2_CO, δ); 5.47 (d, 1H, ^3^*J*(^1^H–^1^H) = 6.0 Hz, *p-*cymene ArC–H), 5.45 (d, 1H, ^3^*J*(^1^H–^1^H) = 6.0 Hz, *p*-cymene ArC–H), 5.32 (s, 1H, methine −COCHCO−), 5.12 (d, 2H, ^3^*J*(^1^H–^1^H) = 6.0 Hz, *p*-cymene ArC–H), 4.75 (t, 1H, ^3^*J*(^1^H–^1^H) = 1.4 Hz, −CC_4_H_4_), 4.51 (t, 1H, ^3^*J*(^1^H–^1^H) = 1.4 Hz, Cp −CC_4_H_4_), 4.24 (q, 1H, ^3^*J*(^1^H–^1^H) = 2.5, 1.3 Hz, Cp −CC_4_H_4_), 4.20 (q, 1H, ^3^*J*(^1^H–^1^H) = 2.5, 1.3 Hz, Cp −CC_4_H_4_), 4.07 (s, 5H, Cp −C_5_H_5_), 2.83 (sept, 1H, ^3^*J*(^1^H–^1^H) = 6.9 Hz, *p*-cymene CH(CH_3_)_2_, H_8_), 2.07 (s, 3H, *p*-cymene Ar–CCH_3_), 1.79 (s, 3H, −COCH_3_), 1.29 (dd, 6H, ^3^*J*(^1^H–^1^H) = 6.9, ^4^*J*(^1^H–^1^H) = 2.3 Hz, *p*-cymene −C(CH_3_)_2_). ^13^C{^1^H} NMR (125 MHz, (CD_3_)_2_CO, δ); 184.9 (Q C–O), 184.6 (Q C–O), 99.2 (Q *p*-cymene −CCH(CH_3_)_2_), 97.7 (Q *p*-cymene −C(CH_3_), 95.9 (methine −COCHCO−), 84.6 (*p*-cymene Ar–CH), 84.1 (*p*-cymene Ar–CH), 81.7 (Q Cp −CC_4_H_4_), 79.6 (*p*-cymene Ar–CH), 71.3 (Cp −CC_4_H_4_), 71.3 (Cp −CC_4_H_4_), 70.8 (Cp −C_5_H_5_), 69.8 (Cp −CC_4_H_4_), 68.4 (Cp −CC_4_H_4_), 31.7 (*p*-cymene −CH(CH_3_)_2_), 27.5 (−COCH_3_) 22.6 (*p*-cymene −CH(CH_3_)_2_), 17.7 (*p*-cymene −C(CH_3_)). Analysis calculated for C_24_H_27_ClFeO_2_Ru: C 53.40, H 5.04, Cl 6.57%. Found: C 53.40, H 5.10, Cl 6.40%. HR-MS [ES^+^] calculated for C_24_H_27_ClFeO_2_Ru: 540.009. Found: 505.042 [MH^+^]-Cl.

#### Complex **2**

Yield: 0.21 g, 89%. ^1^H NMR (500 MHz, (CD_3_)_2_CO, δ); 7.84 (br. d, 2H, ^3^*J*(^1^H–^1^H) = 7.3 Hz, *ortho* ArC–H), 7.33 (t, 1H, ^3^*J*(^1^H–^1^H) = 7.3 Hz, *para* ArC–H), 7.28 (t, 2H, ^3^*J*(^1^H–^1^H) = 7.3 Hz, *ortho* ArC–H), 6.04 (s, 1H, methine −COCHCO−), 5.55 (d, 2H, ^3^*J*(^1^H–^1^H) = 6.4 Hz, *p*-cymene ArC–H), 5.24 (dd, 2H, ^3^*J*(^1^H–^1^H) = 6.0 Hz, ^4^*J*(^1^H–^1^H) = 1.8 Hz, *p*-cymene ArC–H), 4.83 (t, 1H, ^3^*J*(^1^H–^1^H) = 1.2 Hz, −CC_4_H_4_), 4.73 (t, 1H, ^3^*J*(^1^H–^1^H) = 1.2 Hz, −CC_4_H_4_), 4.32 (br. q, 1H, ^3^*J*(^1^H–^1^H) = 2.2, 1.4 Hz, −CC_4_H_4_), 4.28 (br. q, 1H, ^3^*J*(^1^H–^1^H) = 2.3, 1.4 Hz, −CC_4_H_4_), 4.11 (s, 5H, −C_5_H_5_), 2.91 (sept, 1H, ^3^*J*(^1^H–^1^H) = 6.9 Hz, *p*-cymene −CH(CH_3_)_2_), 2.16 (s, 3H, *p-*cymene Ar–CCH_3_), 1.34 (d, 6H, ^3^*J*(^1^H–^1^H) = 7.3 Hz, *p-*cymene −CH(CH_3_)_2_). ^13^C{^1^H} NMR (125 MHz, (CD_3_)_2_CO, δ); 186.8 (Q C–O), 178.4 (Q C–O), 140.5 (Q Ar–C), 131.2 (*ortho* Ar–CH), 128.9 (*meta* Ar–CH), 127.9 (*para* Ar–CH), 99.4 (Q *p*-cymene −CCH(CH_3_)_2_), 97.8 (Q *p*-cymene −C(CH_3_)), 93.4 (methine −COCHCO−), 84.6 (*p*-cymene Ar–CH), 84.5 (*p*-cymene Ar–CH), 82.1 (Q Cp −CC_4_H_4_), 80.0 (*p*-cymene Ar–CH), 80.0 (*p*-cymene Ar–CH), 71.7 (Cp −CC_4_H_4_), 71.6 (Cp −CC_4_H_4_), 70.9 (Cp −C_5_H_5_), 69.9 (Cp −CC_4_H_4_), 68.7 (Cp −CC_4_H_4_), 31.7 (*p*-cymene −CH(CH_3_)_2_), 22.7 (*p*-cymene −CH(CH_3_)_2_), 22.6 (*p*-cymene −CH(CH_3_)_2_), 17.9 (*p*-cymene −C(CH_3_)). Analysis calculated for C_29_H_29_ClFeO_2_Ru: C 57.87, H 4.86, Cl 5.89%. Found: C 57.70, H 5.20, Cl 5.75%. HR-MS [ES^+^] calculated for C_29_H_29_ClFeO_2_Ru: 602.025. Found: 567.058 [MH^+^]-Cl.

#### Complex **3**

Yield: 0.21 g, 82%. ^1^H NMR(500 MHz, (CD_3_)_2_CO, δ); 8.54 (d, 1H, ^3^*J*(^1^H–^1^H) = 7.8 Hz, NpC_2_–H), 7.82 (d, 1H, ^3^*J*(^1^H–^1^H) = 8.0 Hz, NpC_3_–H), 7.78 (dd, 1H, ^3^*J*(^1^H–^1^H) = 7.1 Hz, ^4^*J*(^1^H–^1^H) = 2.1 Hz, NpC_3_–H), 7.45 (d, 1H, ^3^*J*(^1^H–^1^H) = 7.1 Hz, NpC_9_–H), 7.37 (m, 3H, NpC_6–8_–H), 5.72 (s, 1H, methine −COCHCO−), 5.57 (d, 1H, ^3^*J*(^1^H–^1^H) = 5.7 Hz, *p-*cymene ArC–H), 5.53 (d, 1H, ^3^*J*(^1^H–^1^H) = 5.9 Hz, *p-*cymene ArC–H), 5.25 (t, 2H, ^3^*J*(^1^H–^1^H) = 4.9 Hz, *p-*cymene ArC–H), 4.83 (d, 1H, ^3^*J*(^1^H–^1^H) = 0.7 Hz, Cp −CC_4_H_4_), 4.62 (d, 1H, ^3^*J*(^1^H–^1^H) = 0.9 Hz, Cp −CC_4_H_4_), 4.33 (m, 1H, Cp −CC_4_H_4_), 4.27 (m, 1H, Cp −CC_4_H_4_), 4.14 (s, 5H, Cp −C_5_H_5_), 2.86 (sept, 1H, ^3^*J*(^1^H–^1^H) = 6.9 Hz, *p-*cymene −CH(CH_3_)_2_), 2.09 (s, 3H, *p-*cymene −C(CH_3_)), 1.30 (t, 6H, ^3^*J*(^1^H–^1^H) = 6.7 Hz, *p-*cymene −CH(CH_3_)_2_). ^13^C{^1^H} NMR (125 MHz, (CD_3_)_2_CO, δ); 186.9 (Q C–O), 176.0 (Q C–O), 156.4 (Q Np–C_1_), 140.5 (Q Np–C_10_), 134.9 (Q Np–C_5_), 132.0 (Np–C_2_H), 130.2 (Np–C_3_H), 128.6 (Np–C_4_H), 128.4 (Np–C_9_H), 126.8 (Np–C_6_H), 125.7 (Np–C_8_H), 125.6 (Np–C_7_H), 99.7 (Q *p-*cymene, −CCH(CH_3_)_2_), 98.3 (methine −COCHCO−), 97.7 (Q *p-*cymene, −C(CH_3_)), 84.4 (*p-*cymene Ar–CH), 84.2 (*p-*cymene Ar–CH), 81.5 (Q Cp −CC_4_H_4_), 80.1 (*p-*cymene Ar–CH), 80.1 (*p-*cymene Ar–CH), 71.9 (Cp −CC_4_H_4_), 71.9 (Cp −CC_4_H_4_), 71.0 (Cp −C_5_H_5_), 69.9 (Cp −CC_4_H_4_), 68.8 (Cp −CC_4_H_4_), 31.6 (*p-*cymene −CCH(CH_3_)_2_), 22.7 (*p-*cymene −CCH(CH_3_)_2_), 22.6 (*p-*cymene −CCH(CH_3_)_2_), 17.8 (*p-*cymene −C(CH_3_)). Analysis calculated for C_33_H_31_ClFeO_2_Ru: C 60.79, H 4.79, Cl 5.44%. Found: C 60.70, H 4.90, Cl 5.20%. HR-MS [ES^+^] calculated for C_33_H_31_ClFeO_2_Ru: 652.041. Found: 617.074 [MH^+^]-Cl.

#### Complex **4**

Yield: 0.20 g, 86%. ^1^H NMR (500 MHz, (CD_3_)_2_CO, δ); 7.53 (br. d, 1H, ^3^*J*(^1^H–^1^H) = 0.9 Hz, Furan −C(*O*)C_3_H_3_), 6.96 (br. d, 1H, ^3^*J*(^1^H–^1^H) = 3.4 Hz, Furan −C(*O*)C_3_H_3_), 6.44 (dd, 1H, ^3^*J*(^1^H–^1^H) = 3.7 Hz, ^4^*J*(^1^H–^1^H) = 1.6 Hz, Furan −C(*O*)C_3_H_3_), 5.92 (s, 1H, methine −COCHCO−), 5.53 (t, 2H, ^3^*J*(^1^H–^1^H) = 6.3 Hz, *p*-cymene ArC–H), 5.20 (t, 2H, ^3^*J*(^1^H–^1^H) = 5.7 Hz, *p*-cymene ArC–H), 4.79 (br. t, 1H, ^3^*J*(^1^H–^1^H) = 1.2 Hz, Cp −CC_4_H_4_), 4.60 (br. t, 1H, ^3^*J*(^1^H–^1^H) = 1.2 Hz, Cp −CC_4_H_4_), 4.32 (br. q, 1H, ^3^*J*(^1^H–^1^H) = 2.5, 1.2 Hz, Cp −CC_4_H_4_), 4.28 (br. q, 1H, ^3^*J*(^1^H–^1^H) = 2.5 Hz, ^4^*J*(^1^H–^1^H) = 1.2 Hz, Cp −CC_4_H_4_), 4.11 (s, 5H, Cp −C_5_H_5_), 2.88 (sept, 1H, ^3^*J*(^1^H–^1^H) = 6.9 Hz, *p*-cymene −CH(CH_3_)_2_, H_8_), 2.14 (s, 3H, *p*-cymene −C(CH_3_)), 1.33 (d, 6H, ^3^*J*(^1^H–^1^H) = 6.9 Hz, *p*-cymene −CH(CH_3_)_2_). ^13^C{^1^H} NMR (125 MHz, (CD_3_)_2_CO, δ); 186.6 (Q C–O), 168.6 (Q C–O), 163.6 (Q Furan −C(*O*)C_3_H_3_), 145.0 (Furan −C(*O*)C_3_H_3_), 113.3 (Furan −C(*O*)C_3_H_3_), 112.9 (Furan −C(*O*)C_3_H_3_), 99.2 (Q *p*-cymene −CCH(CH_3_)_2_), 97.9 (Q *p*-cymene, −C(CH_3_)), 92.3 (methine −COCHCO−), 84.8 (*p*-cymene Ar–CH), 84.5 (*p*-cymene Ar–CH), 81.9 (Q Cp −CC_4_H_4_), 79.9 (*p*-cymene Ar–CH_7_), 79.8 (*p*-cymene Ar–CH), 71.7 (Cp −CC_4_H_4_), 71.7 (Cp −CC_4_H_4_), 70.9 (Cp −C_5_H_5_), 69.9 (Cp −CC_4_H_4_), 68.6 (Cp −CC_4_H_4_), 31.7 (*p*-cymene −CCH(CH_3_)_2_), 22.7 (*p*-cymene −CCH(CH_3_)_2_), 22.6 (*p*-cymene −CCH(CH_3_)_2_), 17.7 (*p*-cymene −C(CH_3_)). Analysis calculated for C_27_H_27_ClFeO_3_Ru: C 54.79, H 4.60, Cl 5.99%. Found: C 55.05, H 4.60 Cl 5.99%. HR-MS [ES^+^] calculated for C_27_H_27_ClFeO_3_Ru: 592.004. Found: 557.036 [MH^+^]-Cl.

#### Complex **5**

Yield: 0.19 g, 81%. ^1^H NMR (500 MHz, (CD_3_)_2_CO, δ); 8.19 (s, 1H, Furan −CH(*O*)C_2_H_2_), 7.60 (t, 1H, ^3^*J*(^1^H–^1^H) = 1.6 Hz, Furan −CH(*O*)C_2_H_2_), 6.90 (d, 1H, ^3^*J*(^1^H–^1^H) = 1.2 Hz, Furan −CH(*O*)C_2_H_2_), 5.97 (s, 1H, methine −COCHCO−), 5.68 (t, 2H, ^3^*J*(^1^H–^1^H) = 6.1 Hz, *p-*cymene ArC–H), 5.35 (t, 2H, ^3^*J*(^1^H–^1^H) = 5.3 Hz, *p-*cymene ArC–H), 4.96 (d, 1H, ^3^*J*(^1^H–^1^H) = 1.2 Hz, −CC_4_H_4_), 4.84 (d, 1H, ^3^*J*(^1^H–^1^H) = 1.2 Hz, −CC_4_H_4_), 4.44 (m, 1H, −CC_4_H_4_), 4.40 (m, 1H, −CC_4_H_4_), 4.25 (s, 5H, −C_5_H_5_), 3.04 (sept, 1H, ^3^*J*(^1^H–^1^H) = 6.9 Hz, *p-*cymene −CCH(CH_3_)_2_), 2.29 (s, 3H, *p-*cymene −C(CH_3_)), 1.49 (dd, 6H, ^3^*J*(^1^H–^1^H) = 6.9 Hz, ^4^*J*(^1^H–^1^H) = 0.9 Hz, *p-*cymene −CCH(CH_3_)_2_). ^13^C{^1^H} NMR (125 MHz, (CD_3_)_2_CO, δ); 186.0 (Q C–O), 173.6 (Q C–O), 145.0 (Furan −CCH(*O*)C_2_H_2_), 144.4 (Furan −CCH(*O*)C_2_H_2_), 128.8 (Q Furan −CCH(*O*)C_2_H_2_), 109.8 (Furan −CCH(*O*)C_2_H_2_), 99.2 (Q *p*-cymene −CCH(CH_3_)_2_), 97.8 (Q *p*-cymene −C(CH_3_)), 94.0 (methine −COCHCO−), 84.7 (*p*-cymene Ar–CH), 84.5 (*p*-cymene Ar–CH), 82.0 (Q Cp −CC_4_H_4_), 79.9 (*p*-cymene Ar–CH), 79.8 (*p*-cymene Ar–CH), 71.5 (Cp −CC_4_H_4_), 71.5 (Cp −CC_4_H_4_), 70.9 (Cp −C_5_H_5_), 69.8 (Cp −CC_4_H_4_), 68.6 (Cp −CC_4_H_4_), 31.7 (*p*-cymene −CCH(CH_3_)_2_), 22.7 (*p*-cymene −CCH(CH_3_)_2_), 22.6 (*p*-cymene −CCH(CH_3_)_2_), 17.7 (*p*-cymene −C(CH_3_)). Analysis calculated for C_27_H_27_ClFeO_3_Ru: C 54.79, H 4.60, Cl 5.99%. Found: C 54.72, H 4.55, Cl 6.01%. HR-MS [ES^+^] calculated for C_27_H_27_ClFeO_3_Ru: 592.004. Found: 557.043 [MH^+^]-Cl.

#### Complex **6**

Yield: 0.17 g, 77%. ^1^H NMR (500 MHz, (CD_3_)_2_CO, δ); 5.86 (t, 1H, ^4^*J*(^1^H–^19^F) = 5.2 Hz, methine −COCHCO−), 5.57 (br. t, 2H, ^3^*J*(^1^H–^1^H) = 6.2 Hz, *p-*cymene ArC–H), 5.56 (br. s, H, −CHF_2_), 5.22 (t, 2H, ^3^*J*(^1^H–^1^H) = 5.6 Hz, *p-*cymene ArC–H), 4.80 (br. s, 1H, −CC_4_H_4_), 4.62 (br. s, 1H, −CC_4_H_4_), 4.40 (br. s, 1H, −CC_4_H_4_), 4.35 (br. s, 1H, −CC_4_H_4_), 4.11 (s, 5H, −C_5_H_5_), 2.71 (sept, 1H, ^3^*J*(^1^H–^1^H) = 5.9 Hz, *p-*cymene −CCH(CH_3_)_2_), 2.09 (s, 3H, *p-*cymene −C(CH_3_)), 1.30 (t, 6H, ^3^*J*(^1^H–^1^H) = 5.9 Hz, *p-*cymene −CCH(CH_3_)_2_). ^13^C{^1^H} NMR (125 MHz, (CD_3_)_2_CO, δ); 190.1 (Q C–O), 172.8 (t, Q C–O, ^2^*J*(^13^C–^19^F) = 22.1 Hz), 112.2 (t, -CHF_2_, ^1^*J*(^13^C–^19^F) = 246.3 Hz), 99.5 (Q *p*-cymene −CCH(CH_3_)_2_), 98.2 (Q *p*-cymene −C(CH_3_)), 92.6 (methine −COCHCO−), 84.9 (*p*-cymene Ar–CH), 84.2 (*p*-cymene Ar–CH), 80.5 (Q Cp −CC_4_H_4_), 79.9 (*p*-cymene Ar–CH), 79.5 (*p*-cymene Ar–CH), 72.7 (Cp −CC_4_H_4_), 72.6 (Cp −CC_4_H_4_), 71.2 (Cp −C_5_H_5_), 70.1 (Cp −CC_4_H_4_), 69.1 (Cp −CC_4_H_4_), 31.7 (*p*-cymene −CCH(CH_3_)_2_), 22.6 (*p*-cymene −CCH(CH_3_)_2_), 22.5 (*p*-cymene −CCH(CH_3_)_2_), 17.7 (*p*-cymene −C(CH_3_)). Analysis calculated for C_24_H_25_ClF_2_FeO_2_Ru: C 50.06, H 4.38, Cl 6.16%. Found: C 50.30, H 4.40, Cl 6.30%. HR-MS [ES^+^] calculated for C_24_H_25_ClF_2_FeO_2_Ru: 575.990. Found: 541.023 [MH^+^]-Cl.

#### Complex **7**

Yield: 0.20 g, 85%. ^1^H NMR (500 MHz, (CD_3_)_2_CO, δ); 5.63 (s, 1H, methine −COCHCO−), 5.61 (t, 2H, ^3^*J*(^1^H–^1^H) = 5.5 Hz, *p-*cymene ArC–H), 5.30 (d, 1H, ^3^*J*(^1^H–^1^H) = 5.5 Hz, *p-*cymene ArC–H), 5.27 (d, 1H, *J* 5.5 Hz, *p-*cymene ArC–H), 4.83 (t, 1H, ^3^*J*(^1^H–^1^H) = 1.4 Hz, −CC_4_H_4_), 4.68 (t, 1H, ^3^*J*(^1^H–^1^H) = 1.4 Hz, −CC_4_H_4_), 4.46 (m, 1H, −CC_4_H_4_), 4.41 (m, 1H, −CC_4_H_4_), 4.13 (s, 5H, −C_5_H_5_), 2.83 (sept, 1H, ^3^*J*(^1^H–^1^H) = 6.9 Hz, *p-*cymene −CCH(CH_3_)_2_), 2.10 (s, 3H, *p-*cymene −C(CH_3_)), 1.17 (d, 6H, ^3^*J*(^1^H–^1^H) = 7.3 Hz, *p-*cymene −CCH(CH_3_)_2_). ^13^C{^1^H} (125 MHz, (CD_3_)_2_CO, δ); 218.9 (Q C–O), 191.8 (Q C–O), 99.5 (Q *p*-cymene −CCH(CH_3_)_2_), 98.3 (Q *p*-cymene −C(CH_3_)), 92.2 (methine −COCHCO−), 84.8 (*p*-cymene Ar–CH), 84.2 (*p*-cymene Ar–CH), 80.1 (Q Cp −CC_4_H_4_), 79.5 (*p*-cymene Ar–CH), 79.5 (*p*-cymene Ar–CH), 73.5 (Cp −CC_4_H_4_), 73.1 (Cp −CC_4_H_4_), 71.3 (Cp −C_5_H_5_), 70.9 (Cp −CC_4_H_4_), 67.5 (Cp −CC_4_H_4_), 31.8 (*p*-cymene −CCH(CH_3_)), 22.5 (*p*-cymene −CCH(CH_3_)), 22.5 (*p*-cymene −CCH(CH_3_)_2_), 17.7 (*p*-cymene −C(CH_3_)). Analysis calculated for C_24_H_24_ClF_3_FeO_2_Ru: C 48.54, H 4.07, Cl 5.97%. Found: C 48.90, H 4.10, Cl 6.00%. HR-MS [ES^+^] calculated for C_24_H_24_ClF_3_FeO_2_Ru: 593.981. Found: 559.023 [MH^+^]-Cl.

#### Complex **8**

Yield: 0.23 g, 93%. ^1^H NMR (500 MHz, (CD_3_)_2_CO, δ); 7.79 (s, 1H, *ortho* ArC–H), 7.75 (m, 1H, *ortho* ArC–H), 7.29 (m, 2H, *meta* and *para* ArC–H), 6.15 (s, 1H, methine −COCHCO−), 5.68 (dt, 2H, ^3^*J*(^1^H–^1^H) = 4.9, ^4^*J*(^1^H–^1^H) = 1.5 Hz, *p-*cymene ArC–H), 5.36 (dt, 2H, ^3^*J*(^1^H–^1^H) = 4.9 Hz, ^4^*J*(^1^H–^1^H) = 1.5 Hz, *p-*cymene ArC–H), 4.96 (t, 1H, ^3^*J*(^1^H–^1^H) = 1.2 Hz, −CC_4_H_4_), 4.84 (t, 1H, ^3^*J*(^1^H–^1^H) = 1.2 Hz, −CC_4_H_4_), 4.44 (m, 1H, −CC_4_H_4_), 4.40 (m, 1H, −CC_4_H_4_), 4.24 (s, 5H, −C_5_H_5_), 3.04 (sept, 1H, ^3^*J*(^1^H–^1^H) = 6.9 Hz, *p-*cymene −CCH(CH_3_)_2_, H_8_), 2.42 (s, 3H, Ar–CCH_3_), 2.29 (s, 3H, *p-*cymene −C(CH_3_)), 1.47 (d, 6H, ^3^*J*(^1^H–^1^H) = 7.1 Hz, *p-*cymene −CCH(CH_3_)_2_). ^13^C{^1^H} NMR (125 MHz, (CD_3_)_2_CO, δ); 186.6 (Q C–O), 178.6 (Q C–O), 140.5 (Q Ar–C), 138.4 (Q Ar–C(CH_3_)), 131.8 (*ortho* Ar–CH), 128.8 (*ortho* Ar–CH), 128.5 (*para* Ar–CH), 125.1 (*ortho* Ar–CH), 99.4 (Q *p*-cymene, −CCH(CH_3_)_2_), 97.8 (Q *p*-cymene −C(CH_3_)), 93.4 (methine −COCHCO−), 84.6 (*p*-cymene Ar–CH), 84.5 (*p*-cymene Ar–CH), 82.1 (Q Cp −CC_4_H_5_), 80.0 (*p*-cymene Ar–CH), 80.0 (*p*-cymene Ar–CH), 71.6 (Cp −CC_4_H_4_), 71.6 (Cp −CC_4_H_4_), 70.9 (Cp −C_5_H_5_), 69.9 (Cp −CC_4_H_4_), 68.7 (Cp −CC_4_H_4_), 31.7 (*p*-cymene −CCH(CH_3_)_2_), 22.7 (*p*-cymene −CCH(CH_3_)_2_), 22.6 (*p*-cymene −CCH(CH_3_)_2_), 21.5 (*meta* Ar–C(CH_3_)), 17.8 (*p*-cymene −C(CH_3_)). Analysis calculated for C_30_H_31_ClFeO_2_Ru: C 58.50, H 5.07, Cl 5.76%. Found: C 57.91, H 5.06, Cl 5.82%. HR-MS [ES^+^] calculated for C_30_H_31_ClFeO_2_Ru: 616.041. Found: 581.072 [MH^+^]-Cl.

#### Complex **9**

Yield: 0.20 g, 80%; ^1^H NMR (500 MHz, (CD_3_)_2_CO, δ); 7.45 (s, 2H, *ortho* ArC–H), 6.98 (s, 1H, *para* ArC–H), 6.01 (s, 1H, methine −COCHCH−), 5.54 (t, 2H, ^3^*J*(^1^H–^1^H) = 4.0 Hz, *p*-cymene ArC–H), 5.22 (d, 2H, ^3^*J*(^1^H–^1^H) = 4.4 Hz, *p-*cymene ArC–H), 4.82 (br. s, 1H, −CC_4_H_4_), 4.69 (br. s, 1H, −CC_4_H_4_), 4.30 (br. s, 1H, −CC_4_H_4_), 4.26 (br. s, 1H, −CC_4_H_4_), 4.10 (s, 5H, −C_5_H_5_), 2.90 (sept, 1H, ^3^*J*(^1^H–^1^H) = 6.9 Hz, *p-*cymene −CCH(CH_3_)_2_, H_8_), 2.21 (s, 6H, *meta* Ar–C(CH_3_)), 2.16 (s, 3H, *p-*cymene −C(CH_3_)), 1.34 (d, 6H, ^3^*J*(^1^H–^1^H) = 6.9 Hz, *p-*cymene −CCH(CH_3_)_2_). ^13^C{^1^H} NMR (125 MHz, (CD_3_)_2_CO, δ); 185.5 (Q C–O), 178.0 (Q C–O), 139.6 (Q Ar–C), 137.4 (Q *meta* Ar–C), 131.7 (*para* Ar–CH), 124.9 (*ortho* Ar–CH), 124.5 (*ortho* Ar–CH), 98.5 (Q *p*-cymene −CCH(CH_3_)_2_), 96.9 (Q *p*-cymene −C(CH_3_)), 92.5 (methine −COCHCO−), 83.8 (*p*-cymene Ar–CH), 83.6 (*p*-cymene Ar–CH), 81.3 (Q Cp −CC_4_H_4_), 79.1 (*p*-cymene Ar–CH), 79.0 (*p*-cymene Ar–CH), 70.7 (Cp −CC_4_H_4_), 70.6 (Cp −CC_4_H_4_), 70.0 (Cp −C_5_H_5_), 69.0 (Cp −CC_4_H_4_), 67.8 (Cp −CC_4_H_4_), 30.8 (*p*-cymene −CCH(CH_3_)_2_), 22.7 (*p*-cymene −CCH(CH_3_)_2_), 22.6 (*p*-cymene −CCH(CH_3_)_2_), 20.6 (*meta* Ar–C(CH_3_)), 17.9 (*p*-cymene −C(CH_3_)). Analysis calculated for C_31_H_33_ClFeO_2_Ru: C 59.10, H 5.28, Cl 5.63%. Found: C 59.30, H 5.30, Cl 5.50%. HR-MS [ES^+^] calculated for C_31_H_33_ClFeO_2_Ru: 630.056. Found: 595.090 [MH^+^]-Cl.

#### Complex **10**

Yield: 0.18 g, 75%. ^1^H NMR (500 MHz, (CD_3_)_2_CO, δ); 7.74 (d, 2H, ^3^*J*(^1^H–^1^H) = 8.3 Hz, *ortho* ArC–H), 7.10 (d, 2H, ^3^*J*(^1^H–^1^H) = 7.8 Hz, *meta* ArC–H), 6.02 (s, 1H, methine −COCHCO−), 5.54 (br. d, 2H, ^3^*J*(^1^H–^1^H) = 6.0 Hz, *p-*cymene ArC–H), 5.22 (d, 2H, ^3^*J*(^1^H–^1^H) = 6.0 Hz, *p-*cymene ArC–H), 4.82 (t, 1H, ^3^*J*(^1^H–^1^H) = 1.2 Hz, Cp −CC_4_H_4_), 4.71 (t, 1H, ^3^*J*(^1^H–^1^H) = 1.2 Hz, Cp −CC_4_H_4_), 4.30 (m, 1H, Cp −CC_4_H_4_), 4.27 (m, 1H, Cp −CC_4_H_4_), 4.11 (s, 5H, Cp −C_5_H_5_), 2.90 (sept, 1H, ^3^*J*(^1^H–^1^H) = 6.9 Hz, *p-*cymene −CCH(CH_3_)_2_), 2.24 (s, 3H, *para* Ar–C(CH_3_)), 2.16 (s, 3H, *p-*cymene −C(CH_3_)), 1.34 (d, 6H, ^3^*J*(^1^H–^1^H) = 6.9 Hz, *p-*cymene −CCH(CH_3_)_2_). ^13^C{^1^H} (125 MHz, (CD_3_)_2_CO, δ); 186.4 (Q C–O), 178.4 (Q C–O), 141.4 (Q Ar–C), 137.7 (Q *para* Ar–C), 129.6 (*ortho* Ar–CH), 127.9 (*meta* Ar–CH), 99.4 (Q *p*-cymene −CCH(CH_3_)_2_), 97.8 (Q *p*-cymene −C(CH_3_)), 93.0 (methine −COCHCO−), 84.7 (*p*-cymene Ar–CH), 84.5 (*p*-cymene Ar–CH), 82.2 (Q Cp −CC_4_H_4_), 79.9 (*p*-cymene Ar–CH), 71.6 (Cp −CC_4_H_4_), 71.5 (Cp −CC_4_H_4_), 70.9 (Cp −C_5_H_5_), 69.9 (Cp −CC_4_H_4_), 68.6 (Cp −CC_4_H_4_), 31.7 (*p*-cymene −CCH(CH_3_)_2_), 22.7 (*p*-cymene −CCH(CH_3_)_2_), 22.6 (*p*-cymene −CCH(CH_3_)_2_), 21.4 (*para* Ar–C(CH_3_)), 17.8 (*p*-cymene −C(CH_3_)). Analysis calculated for C_30_H_31_ClFeO_2_Ru: C 58.50, H 5.07, Cl 5.76%. Found: C 58.60, H 5.10, Cl 5.80%. HR-MS [ES^+^] calculated for C_30_H_31_ClFeO_2_Ru: 616.041. Found: 581.072 [MH^+^]-Cl.

#### Complex **11**

Yield: 0.20 g, 80%. ^1^H NMR (500 MHz, (CD_3_)_2_CO, δ); 7.55 (s, 1H, *ortho* ArC–H), 7.54 (d, 1H, ^3^*J*(^1^H–^1^H) = 7.8 Hz, *ortho* ArC–H), 7.34 (t, 1H, ^3^*J*(^1^H–^1^H) = 8.1 Hz, *meta* ArC–H), 7.06 (dt, 1H, ^3^*J*(^1^H–^1^H) = 7.3 Hz, ^4^*J*(^1^H–^1^H) = 1.7 Hz, *para* ArC–H), 6.17 (s, 1H, −COCHCO−), 5.72 (q, 2H, ^3^*J*(^1^H–^1^H) = 2.4 Hz, *p-*cymene ArC–H), 5.39 (q, 2H, ^3^*J*(^1^H–^1^H) = 2.4 Hz, *p-*cymene ArC–H), 4.99 (br. s, 1H, Cp −CC_4_H_4_), 4.89 (br. s, 1H, Cp −CC_4_H_4_), 4.47 (br. d, 1H, ^3^*J*(^1^H–^1^H) = 0.9 Hz, Cp −CC_4_H_4_), 4.43 (br. d, 1H, ^3^*J*(^1^H–^1^H) = 0.9 Hz, Cp CC_4_H_4_), 4.27 (s, 5H, Cp −C_5_H_5_), 3.89 (s, 3H, *meta* Ar–C(OCH_3_)), 3.07 (sept, 1H, ^3^*J*(^1^H–^1^H) = 6.7 Hz, *p-*cymene −CCH(CH_3_)_2_, H_8_), 2.33 (s, 3H, *p-*cymene −C(CH_3_)), 1.50 (dd, 6H, ^3^*J*(^1^H–^1^H) = 6.9 Hz, ^4^*J*(^1^H–^1^H) = 1.6 Hz, *p-*cymene −CCH(CH_3_)_2_). ^13^C{^1^H} (125 MHz, (CD_3_)_2_CO, δ); 186.9 (Q C–O), 178.1 (Q C–O), 160.6 (Q *meta* Ar–C(OCH_3_)), 142.0 (Q Ar–C), 129.9 (*ortho* Ar–CH), 120.1 (*ortho* Ar–CH), 116.9 (*para* Ar–CH), 113.3 (*meta* Ar–CH), 99.3 (Q *p*-cymene −CCH(CH_3_)_2_), 97.9 (Q *p*-cymene −C(CH_2_)), 93.5 (methine −COCHCO−), 84.7 (*p*-cymene Ar–CH), 84.7 (*p*-cymene Ar–CH), 82.1 (Q Cp −CC_4_H_4_), 79.9 (*p*-cymene Ar–CH), 79.9 (*p*-cymene Ar–CH), 71.7 (Cp −CC_4_H_4_), 71.7 (Cp −CC_4_H_4_), 70.9 (Cp −C_5_H_5_), 69.9 (Cp CC_4_H_4_), 68.8 (Cp −CC_4_H_4_), 55.6 (*meta* Ar–C(O*C*H_3_)), 31.7 (*p*-cymene −CCH(CH_3_)_2_), 22.8 (*p*-cymene −CCH(CH_3_)_2_), 22.6 (*p*-cymene −CCH(CH_3_)_2_), 17.8 (*p*-cymene −C(CH_3_)). Analysis calculated for C_30_H_31_ClFeO_3_Ru: C 57.02, H 4.94, Cl 5.61%. Found: C 57.09, H 4.90, Cl 5.53%. HR-MS [ES^+^] calculated for C_30_H_31_ClFeO_3_Ru: 632.036. Found: 597.068 [MH^+^]-Cl.

#### Complex **12**

Yield: 0.22 g, 89%. ^1^H NMR (500 MHz, (CD_3_)_2_CO, δ); 7.83 (dt, 2H, ^3^*J*(^1^H–^1^H) = 9.2 Hz, ^4^*J*(^1^H–^1^H) = 2.8 Hz, *ortho* ArC–H), 6.82 (dt, 2H, ^3^*J*(^1^H–^1^H) = 9.2 Hz, ^4^*J*(^1^H–^1^H) = 2.8 Hz, *meta* ArC–H), 6.01 (s, 1H, −COCHCO−), 5.53 (dd, 2H, ^3^*J*(^1^H–^1^H) = 6.0 Hz, ^4^*J*(^1^H–^1^H) = 0.9 Hz, *p-*cymene ArC–H), 5.21 (d, 2H, ^3^*J*(^1^H–^1^H) = 6.4 Hz, *p-*cymene ArC–H), 4.82 (t, 1H, ^3^*J*(^1^H–^1^H) = 1.2 Hz, Cp −CC_4_H_4_), 4.71 (t, 1H, ^3^*J*(^1^H–^1^H) = 1.2 Hz, Cp −CC_4_H_4_), 4.92 (m, 1H, Cp −CC_4_H_4_), 4.25 (m, 1H, Cp −CC_4_H_4_), 4.10 (s, 5H, Cp −C_5_H_5_), 3.73 (s, 3H, *para* Ar–C(OCH_3_)), 2.91 (sept, 1H, ^3^*J*(^1^H–^1^H) = 6.9 Hz, *p-*cymene −CCH(CH_3_)_2_), 2.16 (s, 3H, *p-*cymene −C(CH_3_)), 1.34 (d, 6H, ^3^*J*(^1^H–^1^H) = 7.3 Hz, *p-*cymene −CCH(CH_3_)_2_). ^13^C{^1^H} NMR (125 MHz, (CD_3_)_2_CO, δ); 185.9 (Q C–O), 178.0 (Q C–O), 162.7 (Q *para* Ar–C(OCH_3_)), 132.8 (Q Ar–C), 129.7 (*ortho* Ar–CH), 114.2 (*meta* Ar–CH), 99.3 (Q *p*-cymene −CCH(CH_3_)_2_), 97.8 (Q *p*-cymene −C(CH_3_)), 92.6 (methine −COCHCO−), 84.6 (*p*-cymene Ar–CH), 84.5 (*p*-cymene Ar–CH), 82.4 (Q Cp −CC_4_H_4_), 79.9 (*p*-cymene Ar–CH), 79.9 (*p*-cymene Ar–CH), 71.4 (Cp −CC_4_H_4_), 71.4 (Cp −CC_4_H_4_), 70.9 (Cp −C_5_H_5_), 69.9 (Cp −CC_4_H_4_), 68.6 (Cp −CC_4_H_4_), 55.8 (*para* Ar–C(OCH_3_)), 31.7 (*p*-cymene −CCH(CH_3_)_2_), 22.7 (*p*-cymene −CCH(CH_3_)), 22.6 (*p*-cymene −CCH(CH_3_)), 17.8 (*p*-cymene −C(CH_3_)). Analysis calculated for C_30_H_31_ClFeO_3_Ru: C 57.02, H 4.94, Cl 5.61%. Found: C 57.00, H 5.00, Cl 5.50%. HR-MS [ES^+^] calculated for C_30_H_31_ClFeO_3_Ru: 632.036. Found: 597.066 [MH^+^]-Cl.

#### Complex **13**

Yield: 0.19 g, 75%. ^1^H NMR (500 MHz, (CD_3_)_2_CO, δ); 7.82 (d, 2H, ^3^*J*(^1^H–^1^H) = 8.9 Hz, *ortho* ArC–H), 6.80 (d, 2H, ^3^*J*(^1^H–^1^H) = 8.9 Hz, *meta* ArC–H), 6.00 (s, 1H, methine −COCHCO−), 5.53 (dt, 2H, ^3^*J*(^1^H–^1^H) = 4.8 Hz, ^4^*J*(^1^H–^1^H) = 1.2 Hz, *p-*cymene ArC–H), 5.21 (d, 2H, ^3^*J*(^1^H–^1^H) = 5.2 Hz, *p-*cymene ArC–H), 4.82 (t, 1H, ^3^*J*(^1^H–^1^H) = 1.2 Hz, Cp −CC_4_H_4_), 4.70 (t, 1H, ^3^*J*(^1^H–^1^H) = 1.2 Hz, Cp −CC_4_H_4_), 4.29 (m, 1H, Cp −CC_4_H_4_), 4.25 (m, 1H, Cp −CC_4_H_4_), 4.10 (s, 5H, Cp −C_5_H_5_), 3.99 (q, 2H, ^3^*J*(^1^H–^1^H) = 6.9 Hz, *para* ArC(OCH_2_CH_3_)), 2.90 (sept, 1H, ^3^*J*(^1^H–^1^H) = 6.9 Hz, −CCH(CH_3_)_2_), 2.16 (s, 3H, *p-*cymene −C(CH_3_)), 1.33 (d, 6H, ^3^*J*(^1^H–^1^H) = 6.9 Hz, *p-*cymene −CCH(CH_3_)_2_) 1.26 (t, 3H, ^3^*J*(^1^H–^1^H) = 6.9 Hz, *para* ArC(OCH_2_CH_3_)). ^13^C{^1^H} NMR (125 MHz, (CD_3_)_2_CO, δ); 185.8 (Q C–O), 178.0 (Q C–O), 162.1 (Q *para* Ar–C(OCH_2_CH_3_)), 132.6 (Q Ar–C), 129.7 (*ortho* Ar–CH), 114.7 (*meta* Ar–CH), 99.3 (Q *p*-cymene −CCH(CH_3_)_2_), 97.7 (Q *p*-cymene −C(CH_3_)), 92.5 (methine −COCHCO−), 84.6 (*p*-cymene Ar–CH), 84.5 (*p*-cymene Ar–CH_6_), 82.4 (Q Cp −CC_4_H_4_), 79.9 (*p*-cymene Ar–CH), 79.9 (*p*-cymene Ar–CH), 71.4 (Cp −CC_4_H_4_), 71.4 (Cp −CC_4_H_4_), 70.9 (Cp −C_5_H_5_), 69.8 (Cp −CC_4_H_4_), 68.6 (Cp −CC_4_H_4_), 64.2 (*para* ArC(OCH_2_CH_3_)), 31.7 (*p*-cymene −CCH(CH_3_)_2_), 22.8 (*p*-cymene −CCH(CH_3_)_2_), 22.6 (*p*-cymene −CCH(CH_3_)_2_), 17.9 (*p*-cymene −C(CH_3_)), 15.0 (*para* ArC(OCH_2_CH_3_). Analysis calculated for C_31_H_33_ClFeO_3_Ru: C 57.64, H 5.15, Cl 5.49%. Found: C 57.65, H 5.25, Cl 5.40%. HR-MS [ES^+^] calculated for C_31_H_33_ClFeO_3_Ru: 646.051. Found: 611.085 [MH^+^]-Cl.

#### Complex **14**

Yield: 0.18 g, 72%. ^1^H NMR (500 MHz, (CD_3_)_2_CO, δ); 7.87 (td, 1H, ^3^*J*(^1^H–^1^H) = 7.7 Hz, ^4^*J*(^1^H–^19^F) = 1.2 Hz, *ortho* ArC–H), 7.49 (m, 1H, *meta* ArC–H), 7.28 (t, 1H, ^3^*J*(^1^H–^1^H) = 7.6 Hz, *meta* ArC–H), 7.19 (dd, 1H, ^3^*J*(^1^H–^1^H) = 11.8 Hz, ^4^*J*(^1^H–^19^F) = 1.2 Hz, *para* ArC–H), 6.02 (s, 1H, −COCHCO−), 5.71 (d, 2H, ^3^*J*(^1^H–^1^H) = 4.8 Hz, *p*-cymene ArC–H), 5.39 (t, 2H, ^3^*J*(^1^H–^1^H) = 4.8 Hz, *p*-cymene ArC–H), 4.93 (d, 1H, ^3^*J*(^1^H–^1^H) = 0.8 Hz, Cp −CC_4_H_4_), 4.75 (d, 1H, ^3^*J*(^1^H–^1^H) = 0.9 Hz, Cp −CC_4_H_4_), 4.49 (br. t, 1H, Cp −CC_4_H_4_), 4.45 (br. t, 1H, Cp −CC_4_H_4_), 4.27 (s, 5H, Cp −C_5_H_5_), 3.04 (sept, 1H, ^3^*J*(^1^H–^1^H) = 6.9 Hz, *p-*cymene −CCH(CH_3_)_2_), 1.99 (s, 3H, *p-*cymene −C(CH_3_)), 1.48 (d, 6H, ^3^*J*(^1^H–^1^H) = 6.9 Hz, *p-*cymene −CCH(CH_3_)_2_). ^13^C{^1^H} (125 MHz, (CD_3_)_2_CO, δ); 187.1 (Q C–O), 174.8 (d, Q C–O, ^4^*J*(^13^C–^19^F) = 3.6 Hz), 160.6 (d, *ortho* Ar–CF, ^1^*J*(^13^C–^19^F) = 250.1 Hz), 132.3 (d, *ortho* Ar–CH, ^3^*J*(^13^C–^19^F) = 8.8 Hz), 131.7 (d, *meta* Ar–CH, ^4^*J*(^13^C–^19^F) = 3.1 Hz), 129.3 (d, Q Ar–C, ^2^*J*(^13^C–^19^F) = 11.9 Hz), 125.1 (d, *para* Ar–CH, ^3^*J*(^13^C–^19^F) = 3.6 Hz), 116.9 (d, *meta* Ar–CH, ^2^*J*(^13^C–^19^F) = 23.9 Hz), 99.5 (Q *p*-cymene −CCH(CH_3_)_2_), 97.9 (d, methine −COCHCO–, ^4^*J*(^13^C–^19^F) = 5.2 Hz), 97.8 (Q *p*-cymene −C(CH_3_)), 84.5 (*p*-cymene Ar–CH), 84.4 (*p*-cymene Ar–CH), 81.7 (Q Cp −CC_4_H_4_), 79.9 (*p*-cymene Ar–CH), 71.9 (Cp −CC_4_H_4_), 71.9 (Cp −CC_4_H_4_), 71.1 (Cp −CC_5_H_5_), 69.9 (Cp −CC_4_H_4_), 68.8 (Cp −CC_4_H_4_), 31.7 (*p*cymene −CCH(CH_3_)_2_), 22.7 (*p*-cymene −CCH(CH_3_)_2_), 22.6 (*p*-cymene −CCH(CH_3_)_2_), 17.9 (*p*-cymene −C(CH_3_)). Analysis calculated for C_29_H_28_ClFFeO_2_Ru: C 56.19, H 4.55, Cl 5.72%. Found: C 55.90, H 4.70, Cl 5.90%. HR-MS [ES^+^] calculated for C_29_H_28_ClFFeO_2_Ru: 620.016. Found: 585.049 [MH^+^]-Cl.

#### Complex **15**

Yield: 0.19 g, 78%. ^1^H NMR (500 MHz, (CD_3_)_2_CO, δ); 7.70 (br. dt, 1H, ^3^*J*(^1^H–^1^H) = 7.8 Hz, *ortho* ArC–H), 7.61 (dq, 1H, ^3^*J*(^1^H–^1^H) = 10.4 Hz, ^4^*J*(^1^H–^1^H) = 1.6, 0.9 Hz, *ortho* ArC–H), 7.36 (m, 1H, *para* ArC–H), 7.14 (td, 1H, ^3^*J*(^1^H–^1^H) = 8.3 Hz, ^4^*J*(^1^H–^1^H) = 2.3 Hz, *meta* ArC–H), 6.08 (s, 1H, methine −COCHCO−), 5.62 (d, 2H, ^3^*J*(^1^H–^1^H) = 6.2 Hz, *p-*cymene ArC–H), 5.29 (t, 2H, ^3^*J*(^1^H–^1^H) = 4.7 Hz, *p-*cymene ArC–H), 4.89 (quintet, 1H, ^3^*J*(^1^H–^1^H) = 1.2 Hz, Cp −CC_4_H_4_), 4.81 (quintet, 1H, ^3^*J*(^1^H–^1^H) = 1.2 Hz, Cp −CC_4_H_4_), 4.37 (m, 1H, Cp −CC_4_H_4_), 4.34 (m, 1H, Cp −CC_4_H_4_), 4.15 (s, 5H, Cp −C_5_H_5_), 2.94 (sept, 1H, ^3^*J*(^1^H–^1^H) = 6.9 Hz, *p-*cymene -CCH(CH_3_)_2_), 2.20 (s, 3H, *p-*cymene −C(CH_3_)), 1.38 (d, 6H, ^3^*J*(^1^H–^1^H) = 7.1 Hz, *p-*cymene −CCH(CH_3_)_2_). ^13^C{^1^H} NMR (125 MHz, (CD_3_)_2_CO, δ); 187.6 (Q C–O), 179.1 (Q C–O), 164.6 (d, Q *meta* Ar–CF, ^1^*J*(^13^C–^19^F) = 243.9 Hz), 143.0 (d, Q Ar–C, ^3^*J*(^13^C–^19^F) = 6.7 Hz), 130.8 (d, *meta* Ar–CH, ^3^*J*(^13^C–^19^F) = 8.3 Hz), 123.6 (d, *ortho* Ar–CH, ^4^*J*(^13^C–^19^F) = 2.6 Hz), 117.7 (d, *ortho* Ar–CH, ^2^*J*(^13^C–^19^F) = 21.3 Hz), 114.5 (d, *para* Ar–CH, ^2^*J*(^13^C–^19^F) = 22.8 Hz), 99.5 (Q *p*-cymene −CCH(CH_3_)_2_), 97.9 (Q *p*-cymene −C(CH_2_)), 93.6 (methine −COCHCO−), 84.6 (*p*-cymene Ar–CH), 84.5 (*p*-cymene Ar–CH), 81.8 (Q Cp −CC_4_H_5_), 80.0 (*p*-cymene Ar–CH), 80.0 (*p*-cymene Ar–CH), 71.9 (Cp −CC_4_H_4_), 71.8 (Cp −CC_4_H_4_), 71.0 (Cp −C_5_H_5_), 70.0 (Cp −CC_4_H_4_), 68.9 (Cp −CC_4_H_4_), 31.7 (*p*-cymene −CCH(CH_3_)_2_), 22.7 (*p*-cymene CCH(CH_3_)_2_), 22.6 (*p*-cymene −CCH(CH_3_)_2_), 17.9 (*p*-cymene −C(CH_3_)). Analysis calculated for C_29_H_28_ClFFeO_2_Ru:: C 56.19, H 4.55, Cl 5.72%. Found: C 56.30, H 4.85, Cl 5.30%. HR-MS [ES^+^] calculated for C_29_H_28_ClFFeO_2_Ru: 620.016. Found: 585.048 [MH^+^]-Cl.

#### Complex **16**

Yield: 0.19 g, 76%. ^1^H NMR (500 MHz, (CD_3_)_2_CO, δ); 7.45 (d, 2H, ^3^*J*(^1^H–^1^H) = 6.9 Hz, *ortho* ArC–H), 6.99 (tt, 1H, ^3^*J*(^1^H–^1^H) = 8.9 Hz, ^4^*J*(^1^H–^1^H) = 2.1 Hz, *para* ArC–H), 6.05 (s, 1H, methine −COCHCO−), 5.60 (d, 2H, ^3^*J*(^1^H–^1^H) = 6.0 Hz, *p-*cymene ArC–H), 5.28 (t, 2H, ^3^*J*(^1^H–^1^H) = 5.0 Hz, *p-*cymene ArC–H), 4.87 (br. s, 1H, Cp −CC_4_H_4_), 4.83 (br. s, 1H, Cp −CC_4_H_4_), 4.36 (br. s, 1H, Cp −CC_4_H_4_), 4.32 (br. s, 1H, Cp −CC_4_H_4_), 4.12 (s, 5H, Cp −C_5_H_5_), 2.90 (sept, 1H, ^3^*J*(^1^H–^1^H) = 7.0 Hz, *p-*cymene −CCH(CH_3_)_2_), 2.17 (s, 3H, −C(CH_3_)), 1.34 (d, 6H, ^3^*J*(^1^H–^1^H) = 6.9 Hz, *p-*cymene −CCH(CH_3_)_2_). ^13^C{^1^H} NMR (125 MHz, (CD_3_)_2_CO, δ); 188.4 (Q C–O), 174.6 (Q C–O), 163.7 (dd, *meta* Ar–CF, ^1^*J*(^13^C–^19^F) = 264.0 Hz, ^3^*J*(^13^C–^19^F) = 12.8 Hz), 144.3 (Q Ar–C, ^3^*J*(^13^C–^19^F) = 8.3 Hz), 110.7 (dd, *ortho* Ar–CH, ^2^*J*(^13^C–^19^F) = 20.2, 6.2 Hz), 105.9 (t, *para* Ar–CH, ^2^*J*(^13^C–^19^F) = 26.0 Hz), 99.6 (Q *p-*cymene, −CCH(CH_3_)_2_), 98.0 (Q *p-*cymene −C(CH_3_)), 93.7 (methine −COCHCO−), 84.6 (*p-*cymene Ar–CH), 84.5 (*p-*cymene Ar–CH), 81.6 (Q Cp −CC_4_H_4_), 80.0 (*p-*cymene Ar–CH), 72.1 (Cp −CC_4_H_4_), 72.0 (Cp −CC_4_H_4_), 71.0 (Cp −C_5_H_5_), 70.0 (Cp −CC_4_H_4_), 69.1 (Cp −CC_4_H_4_), 31.7 (*p*-cymene −CCH(CH_3_)_2_), 22.7 (*p*-cymene −CCH(CH_3_)_2_), 22.6 (*p*-cymene −CCH(CH_3_)_2_), 17.9 (*p*-cymene −C(CH_3_)). Analysis calculated for C_29_H_27_ClF_2_FeO_2_Ru: C 54.60, H 4.27, Cl 5.56%. Found: C 55.20, H 4.40, Cl 5.57%. HR-MS [ES^+^] calculated for C_29_H_27_ClF_2_FeO_2_Ru: 638.006. Found: 603.040 [MH^+^]-Cl.

#### Complex **17**

Yield: 0.12 g, 82%. ^1^H NMR (500 MHz, (CD_3_)_2_CO, δ); 7.91 (q, 2H, ^3^*J*(^1^H–^1^H) = 5.5, ^3^*J*(^1^H–^19^F) = 3.2 Hz, *meta* ArC–H), 7.04 (t, 2H, ^3^*J*(^1^H–^1^H) = 8.7 Hz, *meta* ArC–H), 6.02 (s, 1H, methine −COCHCO−), 5.56 (d, 2H, ^3^*J*(^1^H–^1^H) = 6.4 Hz, *p-*cymene ArC–H), 5.24 (t, 2H, ^3^*J*(^1^H–^1^H) = 4.4 Hz, *p-*cymene ArC–H), 4.83 (t, 1H, ^3^*J*(^1^H–^1^H) = 1.2 Hz, Cp −CC_4_H_4_), 4.73 (t, 1H, ^3^*J*(^1^H–^1^H) = 1.2 Hz, Cp −CC_4_H_4_), 4.32 (br. q, 1H, ^3^*J*(^1^H–^1^H) = 2.4, 1.1 Hz, Cp −CC_4_H_4_), 4.28 (br. q, 1H, ^3^*J*(^1^H–^1^H) = 2.4, 1.1 Hz, Cp −CC_4_H_4_), 4.11 (s, 5H, Cp −C_5_H_5_), 2.90 (sept, 1H, ^3^*J*(^1^H–^1^H) = 6.9 Hz, *p-*cymene −CCH(CH_3_)_2_, H_8_), 2.16 (s, 3H, *p-*cymene −C(CH_3_)), 1.33 (d, 6H, ^3^*J*(^1^H–^1^H) = 6.9 Hz, *p-*cymene −CCH(CH_3_)_2_); ^13^C{^1^H} NMR (125 MHz, (CD_3_)_2_CO, δ); 187.0 (Q C–O), 177.0 (Q C–O), 165.0 (d, *para* Ar–CF, ^1^*J*(^13^C–^19^F) = 248.6 Hz), 136.9 (d, Q Ar-C, ^4^*J*(^13^C–^19^F) = 3.1 Hz), 130.3 (d, *ortho* Ar-CH, ^3^*J*(^13^C–^19^F) = 8.8 Hz), 115.6 (d, *meta* Ar-CH, ^2^*J*(^13^C–^19^F) = 21.8 Hz), 99.5 (Q *p*-cymene −CCH(CH_3_)_2_), 97.8 (Q *p*-cymene −C(CH_3_)), 93.2 (methine −COCHCO−), 84.6 (*p*-cymene Ar–CH), 84.5 (*p*-cymene Ar–CH), 82.0 (Q Cp −CC_4_H_4_), 80.0 (*p*-cymene Ar–CH), 71.7 (Cp −CC_4_H_4_), 71.7 (Cp −CC_4_H_4_), 70.9 (Cp −C_5_H_5_), 69.9 (Cp −CC_4_H_4_), 68.8 (Cp −CC_4_H_4_), 31.7 (*p*-cymene −CCH(CH_3_)_2_), 22.7 (*p*-cymene −CCH(CH_3_)_2_), 22.6 (*p*-cymene −CCH(CH_3_)_2_), 17.9 (*p*-cymene −C(CH_3_)). Analysis calculated for C_29_H_28_ClFFeO_2_Ru: C 56.19, H 4.55, Cl 5.72%. Found: C 56.20, H 4.95, Cl 5.95%. HR-MS [ES^+^] calculated for C_29_H_28_ClFFeO_2_Ru: 620.016. Found: 585.047 [MH^+^]-Cl.

#### Complex **18**

Yield: 0.23 g, 92%. ^1^H NMR (500 MHz, (CD_3_)_2_CO, δ); 7.99 (t, 1H, ^3^*J*(^1^H–^1^H) = 1.8 Hz, *ortho* ArC–H), 7.93 (dt, 1H, ^3^*J*(^1^H–^1^H) = 7.8 Hz, ^4^*J*(^1^H–^1^H) = 1.4 Hz, *ortho* ArC–H), 7.52 (dq, 1H, ^3^*J*(^1^H–^1^H) = 8.0 Hz, ^4^*J*(^1^H–^1^H) = 1.1 Hz, *para* ArC–H), 7.46 (t, 1H, ^3^*J*(^1^H–^1^H) = 7.8 Hz, *meta* ArC–H), 6.20 (s, 1H, methine −COCHCO−), 5.73 (dd, 2H, ^3^*J*(^1^H–^1^H) = 4.8 Hz, ^4^*J*(^1^H–^1^H) = 1.4 Hz, *p-*cymene ArC-H), 5.42 (t, 2H, ^3^*J*(^1^H–^1^H) = 5.0 Hz, *p-*cymene ArC–H), 5.01 (quin, 1H, ^3^*J*(^1^H–^1^H) = 1.3 Hz, Cp −CC_4_H_4_), 4.93 (pent, 1H, ^3^*J*(^1^H–^1^H) = 1.3 Hz, Cp −CC_4_H_4_), 4.49 (m, 1H, Cp −CC_4_H_4_), 4.45 (m, 1H, Cp −CC_4_H_4_), 4.27 (s, 5H, Cp −C_5_H_5_), 3.06 (sept, 1H, ^3^*J*(^1^H–^1^H) = 6.9 Hz, *p-*cymene −CCH(CH_3_)_2_), 2.32 (s, 3H, *p-*cymene −C(CH_3_)), 1.50 (d, 6H, ^3^*J*(^1^H–^1^H) = 7.1 Hz, *p-*cymene −CCH(CH_3_)_2_); ^13^C{^1^H} NMR (125 MHz, (CD_3_)_2_CO, δ); 187.7 (Q C–O), 176.3 (Q C–O), 142.6 (*meta* Ar–CCl), 134.6 (Q Ar–C), 130.9 (*ortho* Ar–CH), 130.7 (*ortho* Ar–CH), 127.8 (*para* Ar–CH), 126.2 (*meta* Ar–CH), 99.5 (Q *p*-cymene −CCH(CH_3_)_2_), 97.6 (Q *p*-cymene −C(CH_3_)), 93.6 (methine −COCHCO−), 84.6 (*p*-cymene Ar–CH), 84.5 (*p*-cymene Ar–CH), 81.8 (Q Cp −CC_4_H_4_), 80.0 (*p*-cymene Ar–CH), 80.0 (*p*-cymene Ar–CH), 71.9 (Cp −CC_4_H_4_), 71.9 (Cp −CC_4_H_4_), 71.0 (Cp −C_5_H_5_), 70.0 (Cp −CC_4_H_4_), 68.9 (Cp −CC_4_H_4_), 31.7 (*p*-cymene −CCH(CH_3_)_2_), 22.7 (*p*-cymene −CCH(CH_3_)_2_), 22.6 (*p*-cymene −CCH(CH_3_)_2_), 17.9 (*p*-cymene −C(CH_3_)). Analysis calculated for C_29_H_28_Cl_2_FeO_2_Ru: C 54.74, H 4.44, Cl 11.14%. Found: C 53.95, H 4.51, Cl 11.06%. HR-MS [ES^+^] calculated for C_29_H_28_Cl_2_FeO_2_Ru: 635.986. Found: 601.018 [MH^+^]-Cl.

#### Complex **19**

Yield: 0.16 g, 61%. ^1^H NMR (500 MHz, (CD_3_)_2_CO, δ); 7.94 (d, 2H, ^3^*J*(^1^H–^1^H) = 1.8 Hz, *ortho* ArC–H), 7.59 (t, 1H, ^4^*J*(^1^H–^1^H) = 1.8 Hz, *para* ArC–H), 6.21 (s, 1H, methine −COCHCO−), 5.75 (d, 2H, ^3^*J*(^1^H–^1^H) = 5.7 Hz, *p-*cymene ArC–H), 5.44 (t, 2H, ^3^*J*(^1^H–^1^H) = 5.7 Hz, *p-*cymene ArC–H), 5.03 (d, 1H, ^3^*J*(^1^H–^1^H) = 0.9 Hz, Cp −CC_4_H_4_), 4.98 (d, 1H, ^3^*J*(^1^H–^1^H) = 1.1 Hz, Cp −CC_4_H_4_), 4.52 (br. t, 1H, Cp −CC_4_H_4_), 4.47 (br. t, 1H, Cp −CC_4_H_4_), 4.27 (s, 5H, Cp −C_5_H_5_), 3.05 (sept, 1H, *J* 6.9 Hz, *p-*cymene −CCH(CH_3_)_2_), 2.32 (s, 3H, *p-*cymene −C(CH_3_)), 1.49 (d, 6H, ^3^*J*(^1^H–^1^H) = 7.1 Hz, *p-*cymene −CCH(CH_3_)_2_); ^13^C{^1^H} NMR (125 MHz, (CD_3_)_2_CO, δ); 187.7 (Q C–O), 17.5 (Q C–O), 143.0 (Q *meta* Ar–CCl), 134.6 (Q Ar–C), 129.4 (*ortho* Ar–CH), 125.5 (*para* Ar–CH), 98.7 (Q *p*-cymene −CCH(CH_3_)_2_), 97.0 (Q *p*-cymene −C(CH)_3_), 92.9 (methine −COCHCO−), 83.7 (*p*-cymene Ar–CH), 83.6 (*p*-cymene Ar–CH), 80.6 (Q Cp −CC_4_H_4_), 79.2 (*p*-cymene Ar–CH), 79.1 (*p*-cymene Ar–CH), 71.3 (Cp −CC_4_H_4_), 71.2 (Cp −CC_4_H_4_), 70.1 (Cp −C_5_H_5_), 69.2 (Cp −CC_4_H_4_), 68.2 (Cp −CC_4_H_4_), 30.9 (*p*-cymene −CCH(CH_3_)_2_), 21.8 (*p*-cymene −CCH(CH_3_)_2_), 21.7 (*p*-cymene −CCH(CH_3_)_2_), 17.0 (*p*-cymene −C(CH_3_)). Analysis calculated for C_29_H_27_Cl_3_FeO_2_Ru·H_2_O: C 50.57, H 4.24, Cl 15.44%. Found: C 51.00, H 3.90, Cl 15.89%. HR-MS [ES+] calculated for C_29_H_27_Cl_3_FeO_2_Ru: 669.947. Found: 634.979 [M^+^]-Cl.

#### Complex **20**

Yield: 0.23 g, 91%. ^1^H NMR (500 MHz, (CD_3_)_2_CO, δ); 7.86 (br. d, 2H, ^3^*J*(^1^H–^1^H) = 8.7 Hz, *ortho* ArC–H), 7.31 (br. d, 2H, ^3^*J*(^1^H–^1^H) = 8.7 Hz, *meta* ArC–H), 6.03 (s, 1H, methine −COCHCO−), 5.56 (d, 2H, ^3^*J*(^1^H–^1^H) = 6.0 Hz, *p-*cymene ArC–H), 5.24 (t, 2H, ^3^*J*(^1^H–^1^H) = 4.6 Hz, *p-*cymene ArC–H), 4.84 (t, 1H, ^3^*J*(^1^H–^1^H) = 1.4 Hz, Cp −CC_4_H_4_), 4.74 (t, 1H, ^3^*J*(^1^H–^1^H) = 1.2 Hz, Cp −CC_4_H_4_), 4.33 (q, 1H, ^3^*J*(^1^H–^1^H) = 2.3, 1.2 Hz, Cp −CC_4_H_4_), 4.29 (q, 1H, ^3^*J*(^1^H–^1^H) = 2.3, 1.4 Hz, Cp −CC_4_H_4_), 4.11 (s, 5H, Cp −C_5_H_5_), 2.90 (sept, 1H, ^3^*J*(^1^H–^1^H) = 6.9 Hz, −CCH(CH_3_)_2_), 2.16 (s, 3H, *p-*cymene −C(CH_3_)), 1.33 (d, 6H, ^3^*J*(^1^H–^1^H) = 6.9 Hz, *p-*cymene −CCH(CH_3_)_2_); ^13^C{^1^H} NMR (125 MHz, (CD_3_)_2_CO, δ); 187.3 (Q C–O), 176.8 (Q C–O), 139.2 (Q Ar–C), 136.6 (Q *para* Ar–C), 129.6 (*ortho* Ar–CH), 129.0 (*meta* Ar–CH), 99.5 (Q *p*-cymene −CCH(CH_3_)_2_), 97.8 (Q *p*-cymene −C(CH_3_)), 93.4 (methine −COCHCO−), 84.6 (*p*-cymene Ar–CH), 84.5 (*p*-cymene Ar–CH), 81.9 (Q Cp −CC_4_H_4_), 80.0 (*p*-cymene Ar–CH), 71.9 (Cp −CC_4_H_4_), 71.8 (Cp −CC_4_H_4_), 71.0 (Cp −C_5_H_5_), 70.0 (Cp −CC_4_H_4_), 68.8 (Cp −CC_4_H_4_), 31.74 (*p*-cymene −CCH(CH_3_)_2_), 22.7 (*p*-cymene −CCH(CH_3_)_2_), 22.6 (*p*-cymene −CCH(CH_3_)_2_), 17.9 (*p*-cymene −C(CH_3_)). Analysis calculated for C_29_H_28_Cl_2_FeO_2_Ru: C 54.74, H 4.44, Cl 11.14%. Found: C 54.90, H 4.50, Cl 11.10%. HR-MS [ES^+^] calculated for C_29_H_28_Cl_2_FeO_2_Ru: 635.986. Found: 601.016 [MH^+^]-Cl.

#### Complex **21**

Yield: 0.23 g, 84%. ^1^H NMR (500 MHz, (CD_3_)_2_CO, δ); 7.99 (t, 1H, ^3^*J*(^1^H–^1^H) = 1.7 Hz, *ortho* ArC–H), 7.81 (dt, 1H, ^3^*J*(^1^H–^1^H) = 7.8 Hz, ^4^*J*(^1^H–^1^H) = 1.1 Hz, *ortho* ArC–H), 7.51 (dq, 1H, ^3^*J*(^1^H–^1^H) = 7.8 Hz, ^4^*J*(^1^H–^1^H) = 0.9 Hz, *para* ArC–H), 7.24 (t, 1H, ^3^*J*(^1^H–^1^H) = 7.8 Hz, *meta* ArC–H), 6.03 (s, 1H, methine −COCHCO−), 5.57 (dd, 2H, ^3^*J*(^1^H–^1^H) = 5.0 Hz, ^4^*J*(^1^H–^1^H) = 1.4 Hz, *p-*cymene ArC–H), 5.26 (t, 2H, ^3^*J*(^1^H–^1^H) = 5.0 Hz, *p-*cymene ArC–H), 4.85 (quin, 1H, ^3^*J*(^1^H–^1^H) = 1.2 Hz, Cp −CC_4_H_4_), 4.77 (quin, 1H, ^3^*J*(^1^H–^1^H) = 1.2 Hz, Cp −CC_4_H_4_), 4.34 (m, 1H, Cp −CC_4_H_4_), 4.30 (m, 1H, Cp −CC_4_H_4_), 4.11 (s, 5H, Cp −C_5_H_5_), 2.90 (sept, 1H, ^3^*J*(^1^H–^1^H) = 6.9 Hz, *p-*cymene −CCH(CH_3_)_2_), 2.16 (s, 3H, *p-*cymene −C(CH_3_)), 1.33 (d, 6H, ^3^*J*(^1^H–^1^H) = 7.1 Hz, *p-*cymene −CCH(CH_3_)_2_); ^13^C{^1^H} (125 MHz, (CD_3_)_2_CO, δ); 187.7 (Q C–O), 176.2 (Q C–O), 142.8 (Q *meta* Ar–C), 133.8 (*ortho* Ar–CH), 130.9 (*ortho* Ar–CH), 130.8 (*meta* Ar–CH), 126.6 (*para* Ar–CH), 122.8 (*meta* Ar–CBr), 99.5 (Q *p*-cymene −CCH(CH3)_2_), 97.9 (Q *p*-cymene −C(CH_3_)), 93.6 (methine −COCHCO−), 84.6 (*p*-cymene Ar–CH), 84.5 (*p*-cymene Ar–CH), 81.7 (Q Cp −CC_4_H_4_), 80.1 (*p*-cymene Ar–CH), 80.0 (*p*-cymene Ar–CH), 72.0 (Cp −CC_4_H_4_), 71.9 (Cp −CC_4_H_4_), 71.0 (Cp −C_5_H_5_), 70.0 (Cp −CC_4_H_4_), 68.9 (Cp −CC_4_H_4_), 31.7 (*p*-cymene −CCH(CH_3_)_2_), 22.7 (*p*-cymene −CCH(CH_3_)_2_), 22.6 (*p*-cymene −CCH(CH_3_)_2_), 17.9 (*p*-cymene −C(CH_3_)). Analysis calculated for C_29_H_28_BrClFeO_2_Ru: C 51.16, H 4.15%. Found: C 51.06, H 4.18%. HR-MS [ES^+^] calculated for C_29_H_28_BrClFeO_2_Ru: 679.935. Found: 646.967 [MH^+^]-Cl.

#### Complex **22**

Yield: 0.17 g, 64%. ^1^H NMR (500 MHz, (CD_3_)_2_CO, δ); 7.78 (d, 2H, ^3^*J*(^1^H–^1^H) = 8.7 Hz, *ortho* ArC–H), 7.46 (d, 2H, ^3^*J*(^1^H–^1^H) = 8.7 Hz, *meta* ArC–H), 6.03 (s, 1H, methine −COCHCO−), 5.56 (d, 2H, ^3^*J*(^1^H–^1^H) = 6.2 Hz, *p-*cymene ArC–H), 5.24 (t, 2H, ^3^*J*(^1^H–^1^H) = 4.7 Hz, *p-*cymene ArC–H), 4.83 (q, 1H, ^3^*J*(^1^H–^1^H) = 1.3 Hz, Cp −CC_4_H_4_), 7.47 (q, 1H, ^3^*J*(^1^H–^1^H) = 1.3 Hz, Cp −CC_4_H_4_), 4.33 (m, 1H, Cp −CC_4_H_4_), 4.29 (m, 1H, Cp −CC_4_H_4_), 4.11 (s, 5H, Cp −C_5_H_5_), 2.90 (sept, 1H, ^3^*J*(^1^H–^1^H) = 6.9 Hz, *p-*cymene −CCH(CH_3_)_2_), 2.16 (s, 3H, *p-*cymene −C(CH_3_)), 1.33 (d, 6H, ^3^*J*(^1^H–^1^H) = 6.9 Hz, *p-*cymene −CCH(CH_3_)_2_). ^13^C{^1^H} NMR (125 MHz, (CD_3_)_2_CO, δ); 194.4 (Q C–O), 189.7 (Q C–O), 139.7 (Q Ar–C), 132.0 (*ortho* Ar–CH), 129.8 (*meta* Ar–CH), 106.7 (*para* Ar–CBr), 99.5 (Q *p-*cymene −CCH(CH_3_)_2_), 97.8 (Q *p-*cymene −C(CH_3_)), 93.4 (methine −COCHCO), 84.6 (*p-*cymene Ar–CH), 84.5 (*p-*cymene Ar–CH), 81.9 (Q Cp −CC_4_H_4_), 80.0 (*p-*cymene Ar–CH), 80.0 (*p-*cymene Ar–CH), 71.9 (Cp −CC_4_H_4_), 71.8 (Cp −CC_4_H_4_), 71.0 (Cp −C_5_H_5_), 69.9 (Cp −CC_4_H_48_), 68.8 (Cp −CC_4_H_4_), 31.7 (*p-*cymene −CCH(CH_3_)_2_), 22.7 (*p-*cymene −CCH(CH_3_)_2_), 22.6 (*p-*cymene −CCH(CH_3_)_2_), 17.9 (*p-*cymene −C(CH_3_)). Analysis calculated for C_29_H_28_BrClFeO_2_Ru: C 51.16, H 4.15%. Found: C 51.20, H 4.20%. HR-MS [ES^+^] calculated for C_29_H_28_BrClFeO_2_Ru: 679.935. Found: 644.968 [M^+^]-Cl.

#### Complex **23**

Yield: 0.23 g, 80%; ^1^H NMR (500 MHz, (CD_3_)_2_CO, δ); 8.20 (t, 1H, ^3^*J*(^1^H–^1^H) = 1.6 Hz, *ortho* ArC–H), 7.83 (dt, 1H, ^3^*J*(^1^H–^1^H) = 8.0 Hz, ^4^*J*(^1^H–^1^H) = 1.2 Hz, *ortho* ArC–H), 7.71 (dt, 1H, ^3^*J*(^1^H–^1^H) = 7.8 Hz, ^4^*J*(^1^H–^1^H) = 0.7 Hz, *para* ArC–H), 7.10 (t, 1H, ^3^*J*(^1^H–^1^H) = 7.8 Hz, *meta* ArC–H), 6.01 (s, 1H, methine −COCHCO−), 5.57 (dt, 2H, ^3^*J*(^1^H–^1^H) = 4.81, ^4^*J*(^1^H–^1^H) = 1.3 Hz, *p-*cymene ArC–H), 5.26 (t, 2H, ^3^*J*(^1^H–^1^H) = 5.0 Hz, *p-*cymene ArC–H), 4.85 (t, 1H, ^3^*J*(^1^H–^1^H) = 1.2 Hz, Cp −CC_4_H_4_), 4.76 (t, 1H, ^3^*J*(^1^H–^1^H) = 1.2 Hz, Cp −CC_4_H_4_), 4.34 (m, 1H, Cp −CC_4_H_4_), 4.30 (m, 1H, Cp −CC_4_H_4_), 4.11 (s, 5H, Cp −C_5_H_5_), 2.90 (sept, 1H, ^3^*J*(^1^H–^1^H) = 6.9 Hz, *p-*cymene −CCH(CH_3_)_2_), 2.16 (s, 3H, *p-*cymene −C(CH_3_)), 1.34 (d, 6H, ^3^*J*(^1^H–^1^H) = 7.1 Hz, *p-*cymene −CCH(CH_3_)_2_); ^13^C{^1^H} NMR (125 MHz, (CD_3_)_2_CO, δ); 187.6 (Q C–O), 176.3 (Q C–O), 142.7 (Q Ar–C), 139.9 (*ortho* Ar–CH), 136.9 (*ortho* Ar–CH), 131.0 (*meta* Ar–CH), 127.0 (*para* Ar–CH), 105.9 (*ortho* Ar–CCI), 99.5 (Q *p*-cymene −CCH(CH_3_)_2_), 97.8 (Q *p*-cymene −C(CH_3_)_2_), 93.5 (methine −COCHCO−), 84.5 (*p*-cymene Ar–CH), 84.5 (*p*-cymene Ar–CH), 81.8 (Q −CC_4_H_4_), 80.1 (*p*-cymene Ar–CH), 80.0 (*p*-cymene Ar–CH), 71.9 (Cp −CC_4_H_4_), 71.9 (Cp −CC_4_H_4_), 71.0 (Cp −C_5_H_5_), 70.0 (Cp −CC_4_H_4_), 68.9 (Cp −CC_4_H_4_), 31.7 (*p*-cymene −CCH(CH_3_)_2_), 22.7 (*p*-cymene -CCH(CH_3_)_2_), 22.6 (*p*-cymene −CCH(CH_3_)_2_), 17.8 (*p*-cymene −C(CH_3_)). Analysis calculated for C_29_H_28_ClFeIO_2_Ru: C 47.86, H 3.88%. Found: C 47.89, H 3.72%. HR-MS [ES^+^] calculated for C_29_H_28_ClFeIO_2_Ru: 727.922. Found: 692.954 [M^+^]-Cl.

#### Complex **24**

Yield: 0.29 g, 79%. ^1^H NMR (500 MHz, (CD_3_)_2_CO, δ); 7.66 (br. d, 4H, ^3^*J*(^1^H–^1^H) = 12.8 Hz, *ortho* and *meta* ArC–H), 6.02 (s, 1H, methine −COCHCO−), 5.56 (br. s, 2H, *p-*cymene ArC–H), 5.24 (br. s, 2H, *p-*cymene ArC–H), 4.83 (br. m, 1H, Cp −CC_4_H_4_), 4.73 (br. m, 1H, Cp −CC_4_H_4_), 4.31 (br. d, 2H, ^3^*J*(^1^H–^1^H) = 18.6 Hz, Cp −CC_4_H_4_), 4.11 (s, 5H, Cp −C_5_H_5_), 2.90 (sept, 1H, ^3^*J*(^1^H–^1^H) = 6.8 Hz, *p-*cymene −CCH(CH_3_)_2_), 2.15 (s, 3H, *p-*cymene −C(CH_3_)), 1.34 (d, 6H, ^3^*J*(^1^H–^1^H) = 6.2 Hz, *p-*cymene −CCH(CH_3_)_2_); ^13^C{^1^H} (125 MHz, (CD_3_)_2_CO, δ); 187.1 (Q C–O), 178.8 (Q C–O), 140.2 (Q Ar–C), 138.2 (*ortho* Ar–CH), 129.8 (*meta* Ar–CH), 105.9 (*para* Ar–CI), 99.5 (Q *p-*cymene −CCH(CH_3_)_2_), 97.8 (Q *p-*cymene −C(CH_3_)), 93.3 (methine −COCHCO−), 84.6 (*p-*cymene Ar–CH), 84.5 (*p-*cymene Ar–CH), 81.9 (Q Cp −CC_4_H_4_), 80.0 (*p-*cymene Ar–CH), 80.0 (*p-*cymene Ar–CH), 71.9 (Cp −CC_4_H_4_), 71.8 (Cp −CC_4_H_4_), 71.0 (Cp −C_5_H_5_), 69.9 (Cp −CC_4_H_4_), 68.8 (Cp −CC_4_H_4_), 31.7 (*p-*cymene −CCH(CH_3_)_2_), 22.7 (*p-*cymene −CCH(CH_3_)_2_), 22.6 (*p-*cymene −CCH(CH_3_)_2_), 17.9 (*p-*cymene −C(CH_3_)). Analysis calculated for C_29_H_28_ClFeIO_2_Ru: C 47.86, H 3.88%. Found: C 48.00, H 3.90%. HR-MS [ES^+^] calculated for C_29_H_28_ClFeIO_2_Ru: 727.922. Found: 692.955 [M^+^]-Cl.

## Supporting Information Available

Single-crystal X-ray diffraction data tables and images for all ligands and complexes, NMR spectra for stability studies, cytotoxicity data, UV–vis spectra, cyclic voltammetry, and antimicrobial studies (PDF)
