# Supplementary material for: Cytotoxicity of Ruthenium(II) Arene Complexes Containing Functionalized Ferrocenyl β-Diketonate Ligands
Source: Organometallics. 2023 Jul 5;42(15):1869–81. doi: 10.1021/acs.organomet.2c00553 (PMC10428205; doi:10.1021/acs.organomet.2c00553)
Supplement: Supplementary file 1 — om2c00553_si_001.pdf [file om2c00553_si_001.pdf]

Cytotoxicity of ruthenium(II) arene complexes containing functionalized ferrocenyl β-diketonate ligands

Matthew Allison,[a] Pablo Caramés-Méndez,[a,b] Benjamin J. Hofmann,[c] Christopher M. Pask,[a] Roger M. Phillips,[b] Rianne M. Lord[c,d]* and Patrick C. McGowan[a]*

[a] School of Chemistry, University of Leeds, Woodhouse Lane, Leeds, LS2 9JT, UK, email: p.c.mcgowan@leeds.ac.uk; [b] Department of Pharmacy, University of Huddersfield, Huddersfield, HD1 3DH, UK [c] School of Chemistry, University of East Anglia, Norwich Research Park, Norwich, NR4 7JT, UK, email: r.lord@uea.ac.uk, [d] School of Chemistry and Biosciences, University of Bradford, Bradford, BD7 1DP, UK

Contents

Methods 3

General 3

Instrumentation 3

X-ray Crystallography 3

Cyclic Voltammetry 3

Hydrophobicity 3

96 h Chemosensitivity Studies 4

24 and 48 h Chemosensitivity Studies 4

Chemosensitivity under Hypoxic Conditions 4

Cell Uptake 4

Analysis of cellular DNA damage by the Comet assay 5

Antibacterial HIT Confirmation 6

Antifungal Evaluation 6

Antifungal HIT Confirmation 6

Cytotoxicity Assay against HEK-293 6

Haemolysis Assay 7

Synthesis of Ligands 7

Characterization for Ligands 7

X-ray Crystallography of Ligands 9

X-ray Crystallography of Complexes 13

NMR Ligand and Complex 23

Chemosensitivity Studies 23

Hydrophobicity versus Cytotoxicity 25

Hypoxia Studies 27

UV-vis Stability Studies 27

NMR Stability Studies of Ferrocenyl Ligands 32

Ligands in DMSO 32

Ligands in DMSO + 10% D_2O 35

NMR Stability Studies of Ruthenium Ferrocenyl Complexes 39

Complexes in DMSO 39

Complexes in DMSO + 10% D_2O 43

Complex 1 overlay 47

DMSO + 10% D_2O – showing decomposition 47

DMSO + 10% D_2O + 100 mM NaCl 47

DMSO + 10% D_2O – comparison with and without 100 mM NaCl 48

Cyclic Voltammetry 49

Antimicrobial Studies 54

References 56

Methods

General

Synthetic procedures were conducted under aerobic conditions unless stated otherwise. All chemicals were supplied by Sigma-Aldrich Chemical Co., Acros Organics, Alfa Aesar, Fisher Chemicals and BOC gases used without further purification. Deuterated NMR solvents were purchased from Sigma-Aldrich Chemical Co. or Acros Organics. Chromatography columns were prepared using Fisher Chemicals 60A 35–70 micron silica gel.

Instrumentation

Nuclear magnetic resonance spectra were recorded using Bruker Advance 300, 400, 500, DPX300 and DPX500 MHz spectrometers. Chemical shifts are reported in parts per million (δ) downfield relative to the internal reference tetramethylsilane or referenced to the solvent signal. All NMR spectra were recorded in deuterated acetone at room temperature. Abbreviations used: br. = broad, d = doublet, dd = doublet of doublets, dt = doublet of triplets, m = multiplet, Q = quaternary, q = quartet, s = singlet, t = triplet. Mass spectra were recorded using a micromass ZMD 2000 spectrometer employing the electrospray (ES+/-) ionisation technique. Accurate molecular masses were obtained from Walters LCT, GCT or Bruker MicroTof spectrometers. Microanalyses were acquired by Mr. Stephen Boyer at the London Metropolitan University Elemental Analysis Service. UV-vis absorption spectra were acquired on a Cary Series UV-vis spectrophotometer using 1 cm path length quartz cuvettes.

X-ray Crystallography

A suitable single crystal was selected and immersed in inert oil. The crystal was then mounted to a goniometer head on an Agilent SuperNova X-ray diffractometer fitted with an Atlas area detector and a kappa-geometry 4-circle goniometer, using mirror monochromated Mo-Kα radiation (λ = 0.71073 Å) or Cu-Kα (λ = 1.54184 Å) radiation. The crystal was cooled to 120 K by an Oxford cryostream low temperature device.[1] The full data set was recorded and the images processed using CrysAlis Pro.[2] Structure solution by direct methods was achieved through the use SHELXT and SHELXL programs,[3, 4] and the structural model refined by full matrix least squares on F^2 using the program Olex2.[5] Molecular graphics were plotted, editing of CIFs and construction of tables of bond lengths and angles were achieved using Olex2. Unless otherwise stated, hydrogen atoms were placed using idealized geometric positions (with free rotation for methyl groups), allowed to move in a "riding model" along with the atoms to which they were attached, and refined isotropically. model" along with the atoms to which they were attached and refined isotropically. The SQUEEZE routine in Olex2 and was used to refine structures where diffuse electron density could not be adequately modelled as solvent of crystallization.[6]

Cyclic Voltammetry

A Metrohm Autolab PGSTAT30 potentiostat was used to conduct cyclic voltammetry measurements combined with the Nova software package (Metrohm, Version 2.1.5). A glassy carbon working electrode (diameter = 3 mm) and a glassy carbon rod counter electrode were used. As a reference electrode, a double junction Ag/AgCl electrode was used with a 2 M solution of LiCl in ethanol in the inner compartment and 0.1 M NBu_4PF_6 solution in acetonitrile in the outer compartment. All samples were measured in concentrations of 0.7-0.8 mg/mL for complexes and 0.5-0.6 mg/mL for ligands in 10 mL of a dried and degassed 0.1 M NBu_4PF_6 solution in acetonitrile. The voltammograms were corrected using ferrocene as internal standard with the Fc/Fc^+ couple at 0.40 V vs SCE (standard calomel electrode).[7] The reversible Fc^*/Fc^{*+} couple is measured between -0.5 – 1.0 V with scanning speeds of 50, 100 and 250 mV/s. All other voltammograms are obtained using a scanning speed of 100 mV. The potential was scanned three times and the third run is presented using QtiPlot.

Hydrophobicity

Equal volumes of 1-octanol and sodium chloride saturated distilled water were stirred for 16 h and separated to give water-saturated octanol and octanol-saturated water. Standard solutions of each complex (5, 10, 20, 40 and 60 µM) were prepared in water-saturated octanol. The calibration curve of absorbance against concentration was determined from the maximum absorbance (λ_{max}) of the standard solutions. Stock solutions of each complex (50 µM) in water-saturated octanol (25 mL) were prepared. Six independent samples were prepared by the addition of the stock solution (3 mL) to a 15 mL Falcon tube followed by layered addition of octanol-saturated water (3 mL). Samples were shaken at 1000 g/min for 2 h using an IKA Vibrax VXC basic shaker. The layers were separated, and the water-saturated octanol layer retained. The concentration of each sample in the water-saturated octanol layer was determined by UV-vis spectroscopy with reference to the individual calibration

curves to give an average concentration for shaken samples ($[C]_{final}$). The concentration of an unshaken sample of stock solution was determined to give $[C]_{initial}$. The partition coefficient (Log*P*) was determined with by **Eqn1.**

$$\mathrm{Log}P = \mathrm{Log}_{10}\left(\frac{[\mathrm{C}]_{\mathrm{final}}}{[\mathrm{C}]_{\mathrm{initial}} - [\mathrm{C}]_{\mathrm{final}}}\right) \qquad \textbf{Eqn1}$$

96 h Chemosensitivity Studies

In vitro chemosensitivity tests were performed at both the University of Huddersfield and the Institute of Cancer Therapeutics, Bradford, in a sterile Class II Biological Safety Cabinet fitted with a HEPA filtered laminar airflow. Cellular assays were conducted against human pancreatic carcinoma (MIA PaCa-2), human colorectal carcinoma *p53*-wildtype (HCT116 $p53^{+/+}$), and human non-cancerous epithelial retinal cells (ARPE-19). The ARPE-19 cells were obtained from the American Type Culture Collection and provided by the University of Huddersfield. All cell lines were routinely maintained as monolayer cultures in appropriate complete medium and maintained in either T-25 or T-75 flasks at 37 °C and 5% CO_2. MIA PaCa-2 and ARPE-19 cells were cultured in high glucose DMEM-F12 medium containing 10% foetal calf serum, and HCT116 $p53^{+/+}$ was cultured in RPMI 1640 supplemented with 10% foetal calf serum. All medium was supplemented with sodium pyruvate (1 mM) and L-glutamine (2 mM), and prior to chemosensitivity studies, cell monolayers were passaged using Trypsin-EDTA and diluted to a concentration of 1 x 10^4 cells/mL. All assays were conducted using 96-well plates, in which 100 mL of the 1 x 10^4 cells/mL cell suspension was added to each well and incubated for 24 h at 37 °C and 5% CO_2. After 24 h, 100 mL of drug dilutions (fresh 100 mM stock solutions in DMSO) in media were added to the plates and then incubated for a further 96 h at 37°C and 5% CO_2 (48 h + 48 h recovery period were used for the shorter exposures with MIA PaCa-2 cells only). After 96 h, 20 mL MTT (3-(4,5-dimethylthiazol-2-yl)-2,5-diphenyltetrazolium bromide, 5 mg/mL) was added to each well and incubated for 3 h at 37 °C and 5% CO_2. All solutions were then removed from the 96-well plates via pipette and 150 mL of DMSO added to each well to dissolve the purple formazan crystals. After mixing well, the absorbance of each well was measured at 540 nm using a ThermoFisher Multiskan FC spectrophotometer microplate reader. Using the blank and 100% cell controls, the % cell viability was determined [(abs. of compound containing wells – abs. media wells)/(abs. 100% cell suspension well control)] and from this the % cell death (100%- % cell viability) was calculated. On a logarithmic scale, the percentage cell death was plotted against the concentration of compound, and from this the half maximal inhibitory concentration (IC_{50}) value was determined. Each of the experiments were performed in triplicate with mean values being stated as the IC_{50} ± Standard Deviation (SD).

24 and 48 h Chemosensitivity Studies

The same experimental protocol described for the 96 h MTT assay was used, and the MIA PaCa-2 cells were incubated for either 24 or 48 h, with a 72 or 48 h recovery period respectively.

Chemosensitivity under Hypoxic Conditions

The assay was conducted according to the protocol stated previously for normoxic conditions. However, all the incubations periods, the addition of the drug and the addition of the MTT solution were carried out inside a Don Whitley Scientific H35 Hypoxystation with the oxygen level set at 0.1 %. Cell culture media was conditioned for 24 hours at 0.1 % O_2 prior to the start of the experiment.

Cell Uptake

Cell uptake assays were conducted using MIA PaCa-2 cells and these were maintained as described above. All assays were conducted in 6-well plates and cell concentrations of 1 x 10^5 cells/ well in 2 mL of complete cell media. These cell suspensions were added to the wells and incubated at 37°C and 5% CO_2 for 24 h. The media was then removed, and 2 mL of media/copper compound was added to each well. For each compound, a concentration of 10 µM of ligands L1, L2, L4, L7 or complexes C1, C2, C4, C7 were used. The cells were incubated with the complexes for 48 h, before removing and discarding the media. The cell monolayers were then individually washed with PBS (3 x 1 mL) and all waste discarded. 0.25% Trypsin-EDTA (0.5 mL) was then added until the cells were removed from the surface of the wells and then diluted with media (1.5 mL). The contents of each well were transferred to separate Falcon tubes and centrifuged at 1500 rpm for 5 min. The supernatant was removed, the cell pellets were resuspended in PBS (1 mL) and centrifuged again at 1500 rpm for 5 min (this was completed 3 times). On the final time, the cells were counted before the last centrifuge and then pelleted and store in the freezer for ICP-MS analysis. All assays were conducted in triplicate.

Samples were then taken up in Optima high purity nitric acid, diluted to 10 mL with 1mL rhodium internal standard and 9 mL 18.2 Ultrapure water. The elemental contents of the samples were determined using ICP-MS-QQQ Thermo TQ spectrometer. Measurement of iron mass 56 and ruthenium isotope mass 100 was conducted by comparing solutions of known concentration and intensities. A series of standard calibration curves allows the concentration of each element to be determined. The standards were prepared by weight using 1000 mg/kg reference solutions. Operating conditions: cooling flow rate, 14.0 L/min, auxiliary gas flow rate, 0.8 L/min; sampling Depth 5 mm, additional gas flow 75 %, spray chamber 2.7 degrees, nebulizer flow rate, 1.144 L/min pump speed 15 rpm, RF power of 1,550 W.

Analysis of cellular DNA damage by the Comet assay

Slides containing a layer of agarose were prepared in advance using 1% normal melting point agarose (NMPA, 500 mg/50 mL PBS). A concentration of 1 x 10^6 cells/ mL cell suspension was obtained, and 2 mL added to each well of a 6-well plate. The cells were incubated for 24 hours at 37℃ in a 5.0% CO_2 atmosphere. Drug samples with concentrations ranging from 20-2.5 mM (+ untreated control) were prepared using high glucose DMEM. The medium was removed from the wells, and 2 mL of drug-containing media was added to each well and incubated for a further 48 hours at 37℃ in a 5.0% CO_2 atmosphere. All the drug-containing media was removed and placed in centrifuge tubes, the wells were each washed with PBS (1 mL), which was also placed into the centrifuge tube. The media-free wells were then trypsinized (1 mL) for 3-5 minutes and then neutralized with high glucose DMEM (1 mL), these were all added to the centrifuge tube and centrifuged at 1500 rpm for 3 minutes. The supernatant was removed, and the pellet re-suspended in complete medium (1 mL). The cell suspensions can be stored at -80℃ until required (defrost and centrifuge at 16.1 rcf for 20 seconds). 0.5% low melting agarose (LMPA) was prepared (250 mg/50 mL PBS) and used to re-suspend the pellets (150-1000 mL, depending on size). Cell suspension (150 mL) was added to a previously coated glass slide (containing NMPA) and set using a coverslip and cool tray, the process was repeated with LMPA only. All slides were placed into a tray where freshly prepared neutral lysing solution and incubate for 1 h at 37℃ in dark conditions. The slides were then submerged in electrophoresis buffer for 30 minutes (x2). The slides were then placed in an electrophoresis chamber and freshly prepared electrophoresis buffer (pH 8.0) added and run at 24 V for 25 minutes. The slides were removed and rinsed with distilled water (x 3) and 100% ice cold ethanol (x1), then left to dry overnight. Staining solution (150 mL) was added to each slide and the Comets analysed using Comet assay III software. A minimum of 50 different comets were scored and the computer outputs an average on head and tail intensities and tail moments. Images were taken of the comets and the tail moments plotted against concentration of drug sample used.

Reagents

1. Neutral Lysing Solution (2% sarkosyl, 0.5 M Na_2EDTA, 0.5 mg/ mL proteinase K (pH 8.0)): Sarkosyl (2 g) and Na_2EDTA (18.61 g) were added to distilled water (80 mL) and the pH adjusted to 8.0 with 10 M NaOH. Proteinase K (50 mg) was then added and made up to a final volume of 100 mL.
2. Electrophoresis Buffer (90 mM Tris buffer, 90 mM boric acid, 2 mM Na_2EDTA (pH 8.0)): Tris base (32.707 g), boric acid (16.694 g) and Na_2EDTA (2.233 g) were added to distilled water (2.5 L). The pH was adjusted to 8.0 and the volume made up to 3 L with distilled water.
3. Staining Solution: SYBR™ Gold solution (molecular probes inc, S-11494) (1 mL) was added to PBS (10 mL); this was made on the day of imaging.

Statistical Analysis. Statistical analysis of the results was conducted using Student's t test, for $p < 0.05$ being considered as significant, and $p < 0.01$ as very significant.

Antibacterial Evaluation. Complexes were prepared in DMSO and water to a give a final concentration of 32 mg/mL in 384-well non-binding surface (NBS) plates. The final DMSO concentration was at a maximum of 1.0 %. All bacteria were cultured in Cation adjusted Mueller Hinton broth (CAMHB) at 37 °C overnight. A sample of each culture was diluted 40-fold in fresh broth and incubated at 37 °C for 1.5-3 h. The resultant mid-log phase cultures were diluted (CFU/mL measured by OD_{600}), then added to each well of the compound containing plates, giving a cell density of 5 × 10^5 CFU mL^{-1} and a total volume of 50 µL. Colistin and vancomycin were used as positive bacterial inhibitor standards for Gram-negative and Gram-positive bacteria, respectively. Each standard was provided in four concentrations (above and below the MIC or CC_{50} value) and plated into the first 8 wells of column 23 of the 384-well NBS plates. All the plates were covered and incubated at 37 °C for 18 h without shaking and were carried out in duplicate. Inhibition of bacterial growth was determined measuring absorbance at 600 nm (OD_{600}), using a Tecan M1000 Pro monochromator plate reader. The percentage of growth inhibition was calculated for each well, using the negative control (media only) and positive control (bacteria without inhibitors) on the same plate as references. The significance of the inhibition values was determined by modified Z-scores, calculated using the median and MAD of the samples (no controls) on the same plate. Samples with inhibition

value above 80% and Z-Score above 2.5 for either replicate were classed as actives. Samples with inhibition values in the range 50-80% and Z-Score above 2.5 for either replicate were classed as partial actives.

Antibacterial HIT Confirmation

The preparation is as above, though with maximum DMSO concentrations of 0.5%. Results were analysed using the same methods and using the same controls. The MIC was determined as the lowest concentration at which the growth was fully inhibited, defined by an inhibition ≥ 80%. In addition, the maximal percentage of growth inhibition is reported as D_{Max}, indicating any compounds with partial activity. Hits were classified by MIC ≤ 16 mg/mL or MIC ≤ 10 mM in either replicate.

Antifungal Evaluation

Complexes were prepared in DMSO and water to a give a final concentration of 32 mg/mL in 384-well non-binding surface (NBS) plate. The final DMSO concentration was at a maximum of 1.0%. Fungal strains were cultured for three days on yeast extract-peptone dextrose (YPD) agar at 30 °C. A yeast suspension of 1 x 10^6 to 5 x 10^6 CFU/mL (as determined by OD_{530}) was prepared from five colonies. The suspension was diluted and added to each well of the compound-containing plates giving a final cell density of fungi suspension of 2.5 × 10^3 CFU/mL and a total volume of 50 mL. Fluconazole was used as a positive fungal inhibitor standard and provided in four concentrations (two above and two below the MIC or CC_{50} value) and plated into the first 8 wells of column 23 of the 384-well NBS plates. All the plates were covered and incubated at 35 °C for 36 h without shaking. All experiments were carried out in duplicate. Growth inhibition of *C. albicans* was determined measuring absorbance at 630 nm (OD_{630}) and the growth inhibition of *C. neoformans* was determined measuring the difference in absorbance between 600 and 570 nm ($OD_{600-570}$), after the addition of 0.001 % resazurin and incubation at 35 °C for an additional 2 h. The absorbance was measured using a Biotek Synergy HTX plate reader. The percentage of growth inhibition was calculated for each well, using the negative control (media only) and positive control (fungi without inhibitors) on the same plate. The significance of the inhibition values was determined by modified Z-scores, calculated using the median and MAD of the samples (no controls) on the same plate. Samples with inhibition value above 80% and Z-Score above 2.5 for either replicate were classed as actives. Samples with inhibition values in the range 50-80% and Z-Score above 2.5 for either replicate were classed as partial actives.

Antifungal HIT Confirmation

The preparation is as above, though with maximum DMSO concentrations of 0.5%. Results were analysed using the same methods and using the same controls. The MIC was determined as the lowest concentration at which the growth was fully inhibited, defined by an inhibition ≥ 80% for *C. albicans* and an inhibition ≥ 70% for *C. neoformans*. Due to a higher variance in growth and inhibition, a lower threshold was applied to the data for C. *neoformans*. In addition, the maximal percentage of growth inhibition is reported as D_{Max}, indicating any compounds with marginal activity. Hits were classified by MIC ≤ 16 µg·mL^{-1} or MIC ≤ 10 mM in either replicate.

Cytotoxicity Assay against HEK-293

To assess the cytotoxicity against eukaryotic cells, HEK-293 cells were counted manually in a Neubauer haemocytometer and then plated in the 384-well plates containing the compounds to give a density of 5000 cells/well in a final volume of 50 mL. Dulbecco's modified eagle medium (DMEM) supplemented with 10% FBS was used as growth media and the cells were incubated together with the compounds for 20 hours at 37 °C in 5% CO_2. Tamoxifen was used as a positive cytotoxicity standard in eight concentrations in 2-fold serial dilutions with 50 µg·mL^{-1} the highest concentration. All experiments were performed in duplicate. Cytotoxicity was measured by fluorescence with excitation at 560 nm and emission at 590 nm (F560/590), after addition of 5 µL of resazurin (25 mg/mL) and incubation for a further 3 h at 37 °C in 5% CO_2. The fluorescence intensity was measured using a Tecan M1000 Pro monochromator plate reader, using automatic gain calculation. CC_{50} (concentration at 50%) were calculated by curve fitting the inhibition values against log(concentration) using sigmoidal dose-response function, with variable fitting values for bottom, top and slope. The maximal percentage of cytotoxicity is reported as D_{Max}, indicating any compounds with partial cytotoxicity. The curve fitting was implemented using Pipeline Pilot's dose-response component. Any value with > indicates a sample with no activity (low D_{Max} value) or samples with CC_{50} values above the maximum tested concentration (higher D_{Max} value). Cytotoxic samples were classified by CC_{50} ≤ 32 µg·mL^{-1} or CC_{50} ≤ 10 µM in either replicate. In addition, samples were flagged as partial cytotoxic if D_{Max} ≥ 50%, even with CC_{50} > the maximum tested concentration.

Haemolysis Assay

To assess blood toxicity, human whole blood was washed three times with three volumes of 0.9% NaCl and then resuspended to a concentration of 0.5 x 10^8 cells/mL, as determined by manual cell count in a Neubauer haemocytometer. The washed cells were then added to the 384-well compound-containing plates for a final volume of 50 µL. After a 10 min shake on a plate shaker the plates were then incubated for 1 h at 37 °C. Melittin was used as a positive haemolytic standard in eight concentrations in 2-fold serial dilutions with 50 µg·mL^{-1} the highest concentration. After incubation, the plates were centrifuged at 1000 g for 10 min to pellet cells and debris, 25 µL of the supernatant was then transferred to a polystyrene 384-well assay plate. Haemolysis was determined by measuring the supernatant absorbance at 405 mm (OD405). The absorbance was measured using a Tecan M1000 Pro monochromator plate reader. HC_{10} and HC_{50} (concentration at 10% and 50% haemolysis, respectively) were calculated by curve fitting the inhibition values vs. log(concentration) using a sigmoidal dose-response function with variable fitting values for top, bottom, and slope. In addition, the maximal percentage of haemolysis is reported as D_{Max}, indicating any compounds with partial haemolysis. The curve fitting was implemented using Pipeline Pilot's dose-response component. Any value with > indicate sample with no activity (low D_{Max} value) or samples with HC_{10} values above the maximum tested concentration (higher D_{Max} value). Haemolysis samples were classified by $HC_{10} \leq 32$ µg/mL or $HC_{10} \leq 10$ µM in either replicate. In addition, samples were flagged as partial haemolytic if $D_{Max} \geq 50\%$, even with HC_{10} > the maximum tested concentration.

Synthesis of Ligands

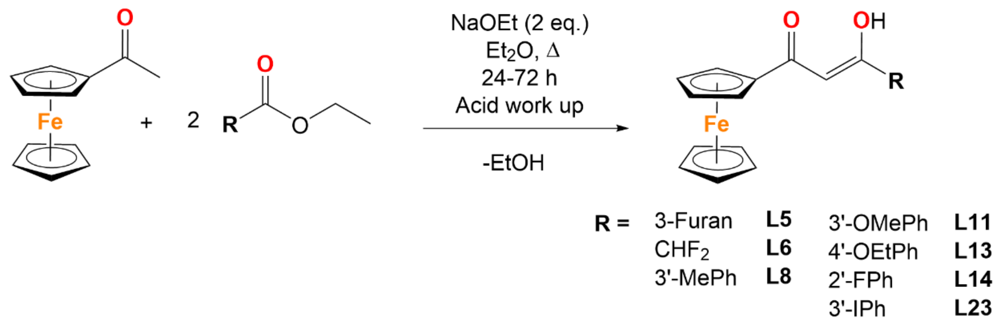

Scheme S 1: Synthetic route for ferrocenyl β-diketonate (Fc-acac) ligands L5, L6, L8, L11, L13, L13 and-L23, where all other ligands have been previously reported.[6,7]

Characterization for Ligands

A functionalized ethyl ester (13.0 mmol) was added to a stirred solution of acetyl ferrocene (7.2 mmol) and sodium ethoxide (13.0 mmol) in ether (20 mL). The solution was stirred at reflux for 24-72 h after which time the product was isolated by one of two methods. **1)** The solid precipitate was isolated by filtration, dissolved in distilled water (150 mL) and acidified with 10% hydrochloric acid until pH 5 which caused a red solid to precipitate out in solution. The solid was filtered and dried overnight under vacuum before purification. **2)** The solution was acidified with 10% hydrochloric acid until pH 5 and added to water (50 ml). The product was extracted with ether (3 x 20 mL) and the organic layers were combined, dried over $MgSO_4$ and filtered. Solvent was removed *in vacuo* to give a red solid product. [8, 9]

Ligand L5. Prepared using ethyl-3-furoate (13.0 mmol) and refluxing for 48 h, then worked up following **method 1**. The product was purified by column chromatography and eluting with 90:10 *v/v* hexane/ethyl acetate. The solvent was removed to yield the product as a red solid. **Yield:** 0.71 g, 31%; **^{1}H NMR (500 MHz, $(CD_3)_2CO$, δ);** 8.17 (s, 1H, Furan -CCH(O)$C_2\underline{H}_2$), 7.57 (t, 1H, $^3J(^1H\text{-}^1H)$ = 1.6 Hz, Furan -CC$\underline{H}$(O)C_2H_2), 6.83 (d, 1H, $^3J(^1H\text{-}^1H)$ = 1.2 Hz, Furan -CCH(O)$C_2\underline{H}_2$), 6.35 (s, 1H, methine -COC$\underline{H}$CO-), 4.83 (t, 2H, $^3J(^1H\text{-}^1H)$ = 1.8 Hz, Cp -$CC_4\underline{H}_4$), 4.46 (t, 2H, $^3J(^1H\text{-}^1H)$ = 1.8 Hz, -$CC_4\underline{H}_4$), 4.09 (s, 5H, Cp -$C_5\underline{H}_5$); **^{13}C{^{1}H} NMR (125 MHz, $(CD_3)_2CO$, δ);** 193.9 (Q $\underline{C}$-O), 176.7 (Q $\underline{C}$-O), 150.4 (Q Furan -$\underline{C}CHOC_2H_2$), 146.3 (Furan -C$\underline{C}HOC_2H_2$), 145.5 (Furan -CCH(O)$\underline{C}_2H_2$), 109.0 (Furan -CCH(O)$\underline{C}_2H_2$), 95.4 (methine -CO$\underline{C}$HCO-), 79.0 (Q Cp -$\underline{C}C_4H_4$), 73.0 (Cp -C$\underline{C}_4H_4$), 71.1 (Cp -$\underline{C}_5H_5$), 69.6 (Cp -C$\underline{C}_4H_4$); **Analysis Calculated for $C_{17}H_{14}FeO_3$:** C 63.38, H 4.38%, **Found:** C 63.27, H 4.47%; **HR-MS [ES$^+$] Calculated for $C_{17}H_{14}FeO_3$:** 322.0292, **Found:** 322.029 [M$^+$].

Ligand L6. Prepared using ethyl difluoroacetate (13.0 mmol) and refluxing for 24 h, then worked up following **method 1**. The product precipitated out as a pure red solid. **Yield:** 2.13 g, 97%;.**^{1}H NMR (500 MHz, $(CD_3)_2CO$, δ);** 6.17 (s, 1H, methine -COCHCO-), 4.87 (t, 2H, $^3J(^1H-^1H)$ = 1.8 Hz, Cp -CC_4H_4), 4.60 (t, 2H, $^3J(^1H-^1H)$ = 1.8 Hz, Cp -CC_4H_4), 4.22 (s, 1H, -CHF_2), 4.13 (s, 5H, Cp -C_5H_5); **$^{13}C\{^1H\}$ (125 MHz, $(CD_3)_2CO$, δ);** 197.0 (Q C-O), 163.6 (Q C-O), 111.3 (t, -CHF_2, $^1J(^{13}C-^{19}F)$ = 242.6 Hz), 95.8 (methine -COCHCO-), 77.8 (Q Cp -CC_4H_4), 74.3 (Cp -CC_4H_4), 71.4 (Cp -C_5H_5), 70.1 (Cp -CC_4H_4); **Analysis Calculated for $C_{14}H_{12}F_2FeO_2$:** C 54.94, H 3.95%, **Found:** C 54.40, H 3.90%; **HR-MS [ES$^+$] Calculated for $C_{14}H_{12}F_2FeO_2$:** 306.016, **Found:** 307.022 [MH^+].

Ligand L8. Prepared using ethyl-m-toluate (13.0 mmol) and refluxing for 24 h, then worked up following **method 2**. The product was purified by column chromatography and eluting with 85:15 *v/v* hexane/ethyl acetate. The solvent was removed to give the product as a red solid. **Yield:** 1.73 g, 69%; **^{1}H NMR (500 MHz, $(CD_3)_2CO$, δ);** 7.91 (s, 1H, *ortho* ArC-H), 7.87 (t, 1H, $^3J(^1H-^1H)$ = 3.6 Hz, *meta* ArC-H), 7.43 (d, 2H, $^3J(^1H-^1H)$ = 4.6 Hz, *ortho* and *para* ArC-H), 6.77 (s, 1H, methine -COCHCO-), 5.06 (t, 2H, $^3J(^1H-^1H)$ = 1.7 Hz, Cp -CC_4H_4), 4.65 (t, 2H, $^3J(^1H-^1H)$ = 1.7 Hz, Cp -CC_4H_4), 4.26 (s, 5H, Cp -C_5H_5), 2.45 (s, 3H, *meta* Ar-CCH_3); **$^{13}C\{^1H\}$ (125 MHz, $(CD_3)_2CO$, δ);** 195.4 (Q C-O), 180.4 (Q C-O), 139.2 (Q Ar-C), 136.0 (Q *meta* Ar-C(CH_3)), 133.5 (ArC-H), 129.4 (ArC-H), 128.1 (ArC-H), 124.8 (ArC-H), 94.6 (methine -COCHCO-), 79.3 (Q Cp -CC_4H_4), 73.2 (Cp -C_5H_5), 71.1 (Cp -CC_4H_4), 69.8 (Cp -CC_4H_4), 21.4 (*meta* Ar-C(CH_3)); **Analysis Calculated for $C_{20}H_{18}FeO_2$:** C 69.39, H 5.24%, **Found:** C 69.39, H 5.31%; **HR-MS [ES$^+$] Calculate for $C_{20}H_{18}FeO_2$:** 346.066, **Found:** 346.066 [M^+].

Ligand L11. Prepared using ethyl-3-methoxybenzoate (13.0 mmol) and refluxing for 24 h, then worked up following **method 2**. The product was purified by column chromatography and eluting with 80:20 *v/v* hexane/ethyl acetate. The solvent was removed to yield the product as a red solid. **Yield:** 1.67 g, 64%; **^{1}H NMR (500 MHz, $(CD_3)_2CO$, δ);** 7.66 (d, 1H, $^3J(^1H-^1H)$ = 7.6 Hz, *ortho* ArC-H, H_{19}), 7.58 (s, 1H, *ortho* ArC-H), 7.46 (t, 1H, $^3J(^1H-^1H)$ = 8.0 Hz, *meta* ArC-H), 7.17 (dd, 1H, $^3J(^1H-^1H)$ = 8.0 Hz, $^4J(^1H-^1H)$ = 2.3 Hz, *para* ArC-H), 6.77 (s, 1H, methine -COCHCO-), 5.08 (s, 2H, Cp -CC_4H_4), 4.65 (s, 2H, Cp -CC_4H_4), 4.26 (s, 5H, Cp -C_5H_5), 3.92 (s, 3H, *meta* Ar-C(OCH_3)); **$^{13}C\{^1H\}$ NMR (125 MHz, $(CD_3)_2CO$, δ);** 195.4 (Q C-O), 180.2 (Q C-O), 161.0 (Q *meta* Ar-C(OCH_3), C_{16}), 137.5 (Q Ar-C), 130.6 (*ortho* Ar-CH), 120.0 (*ortho* Ar-CH), 118.4 (*para* Ar-CH), 112.8 (*meta* Ar-CH), 94.8 (methine -COCHCO-), 79.2 (Q Cp -CC_4H_4), 73.2 (Cp -CC_4H_4), 71.1 (Cp -C_5H_5), 69.8 (Cp -CC_4H_4), 55.8 (*meta* Ar-C(OCH_3)); **Analysis Calculated for $C_{20}H_{18}FeO_3$:** C 66.32, H 5.01%, **Found:** C 66.19, H 5.12%; **HR-MS [ES$^+$] Calculated for $C_{20}H_{18}FeO_2$:** 362.061, **Found:** 362.061 [M^+].

Ligand L13. Prepared using ethyl-4-ethoxybenzoate (13.0 mmol) and refluxing for 24 h, then worked up following **method 1**. The product was purified by column chromatography and eluting with 87:13 *v/v* hexane/ethyl acetate. The solvent was removed to yield the product as a red solid. **Yield:** 0.38 g, 14%; **^{1}H NMR (500 MHz, $(CD_3)_2CO$, δ);** 8.04 (d, 2H, $^3J(^1H-^1H)$ = 2.1 Hz, *ortho* ArC-H), 7.05 (d, 2H, $^3J(^1H-^1H)$ = 2.3 Hz, *meta* ArC-H), 6.72 (s, 1H, methine -COCHCO-), 5.05 (br. s, 2H, Cp -CC_4H_4), 4.62 (br. s, 2H, Cp -CC_4H_4), 4.25 (s, 5H, Cp -C_5H_5), 4.18 (m, 2H, *para* Ar-C(OCH_2CH_3)), 1.43 (t, 3H, $^3J(^1H-^1H)$ = 3.3 Hz, *para* Ar-C(OCH_2CH_3)); **$^{13}C\{^1H\}$ NMR (125 MHz, $(CD_3)_2CO$, δ);** 193.7 (Q C-O), 181.4 (Q C-O), 163.3 (*para* Ar-C(OCH_2CH_3)), 132.1 (Q Ar-C), 129.7 (*ortho* Ar-CH), 115.3 (*meta* Ar-CH), 93.4 (methine -COCHCO-), 79.4 (Q Cp -CC_4H_4), 72.9 (Cp -CC_4H_4), 71.0 (Cp -C_5H_5), 69.6 (Cp -CC_4H_4), 64.5 (*para* Ar-C(OCH_2CH_3)), 15.0 (*para* Ar-C(OCH_2CH_3)); **Analysis Calculated for $C_{21}H_{20}FeO_3$:** C 67.04, H 5.36%, **Found:** C 67.10, H 5.40%; **HR-MS [ES$^+$] Calculated for $C_{21}H_{20}FeO_3$:** 376.076, **Found:** 377.083 [MH^+].

Ligand L14. Prepared using ethyl-2-fluorobenzoate (13.0 mmol) and refluxing for 72 h, then worked up following **method 2**. The product was purified by column chromatography and eluting with 90:10 *v/v* hexane/ethyl acetate. The solvent was removed to yield the product as a red solid. **Yield:** 1.17 g, 47%; **^{1}H NMR (500 MHz, $(CD_3)_2CO$, δ);** 7.84 (td, 1H, $^3J(^1H-^1H)$ = 7.8 Hz, $^4J(^1H-^1H)$ = 1.6 Hz, *ortho* ArC-H), 7.47 (m, 1H, *meta* ArC-H), 7.22 (m, 1H, *meta* ArC-H), 7.16 (m, 1H, *para* ArC-H), 6.47 (s, 1H, methine -COCHCO-), 4.82 (q, 2H, $^3J(^1H-^1H)$ = 2.1 Hz, -CC_4H_4), 4.52 (q, 2H, $^3J(^1H-^1H)$ = 1.8 Hz, -CC_4H_4), 4.11 (s, 5H, -C_5H_5); **$^{13}C\{^1H\}$ NMR (125 MHz, $(CD_3)_2CO$, δ);** 196.3 (Q C-O), 175.4 (d, Q C-O, $^3J(^{13}C-^{19}F)$ = 3.1 Hz), 161.7 (d, *ortho* Ar-CF, $^1J(^{13}C-^{19}F)$ = 253.3 Hz), 134.2 (d, *para* Ar-CH, $^3J(^{13}C-^{19}F)$ = 8.8 Hz), 130.6 (d, *ortho* Ar-CH, $^3J(^{13}C-^{19}F)$ = 2.1 Hz), 125.6 (d, *meta* Ar-CH, $^4J(^{13}C-^{19}F)$ = 3.1 Hz), 124.2 (d, Q Ar-C, $^2J(^{13}C-^{19}F)$ = 10.4 Hz), 117.4 (d, *meta* Ar-CH, $^2J(^{13}C-^{19}F)$ = 23.4 Hz), 99.6 (d, methine -COCHCO-, $^4J(^{13}C-^{19}F)$ = 11.9 Hz), 79.0 (Q -CC_4H_4), 73.6 (Cp -CC_4H_4), 71.2 (Cp -C_5H_5, 69.8 (Cp -CC_4H_4); **Analysis Calculated for $C_{19}H_{15}FFeO_2$:** C 65.17, H 4.32%, **Found:** C 65.10, H 4.30%; **HR-MS [ES$^+$] Calculated for $C_{19}H_{15}FFeO_2$:** 350.041, **Found:** 351.049 [MH^+].

Ligand L23. Prepared using ethyl-3-iodobenzoate (13.0 mmol) and refluxing for 24 h, then worked up following **method 1**. The product was purified by column chromatography and eluting with 90:10 *v/v* hexane/ethyl acetate. The solvent was removed to yield the product as a red solid. **Yield:** 1.02 g, 31%; **^{1}H NMR (500 MHz, $(CD_3)_2CO$, δ);** 8.41 (t, 1H, $^3J(^1H-^1H)$ = 1.4 Hz, *ortho* ArC-H), 8.11 (dd, 1H, $^3J(^1H-^1H)$ = 7.8 Hz, $^4J(^1H-^1H)$ = 0.8 Hz, *ortho* ArC-H), 7.98 (dt, 1H, $^3J(^1H-^1H)$ = 7.8 Hz, $^4J(^1H-^1H)$ = 0.8 Hz, *para* ArC-H), 7.36 (t, 1H, $^3J(^1H-^1H)$ = 7.9 Hz, *ortho* ArC-H), 6.83 (s, 1H, methine -COCHCO-), 5.09 (t, 2H, $^3J(^1H-^1H)$ = 1.8 Hz, Cp -CC_4H_4), 4.67 (t, 2H, $^3J(^1H-^1H)$ = 1.8 Hz, Cp -CC_4H_4), 4.27 (s, 5H, Cp -C_5H_5); **$^{13}C\{^1H\}$ NMR (125 MHz, $(CD_3)_2CO$, δ);** 195.9 (Q C-O), 178.3 (Q C-O), 142.9 (Q Ar-C), 141.5 (*ortho* Ar-CH), 138.3 (*meta* Ar-CI), 136.2 (*ortho* Ar-CH), 131.5 (*para* Ar-CH), 127.0 (*meta* Ar-CH), 94.8 (methine -COCHCO-), 79.0 (Q Cp -CC_4H_4), 73.5 (Cp -CC_4H_4), 71.2 (Cp -C_5H_5), 69.9 (Cp -CC_4H_4); **Analysis Calculated for $C_{19}H_{15}FeIO_2$:** C 49.82, H 3.30%, **Found:** C 49.75, H 3.23%; **HR-MS [ES$^+$] Calculated for $C_{19}H_{15}FeIO_2$:** 457.947, **Found:** 457.946 [M$^+$].

X-ray Crystallography of Ligands

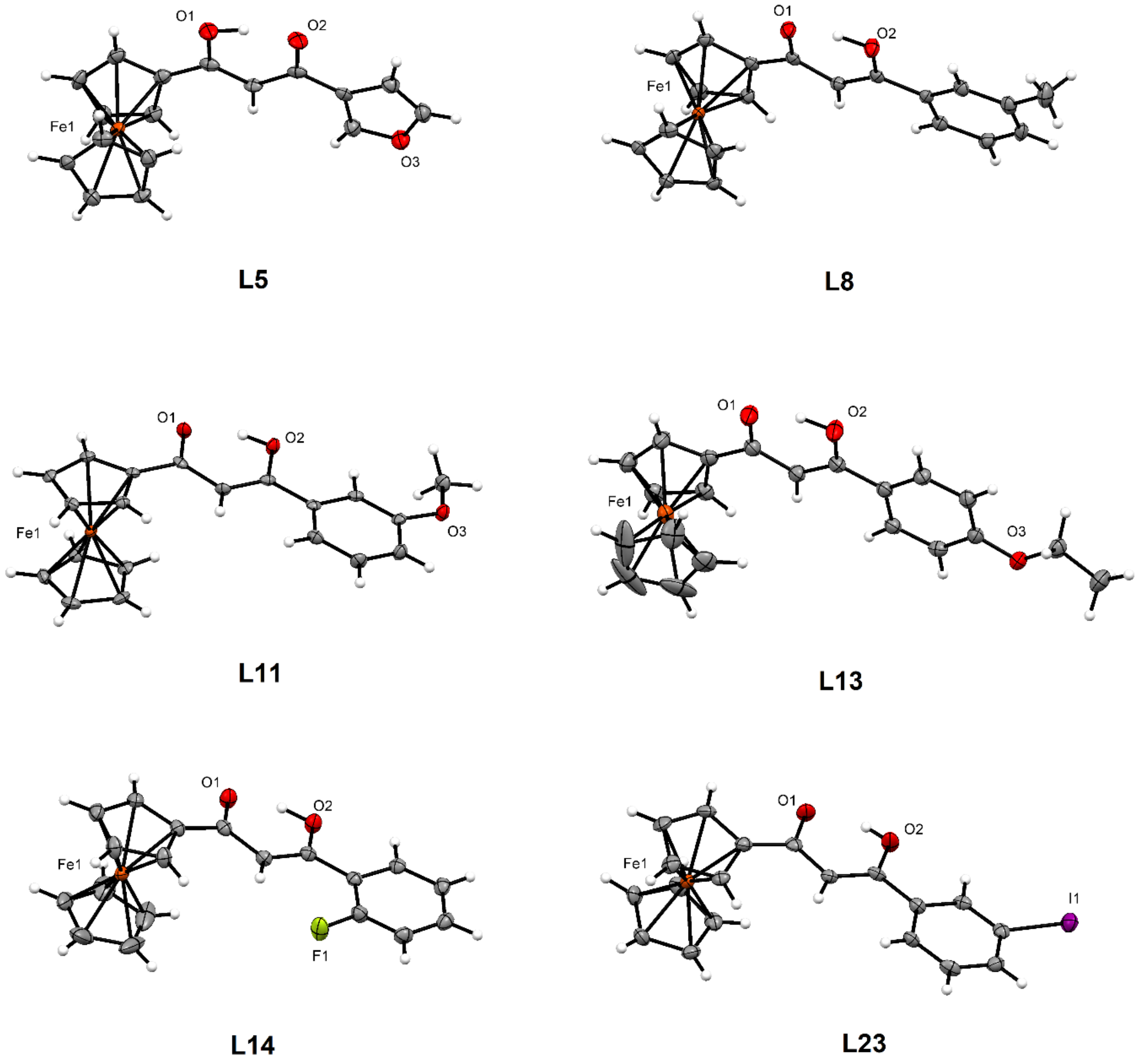

Figure S 1: Molecular structures for ligands L5, L8, L11, L13, L14 and L23. Displacement ellipsoids are placed at the 50% probability level and shown only for the heteroatoms.

Table S 1: X-ray crystallographic data for ligands L5, L8 and L11, s.u.s in parethesis.

Ligand	L5	L8	L11
**CSD Number**	**2052932**	**2052937**	**2052935**
**Empirical formula**	$C_{17}H_{14}FeO_3$	$C_{20}H_{18}FeO_2$	$C_{20}H_{18}FeO_3$
**Formula weight**	322.144	346.210	362.19
**Temperature/K**	119.99(14)	120.01(16)	120.0(2)
**Crystal system**	tetragonal	monoclinic	orthorhombic
**Space group**	*I*-4	$P2_1/n$	*P*bca
**a/Å**	21.6322(7)	5.8265(3)	7.8775(4)
**b/Å**	21.6322(7)	20.8270(11)	12.2908(7)
**c/Å**	5.9033(4)	12.9013(7)	32.9355(18)
**α/°**	90	90	90
**β/°**	90	96.138(5)	90
**γ/°**	90	90	90
**Volume/Å^3**	2762.5(2)	1556.57(15)	3188.9(3)
**Z**	8	4	8
**ρ_{calc}g/cm^3**	1.549	1.477	1.509
**μ/mm^{-1}**	1.098	0.975	0.961
**F(000)**	1331.5	721.7	1504.0
**Crystal size/mm^3**	0.12 × 0.09 × 0.08	0.54 × 0.25 × 0.19	0.28 × 0.16 × 0.05
**Radiation**	Mo Kα (λ = 0.71073)	Mo Kα (λ = 0.71073)	Mo Kα (λ = 0.71073)
**2Θ range for data collection/°**	7.16 to 59.6	6.36 to 59.64	6.266 to 59.606
**Index ranges**	-24 ≤ h ≤ 28, -29 ≤ k ≤ 19, -8 ≤ l ≤ 5	-7 ≤ h ≤ 7, -28 ≤ k ≤ 24, -14 ≤ l ≤ 17	-10 ≤ h ≤ 10, -17 ≤ k ≤ 16, -37 ≤ l ≤ 41
**Reflections collected**	4289	9446	14953
**Independent reflections**	2770 [R_{int} = 0.0331, R_{sigma} = 0.0667]	3809 [R_{int} = 0.0525, R_{sigma} = 0.0732]	4058 [R_{int} = 0.0581, R_{sigma} = 0.0634]
**Data/restraints/parameters**	2770/0/246	3809/0/280	4058/0/289
**Goodness-of-fit on F^2**	1.006	1.061	1.087
**Final R indexes [I>=2σ (I)]**	R_1 = 0.0378, wR_2 = 0.0673	R_1 = 0.0444, wR_2 = 0.0827	R_1 = 0.0537, wR_2= 0.0752
**Final R indexes [all data]**	R_1 = 0.0429, wR_2 = 0.0698	R_1 = 0.0647, wR_2 = 0.0956	R_1 = 0.0795, wR_2 = 0.0830
**Largest diff. peak/hole / e Å^{-3}**	0.39/-0.30	0.68/-0.65	0.45/-0.39

Table S 2: X-ray crystallographic data for ligands L13, L14 and L23, s.u.s in parenthesis.

Ligand	L13	L14	L23
**CSD Number**	**2052934**	**2052933**	**2052936**
**Empirical formula**	$C_{21}H_{20}FeO_3$	$C_{19}H_{15}FFeO_2$	$C_{19}H_{15}FeIO_2$
**Formula weight**	376.236	350.173	458.06
**Temperature/K**	120.01(10)	120.00(10)	120.03(10)
**Crystal system**	monoclinic	monoclinic	tetragonal
**Space group**	$P2_1/c$	$P2_1/c$	$P4_2/n$
**a/Å**	7.3790(3)	6.6136(3)	23.3223(6)
**b/Å**	19.9952(9)	11.4565(5)	23.3223(6)
**c/Å**	11.5786(5)	20.1504(8)	6.0700(2)
**α/°**	90	90	90
**β/°**	94.360(4)	99.109(4)	90
**γ/°**	90	90	90
**Volume/Å^3**	1703.41(13)	1507.51(11)	3301.6(2)
**Z**	4	4	8
**ρcalcg/cm^3**	1.467	1.543	1.843
**μ/mm^{-1}**	0.902	1.017	2.790
**F(000)**	785.8	721.8	1792.0
**Crystal size/mm^3**	0.36 × 0.18 × 0.13	0.4 × 0.29 × 0.17	0.34 × 0.14 × 0.06
**Radiation**	Mo Kα (λ = 0.71073)	Mo Kα (λ = 0.71073)	Mo Kα (λ = 0.71073)
**2Θ range for data collection/°**	6.66 to 59.38	6.9 to 59.54	6.936 to 59.482
**Index ranges**	-8 ≤ h ≤ 10, -27 ≤ k ≤ 26, -15 ≤ l ≤ 16	-8 ≤ h ≤ 8, -15 ≤ k ≤ 14, -25 ≤ l ≤ 28	-30 ≤ h ≤ 31, -28 ≤ k ≤ 32, -6 ≤ l ≤ 8
**Reflections collected**	12232	9608	13335
**Independent reflections**	4135 [R_{int} = 0.0433, R_{sigma} = 0.0508]	3683 [R_{int} = 0.0239, R_{sigma} = 0.0295]	4129 [R_{int} = 0.0610, R_{sigma} = 0.0734]
**Data/restraints/parameters**	4135/0/302	3683/0/268	4129/0/209
**Goodness-of-fit on F^2**	1.069	1.050	0.935
**Final R indexes [I>=2σ (I)]**	R_1 = 0.0473, wR_2 = 0.0989	R_1 = 0.0314, wR_2 = 0.0777	R_1 = 0.0472, wR_2 = 0.1205
**Final R indexes [all data]**	R_1 = 0.0607, wR_2 = 0.1075	R_1 = 0.0371, wR_2 = 0.0821	R_1 = 0.0938, wR_2 = 0.1650
**Largest diff. peak/hole / e Å^{-3}**	0.69/-0.53	0.36/-0.38	1.87/-0.76

Table S 3: Selected bond lengths (Å) for ligands L4-L5, L7-L18 and L20-L24, s.u.s are stated in parenthesis.

**Bond lengths (Å)**	**C11-O1**	**C11-C12**	**C12-C13**	**C13-O2**
**L4**	1.319(5)	1.398(6)	1.390(6)	1.300(6)
**L8**	1.280(3)	1.423(3)	1.371(3)	1.320(3)
**L11**	1.263(3)	1.433(4)	1.369(4)	1.322(3)
**L13**	1.275(3)	1.427(3)	1.374(3)	1.318(3)
**L14**	1.2703(19)	1.431(2)	1.376(2)	1.3275(19)
**L23**	1.295(6)	1.415(7)	1.367(7)	1.317(6)

Table S 4: Selected bond angles (°) for ligands L4-L5, L7-L18 and L20-L24, s.u.s are stated in parenthesis.

**Bond angles (°)**	**O1-C11-C12**	**C11-C12-C13**	**C12-C13-O2**
**L4**	119.6(4)	120.9(4)	121.6(4)
**L8**	120.6(2)	121.7(2)	120.0(2)
**L11**	120.8(2)	120.4(2)	120.9(2)
**L13**	121.1(2)	119.9(2)	120.1(2)
**L14**	121.18(14)	119.44(14)	120.16(14)
**L23**	120.3(5)	120.2(5)	121.1(5)

X-ray Crystallography of Complexes

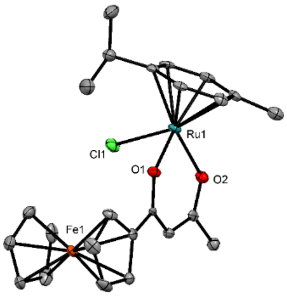

1

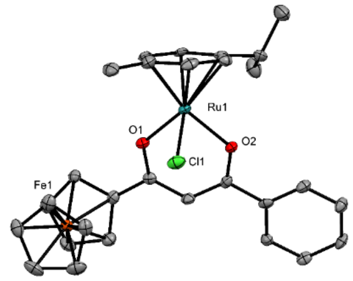

2

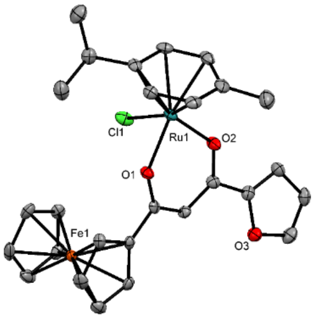

4

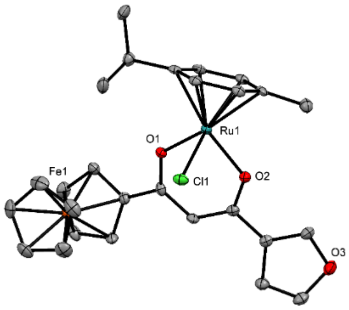

5

Figure S 2: Molecular structures for compounds 1, 2, 4 and 5. Displacement ellipsoids are placed at the 50% probability level and shown only for the heteroatoms.

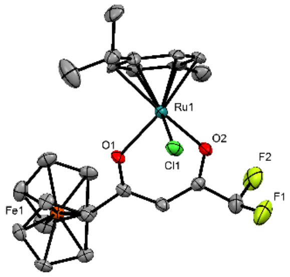

6

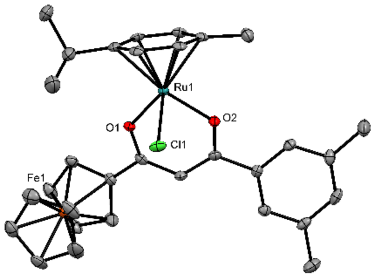

9

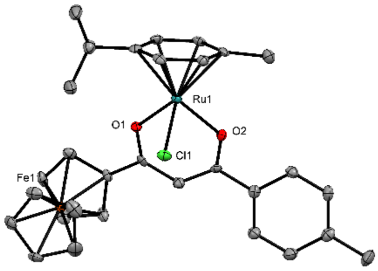

10

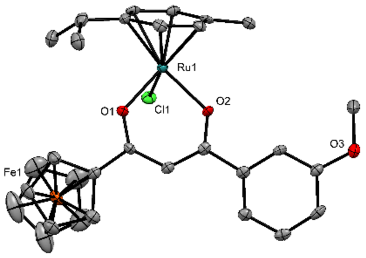

11

Figure S 3: Molecular structures for compounds 6, 9, 10 and 11. Displacement ellipsoids are placed at the 50% probability level and shown only for the heteroatoms.

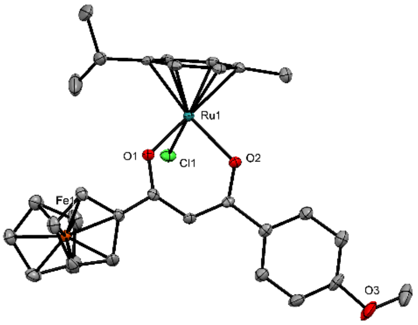

12

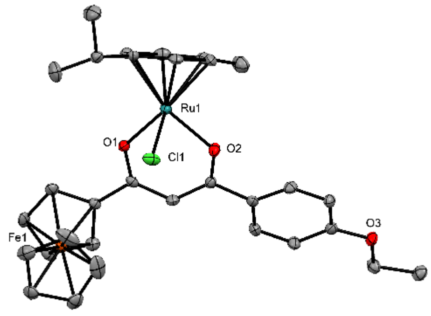

13

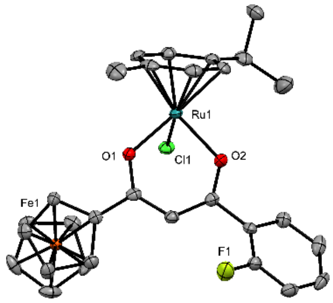

14

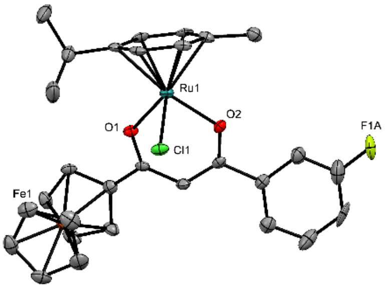

15

Figure S 4: Molecular structures for compounds 12-15. Displacement ellipsoids are placed at the 50% probability level and shown only for the heteroatoms.

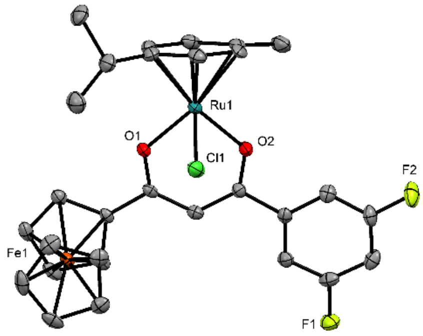

16

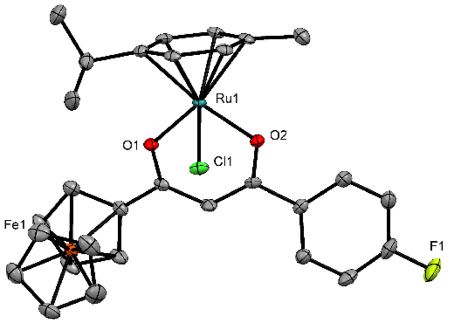

17

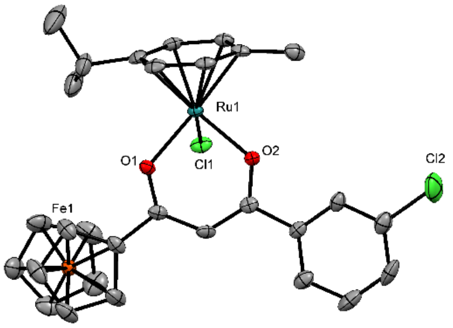

18

Figure S 5: Molecular structures for compounds 16-18. Displacement ellipsoids are placed at the 50% probability level and shown only for the heteroatoms.

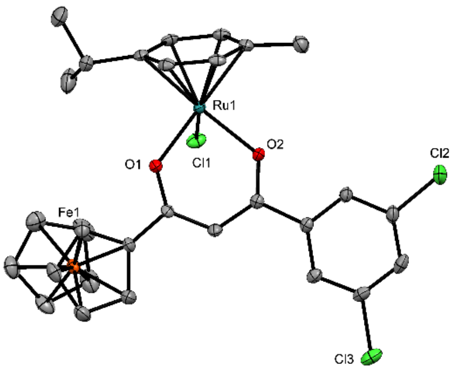

19

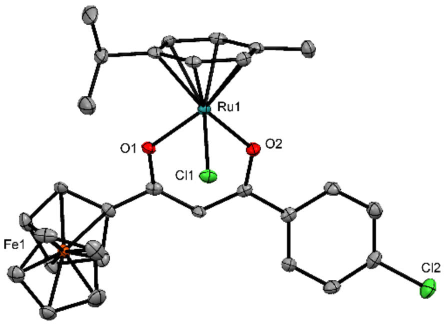

20

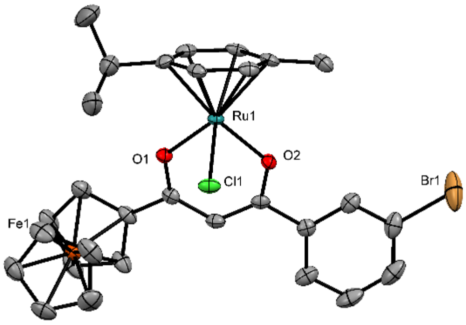

21

Figure S 6: Molecular structures for compounds 19-21. Displacement ellipsoids are placed at the 50% probability level and shown only for the heteroatoms.

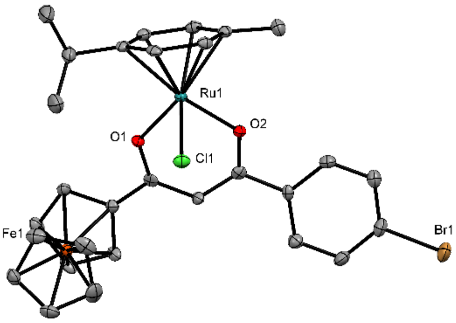

22

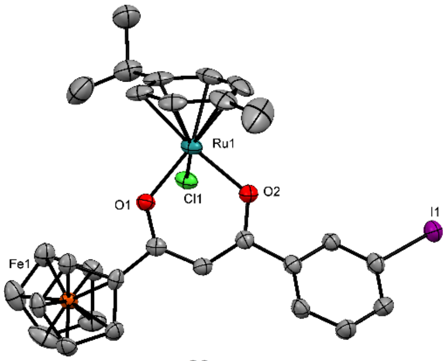

23

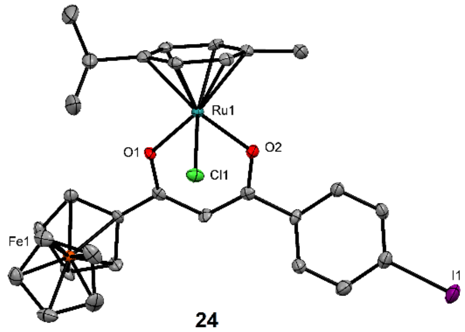

24

Figure S 7: Molecular structures for compounds 22-24. Displacement ellipsoids are placed at the 50% probability level and shown only for the heteroatoms.

Table S 5: X-ray crystallographic data for compounds 1, 2 and 4, s.u.s in parenthesis.

Compound	1	2	4	5
**CSD Number**	**2055570**	**2055565**	**2055584**	**2055572**
**Empirical formula**	$C_{24}H_{27}ClFeO_2Ru$	$C_{29}H_{29}ClFeO_2Ru$	$C_{27}H_{27}ClFeO_3Ru$	$C_{29}H_{30}ClFeNO_3Ru$
**Formula weight**	539.82	601.89	591.85	632.91
**Temperature/K**	120.0(2)	120.03(14)	120.00(17)	120.00(10)
**Crystal system**	monoclinic	monoclinic	monoclinic	monoclinic
**Space group**	$P2_1/c$	$P2_1/n$	$P2_1/n$	$P2_1/c$
**a/Å**	7.4872(3)	17.2048(9)	10.3157(10)	12.5799(3)
**b/Å**	16.7613(9)	7.8568(3)	7.7100(5)	11.0035(2)
**c/Å**	34.6431(16)	19.8481(10)	29.976(3)	18.9357(5)
**α/°**	90	90	90	90
**β/°**	93.867(4)	114.839(6)	98.403(8)	100.596(3)
**γ/°**	90	90	90	90
**Volume/Å^3**	4337.7(4)	2434.8(2)	2358.5(4)	2576.43(11)
**Z**	8	4	4	4
**ρcalcg/cm^3**	1.653	1.642	1.667	1.632
**μ/mm^{-1}**	1.506	1.351	1.396	1.285
**F(000)**	2192.0	1224.0	1200.0	1288.0
**Crystal size/mm^3**	0.073 × 0.034 × 0.022	0.17 × 0.14 × 0.04	0.09 × 0.07 × 0.01	0.17 × 0.12 × 0.09
**Radiation**	Mo Kα (λ = 0.71073)	MoKα (λ = 0.71073)	MoKα (λ = 0.71073)	MoKα (λ = 0.71073)
**2Θ range for data collection/°**	3.384 to 59.432	5.278 to 59.748	5.956 to 59.464	6.198 to 59.708
**Index ranges**	-10 ≤ h ≤ 10, -22 ≤ k ≤ 22, -45 ≤ l ≤ 48	-24 ≤ h ≤ 23, -9 ≤ k ≤ 10, -23 ≤ l ≤ 24	-8 ≤ h ≤ 13, -10 ≤ k ≤ 8, -39 ≤ l ≤ 39	-16 ≤ h ≤ 17, -15 ≤ k ≤ 15, -25 ≤ l ≤ 21
**Reflections collected**	38681	21143	17457	20086
**Independent reflections**	10850 [R_{int} = 0.0750, R_{sigma} = 0.1013]	6045 [Rint = 0.0765, Rsigma = 0.0776]	5933 [R_{int} = 0.0472, R_{sigma} = 0.0607]	6323 [R_{int} = 0.0447, R_{sigma} = 0.0543]
**Data/restraints/parameters**	10850/0/531	6045/0/419	5933/0/301	6323/0/445
**Goodness-of-fit on F^2**	1.093	1.103	1.183	1.093
**Final R indexes [I>=2σ (I)]**	R_1 = 0.0640, wR_2 = 0.0755	R1 = 0.0434, wR2 = 0.0839	R_1 = 0.0565, wR_2 = 0.0824	R_1 = 0.0349, wR_2 = 0.0636
**Final R indexes [all data]**	R_1 = 0.1076, wR_2 = 0.0844	R1 = 0.0644, wR2 = 0.0994	R_1 = 0.0799, wR_2 = 0.0884	R_1 = 0.0525, wR_2 = 0.0728
**Largest diff. peak/hole / e Å^{-3}**	0.69/-0.65	1.00/-0.76	0.65/-0.76	1.13/-0.62

Table S 6: X-ray crystallographic data for compounds 6, 9, 10 and 11, s.u.s in parenthesis.

**Compound**	**6**	**9**	**10**	**11**
**CSD Number**	**2055566**	**2055573**	**2055585**	**2055567**
**Empirical formula**	$C_{24}H_{24}ClF_2FeO_2Ru$	$C_{31}H_{33}ClFeO_2Ru$	$C_{30}H_{31}ClFeO_2Ru$	$C_{30}H_{31}ClFeO_3Ru$
**Formula weight**	574.80	629.969	615.92	631.92
**Temperature/K**	119.97(16)	119.97(13)	120.02(10)	120.01(10)
**Crystal system**	orthorhombic	monoclinic	monoclinic	monoclinic
**Space group**	*P*bca	$P2_1/c$	$P2_1/n$	$P2_1/c$
**a/Å**	19.5151(5)	26.3787(7)	13.6279(5)	8.0186(2)
**b/Å**	7.64427(16)	10.8773(3)	11.1304(3)	10.5772(3)
**c/Å**	29.7943(7)	19.2009(5)	17.4022(5)	29.8312(10)
**α/°**	90	90	90	90
**β/°**	90	97.435(2)	104.415(3)	92.182(3)
**γ/°**	90	90	90	90
**Volume/Å^3**	4444.67(18)	5463.0(3)	2556.51(14)	2528.27(13)
**Z**	8	8	4	4
**ρ_{calc}g/cm^3**	1.718	1.532	1.600	1.660
**μ/mm^{-1}**	12.153	1.208	1.289	1.308
**F(000)**	2312.0	2571.4	1256.0	1288.0
**Crystal size/mm^3**	0.15 × 0.12 × 0.06	0.23 × 0.09 × 0.02	0.23 × 0.17 × 0.08	0.56 × 0.35 × 0.14
**Radiation**	CuKα (λ = 1.54184)	MoKα (λ = 0.71073)	MoKα (λ = 0.71073)	MoKα (λ = 0.71073)
**2Θ range for data collection/°**	5.932 to 148.392	4.06 to 59.54	6.234 to 59.494	5.626 to 59.45
**Index ranges**	-24 ≤ h ≤ 22, -9 ≤ k ≤ 7, -34 ≤ l ≤ 37	-35 ≤ h ≤ 36, -15 ≤ k ≤ 14, -26 ≤ l ≤ 23	-13 ≤ h ≤ 19, -12 ≤ k ≤ 14, -23 ≤ l ≤ 23	-10 ≤ h ≤ 11, -14 ≤ k ≤ 11, -31 ≤ l ≤ 39
**Reflections collected**	21612	53492	18472	15950
**Independent reflections**	4521 [R_{int} = 0.0895, R_{sigma} = 0.0567]	13805 [R_{int} = 0.0469, R_{sigma} = 0.0522]	6330 [R_{int} = 0.0736, R_{sigma} = 0.0859]	6140 [R_{int} = 0.0642, R_{sigma} = 0.0877]
**Data/restraints/parameters**	4521/0/377	13805/0/659	6330/0/421	6140/0/376
**Goodness-of-fit on F^2**	1.056	1.093	1.112	1.053
**Final R indexes [I>=2σ (I)]**	R_1 = 0.0586, wR_2 = 0.1327	R_1 = 0.0442, wR_2 = 0.0761	R_1 = 0.0454, wR_2 = 0.0853	R_1 = 0.0524, wR_2 = 0.0940
**Final R indexes [all data]**	R_1 = 0.0837, wR_2 = 0.1483	R_1 = 0.0624, wR_2 = 0.0820	R_1 = 0.0646, wR_2 = 0.1017	R_1 = 0.0853, wR_2 = 0.1108
**Largest diff. peak/hole / e Å^{-3}**	1.41/-1.21	1.11/-0.82	1.01/-0.83	0.93/-0.72

Table S 7: X-ray crystallographic data for compounds 12-15, s.u.s in parenthesis.

Compound	12	13	14	15
**CSD Number**	**2055568**	**2055569**	**2055574**	**2203163**
**Empirical formula**	$C_{30}H_{31}ClFeO_3Ru$	$C_{31}H_{33}ClFeO_3Ru$	$C_{29}H_{28}ClFFeO_2Ru$	$C_{29}H_{27}ClFFeO_2Ru$
**Formula weight**	631.92	645.94	619.88	618.87
**Temperature/K**	120.02(10)	119.99(11)	119.99(13)	119.98(18)
**Crystal system**	monoclinic	monoclinic	monoclinic	monoclinic
**Space group**	$P2_1/n$	$P2_1/n$	$P2_1/c$	$P2_1/c$
**a/Å**	14.1003(4)	8.1197(3)	9.8244(2)	12.5423(3)
**b/Å**	11.0082(2)	7.4226(3)	24.0394(5)	10.900(2)
**c/Å**	17.4375(5)	44.6546(13)	10.9713(3)	19.296(4)
**α/°**	90	90	90	90
**β/°**	105.601(3)	91.927(3)	101.158(3)	97.40(3)
**γ/°**	90	90	90	90
**Volume/Å^3**	2606.92(11)	2689.79(17)	2542.14(11)	2615.8(9)
**Z**	4	4	4	4
**ρ_{calc}g/cm^3**	1.610	1.595	1.620	1.571
**μ/mm^{-1}**	1.269	1.232	10.616	1.265
**F(000)**	1288.0	1320.0	1256.0	1252.0
**Crystal size/mm^3**	0.25 × 0.16 × 0.15	0.52 × 0.15 × 0.11	0.09 × 0.07 × 0.05	0.16 × 0.13 × 0.05
**Radiation**	MoKα (λ = 0.71073)	MoKα (λ = 0.71073)	CuKα (λ = 1.54184)	MoKα (λ = 0.71073)
**2Θ range for data collection/°**	6.198 to 59.65	5.798 to 59.516	7.354 to 148.192	6.264 to 52.042
**Index ranges**	-16 ≤ h ≤ 17, -15 ≤ k ≤ 11, -21 ≤ l ≤ 24	-10 ≤ h ≤ 11, -7 ≤ k ≤ 10, -57 ≤ l ≤ 60	-12 ≤ h ≤ 12, -26 ≤ k ≤ 29, -13 ≤ l ≤ 12	-15 ≤ h ≤ 13, -13 ≤ k ≤ 13, -23 ≤ l ≤ 23
**Reflections collected**	18767	19150	18908	18397
**Independent reflections**	6397 [R_{int} = 0.0750, R_{sigma} = 0.0782]	6463 [R_{int} = 0.0494, R_{sigma} = 0.0597]	5062 [R_{int} = 0.0808, R_{sigma} = 0.0649]	5146 [R_{int} = 0.0672, R_{sigma} = 0.0884]
**Data/restraints/parameters**	6397/0/438	6463/0/466	5062/0/424	5146/0/329
**Goodness-of-fit on F^2**	1.085	1.083	1.034	1.148
**Final R indexes [I>=2σ (I)]**	R_1 = 0.0452, wR_2 = 0.0974	R_1 = 0.0400, wR_2 = 0.0713	R_1 = 0.0427, wR_2 = 0.0956	R_1 = 0.0682, wR_2 = 0.1369
**Final R indexes [all data]**	R_1 = 0.0565, wR_2 = 0.1094	R_1 = 0.0520, wR_2 = 0.0773	R_1 = 0.0620, wR_2 = 0.1068	R_1 = 0.0893, wR_2 = 0.1450
**Largest diff. peak/hole / e Å^{-3}**	0.92/-0.82	0.49/-0.65	1.13/-0.75	2.45/-0.77

Table S 8: X-ray crystallographic data for compounds 16-18, s.u.s in parenthesis.

Compound	16	17	18
**CSD Number**	**2055582**	**2055580**	**2055575**
**Empirical formula**	$C_{29}H_{25}ClF_2FeO_2Ru$	$C_{29}H_{28}ClFFeO_2Ru$	$C_{29}H_{28}Cl_2FeO_2Ru$
**Formula weight**	635.86	619.88	636.359
**Temperature/K**	120.00(10)	120.02(18)	290.42(10)
**Crystal system**	triclinic	monoclinic	monoclinic
**Space group**	*P*-1	*I*2/a	*C*2/c
**a/Å**	7.6335(3)	27.2039(9)	27.5007(7)
**b/Å**	12.2511(6)	10.8230(3)	11.1178(2)
**c/Å**	13.9983(8)	19.3918(6)	19.0626(4)
**α/°**	73.786(5)	90	90
**β/°**	82.165(4)	102.324(3)	102.852(2)
**γ/°**	89.830(4)	90	90
**Volume/Å³**	1244.45(11)	5577.9(3)	5682.3(2)
**Z**	2	8	8
**ρ_{calc}g/cm³**	1.697	1.476	1.488
**μ/mm⁻¹**	1.338	1.187	1.253
**F(000)**	640.0	2507.1	2572.2
**Crystal size/mm³**	0.14 × 0.11 × 0.06	0.41 × 0.09 × 0.06	0.20 × 0.18 × 0.09
**Radiation**	MoKα (λ = 0.71073)	MoKα (λ = 0.71073)	Mo Kα (λ = 0.71073)
**2Θ range for data collection/°**	6.124 to 59.542	5.568 to 59.584	5.66 to 59.62
**Index ranges**	-10 ≤ h ≤ 8, -16 ≤ k ≤ 16, -19 ≤ l ≤ 17	-37 ≤ h ≤ 35, -15 ≤ k ≤ 13, -25 ≤ l ≤ 25	-38 ≤ h ≤ 30, -14 ≤ k ≤ 15, -25 ≤ l ≤ 26
**Reflections collected**	15001	32091	24276
**Independent reflections**	6057 [R_{int} = 0.0497, R_{sigma} = 0.0708]	7232 [R_{int} = 0.0466, R_{sigma} = 0.0449]	7102 [R_{int} = 0.0357, R_{sigma} = 0.0406]
**Data/restraints/parameters**	6057/0/403	7232/0/420	7102/0/391
**Goodness-of-fit on F²**	1.043	1.010	1.130
**Final R indexes [I>=2σ (I)]**	R_1 = 0.0437, wR_2 = 0.0824	R_1 = 0.0332, wR_2 = 0.0621	R_1 = 0.0420, wR_2 = 0.0819
**Final R indexes [all data]**	R_1 = 0.0579, wR_2 = 0.0923	R_1 = 0.0480, wR_2 = 0.0661	R_1 = 0.0556, wR_2 = 0.0870
**Largest diff. peak/hole / e Å⁻³**	0.86/-0.72	0.51/-0.47	0.87/-0.70

Table S 9: X-ray crystallographic data for compounds 19-21, s.u.s in parenthesis.

Compound	19	20	21
**CSD Number**	**2055579**	**2055576**	**2203164**
**Empirical formula**	$C_{29}H_{27}Cl_3FeO_2Ru$	$C_{29}H_{28}Cl_2FeO_2Ru$	$C_{29}H_{28}BrClFeO_2Ru$
**Formula weight**	670.77	636.33	680.79
**Temperature/K**	290.42(10)	120.00(10)	120.1(3)
**Crystal system**	monoclinic	monoclinic	monoclinic
**Space group**	$P2_1$/c	$P2_1$/n	$C2$/c
**a/Å**	26.1659(4)	13.6289(6)	27.732(6)
**b/Å**	10.8628(15)	11.1257(4)	11.143(3)
**c/Å**	19.1484(2)	17.3444(8)	19.031(4)
**α/°**	90	90	90
**β/°**	96.419(12)	104.663(5)	102.77(2)
**γ/°**	90	90	90
**Volume/Å^3**	5408.54(13)	2544.3(2)	5735.0(2)
**Z**	8	4	8
**ρ_{calc}g/cm^3**	1.648	1.661	1.577
**μ/mm^{-1}**	1.417	1.400	2.542
**F(000)**	2704.0	1228.0	2720.0
**Crystal size/mm^3**	0.16 × 0.15 × 0.06	0.34 × 0.31 × 0.25	0.14 × 0.1 × 0.04
**Radiation**	MoKα (λ = 0.71073)	MoKα (λ = 0.71073)	Mo Kα (λ = 0.71073)
**2Θ range for data collection/°**	4.064 to 59.626	6.826 to 59.672	5.656 to 52.042
**Index ranges**	-32 ≤ h ≤ 36, -14 ≤ k ≤ 13, -26 ≤ l ≤ 26	-15 ≤ h ≤ 19, -14 ≤ k ≤ 15, -24 ≤ l ≤ 23	-34 ≤ h ≤ 33, -13 ≤ k ≤ 10, -21 ≤ l ≤ 23
**Reflections collected**	49369	17784	23472
**Independent reflections**	13528 [R_{int} = 0.0415, R_{sigma} = 0.0486]	6283 [R_{int} = 0.0761, R_{sigma} = 0.0777]	5651 [R_{int} = 0.0395, R_{sigma} = 0.0343]
**Data/restraints/parameters**	13528/0/655	6283/0/319	5651/0/319
**Goodness-of-fit on F^2**	1.065	1.094	1.184
**Final R indexes [I>=2σ (I)]**	R_1 = 0.0351, wR_2 = 0.0602	R_1 = 0.0460, wR_2 = 0.1013	R_1 = 0.0512, wR_2 = 0.1096
**Final R indexes [all data]**	R_1 = 0.0614, wR_2 = 0.0705	R_1 = 0.0571, wR_2 = 0.1132	R_1 = 0.0577, wR_2 = 0.1120
**Largest diff. peak/hole / e Å^{-3}**	0.59/-0.56	0.89/-0.71	1.04/-1.39

Table S 10: X-ray crystallographic data for compounds 22-24, s.u.s in parenthesis.

Compound	22	23	24
**CSD Number**	**2055578**	**2055581**	**2055583**
**Empirical formula**	$C_{29}H_{28}BrClFeO_2Ru$	C29H28ClFeIO2Ru	$C_{29}H_{28}ClFeIO_2Ru$
**Formula weight**	680.79	727.78	727.78
**Temperature/K**	120.00(10)	120.1(4)	119.99(18)
**Crystal system**	monoclinic	monoclinic	monoclinic
**Space group**	$P2_1$/n	*I*2/a	$P2_1$/n
**a/Å**	13.7359(7)	20.2239(5)	13.9939(7)
**b/Å**	11.0897(5)	7.7724(2)	11.0719(5)
**c/Å**	17.3928(10)	33.7981(13)	17.4029(8)
**α/°**	90	90	90
**β/°**	105.015(6)	91.683(3)	105.470(5)
**γ/°**	90	90	90
**Volume/Å³**	2559.0(2)	5310.4(3)	2598.7(2)
**Z**	4	8	4
**ρ_{calc}g/cm³**	1.767	1.821	1.860
**μ/mm^{-1}**	2.849	19.234	2.456
**F(000)**	1360.0	2864.0	1432.0
**Crystal size/mm³**	0.2 × 0.15 × 0.03	0.14 × 0.05 × 0.05	0.16 × 0.11 × 0.03
**Radiation**	MoKα (λ = 0.71073)	CuKα (λ = 1.54184)	MoKα (λ = 0.71073)
**2Θ range for data collection/°**	5.716 to 62.508	8.748 to 147.438	6.2 to 59.482
**Index ranges**	-19 ≤ h ≤ 19, -13 ≤ k ≤ 15, -24 ≤ l ≤ 20	-24 ≤ h ≤ 25, -9 ≤ k ≤ 7, -38 ≤ l ≤ 39	-18 ≤ h ≤ 19, -13 ≤ k ≤ 14, -24 ≤ l ≤ 24
**Reflections collected**	20802	10576	17649
**Independent reflections**	7337 [R_{int} = 0.0636, R_{sigma} = 0.0840]	4947 [Rint = 0.0395, Rsigma = 0.0498]	6256 [R_{int} = 0.0546, R_{sigma} = 0.0747]
**Data/restraints/parameters**	7337/0/383	4947/0/347	6256/0/376
**Goodness-of-fit on F²**	1.027	1.050	1.067
**Final R indexes [I>=2σ (I)]**	R_1 = 0.0475, wR_2 = 0.0755	R_1 = 0.0420, wR_2 = 0.1016	R_1 = 0.0470, wR_2 = 0.0750
**Final R indexes [all data]**	R_1 = 0.0744, wR_2 = 0.0861	R_1 = 0.0540, wR_2 = 0.1087	R_1 = 0.0710, wR_2 = 0.0825
**Largest diff. peak/hole / e Å^{-3}**	0.70/-0.95	1.51/-1.02	1.46/-0.83

Table S 11: Selected bond lengths (Å) for compounds 1, 2, 4-6 and 9-24, s.u.s are shown in parentheses.

Compound	Ru1-O1	Ru1-O2	Ru1-Cl1
**1**	2.057(3)/	2.078(3)/	2.4178(10)/
	2.074(3)	2.077(3)	2.4158(10)
**2**	2.066(2)	2.071(2)	2.4137(8)
**4**	2.067(3)	2.066(3)	2.4186(10)
**5**	2.0800(17)	2.0784(17)	2.4190(6)
**6**	2.082(4)	2.075(4)	2.4163(17)
**9**	2.0722(19)/ 2.058(2)	2.0708(18)/ 2.0734(19)	2.4078(7)/ 2.4141(7)
**10**	2.078(2)	2.077(2)	2.4151(8)
**11**	2.086(3)	2.072(3)	2.4232(10)
**12**	2.075(2)	2.0810(19)	2.4098(8)
**13**	2.0558(18)	2.0696(17)	2.4197(7)
**14**	2.081(3)	2.076(3)	2.4131(11)
**15**	2.077(4)	2.081(4)	2.4089(15)
**16**	2.071(2)	2.065(2)	2.4255(9)
**17**	2.0815(14)	2.0836(15)	2.4089(5)
**18**	2.083(2)	2.068(2)	2.4066(8)
**19**	2.0760(18)/ 2.0621(18)	2.0772(17)/ 2.0793(18)	2.4063(6)/ 2.4117(6)
**20**	2.085(2)	2.072(2)	2.4110(8)
**21**	2.086(4)	2.079(4)	2.4061(14)
**22**	2.079(2)	2.071(2)	2.4130(9)
**23**	2.082(4)	2.063(4)	2.4193(13)
**24**	2.079(3)	2.068(3)	2.4115(10)

Table S 12: Selected bond angles (°) for compounds 1-2, 4-6, and 9-24, s.u.s are stated in parenthesis.

Compound	O1-Ru1-O2	O1-Ru1-Cl1	O2-Ru1-Cl1
**1**	87.81(11)/87.18(11)	85.45(8)/ 84.70(8)	85.18(8)/ 86.40(8)
**2**	88.05(9)	86.37(6)	84.83(6)
**4**	88.38(10)	84.39(8)	85.58(8)
**5**	87.23(7)	86.46(5)	86.08(5)
**6**	88.66(17)	85.50(13)	85.49(14)
**9**	86.82(7)/ 87.54(8)	86.52(6)/ 86.27(6)	85.44(6)/ 85.54(8)
**10**	86.73(9)	86.64(6)	85.88(6)
**11**	86.94(11)	87.15(8)	86.23(8)
**12**	87.22(6)	87.41(6)	84.84(6)
**13**	88.86(7)	84.04(5)	86.15(6)
**14**	89.03(12)	84.26(9)	86.33(9)
**15**	87.14(15)	86.59(11)	85.50(12)
**16**	86.96(9)	85.05(6)	85.39(7)
**17**	87.74(6)	86.81(4)	84.71(4)
**18**	86.74(9)	86.91(6)	85.45(6)
**19**	86.36(7)/ 87.26(7)	86.95(5)/ 86.39(5)	85.31(5)/ 85.66(5)
**20**	86.97(8)	86.92(6)	85.59(6)
**21**	86.94(14)	86.99(10)	85.16(11)
**22**	86.81(8)	86.87(6)	85.53(7)
**23**	88.19(16)	85.34(11)	83.59(12)
**24**	87.03(11)	86.88(8)	85.29(8)

NMR Ligand and Complex

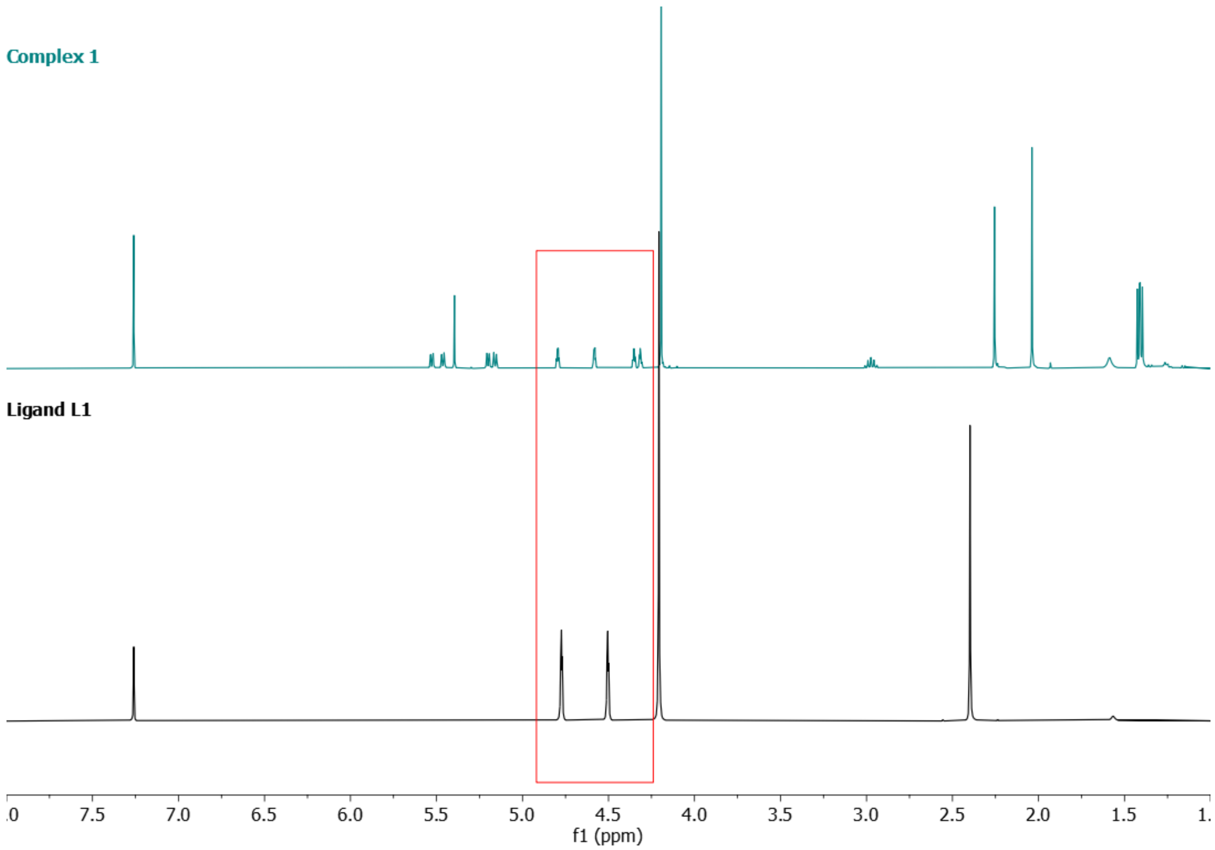

Figure S 8: Ligand L1 and complex 1 – showing the loss in symmetry of the top ferrocenyl Cp ring on complexation to the Ru(II) center ($CDCl_3$, 400 MHz).

Chemosensitivity Studies

To deduce any structural-activity relationships (SARs) of the complexes, chemosensitivity studies were performed for complexes **1-24**, cisplatin (**CDDP**), carboplatin (**CARB**) and oxaliplatin (**OXA**), using a 96 h MTT assay (**Figures 3** and **Table S13**). All compounds were screened against human pancreatic carcinoma (MIA PaCa-2) and human colorectal adenocarcinoma *p53*-wildtype (HCT116 $p53^{+/+}$). The results show that complexes display varying cytotoxic towards both cancer cells lines tested, with a general increase in activity observed against MIA PaCa-2. The 2-furan Fc-acac functionalized complex **4**, exhibited the highest cytotoxicity (IC_{50} = 8 ± 2 µM, *cf.* CDDP = 3.6 ± 0.7 µM) against MIA PaCa-2. Interestingly, the 3-furan Fc-acac functionalized complex **5** (IC_{50} = 40 ± 9 µM; MIA PaCa-2), exhibits a 5-fold decrease in activity when compared to **4**. Though **4** exhibits the highest IC_{50} value against MIA PaCa-2, it has a significantly lower potency than the analogues reported bis(bipyridine)ruthenium ferrocenyl β-diketonato complex (e.g. **Figure 1F**), which has an IC_{50} value of 0.11 ± 0.01 µM.[8]

When functionalizing the methyl Fc-acac complex (**1**) (IC_{50} = 92-93 µM), with a phenyl substituent (**2**), the activity increases by ~2-fold against MIA PaCa-2 (IC_{50} = 50 ± 6 µM). On increasing the hydrophobicity further from Ph (**2**) to 1-Np (**3**), the IC_{50} values decrease and complex **3** is inactive against all cell lines (IC_{50} > 100 µM), which shows IC_{50} values do not correlate with hydrophobicity. The hydrophobicity of each complex has been obtained from an octanol-water shake-flask method and the data is shown in (**Table S14**). The only examples without a functionalized furan or phenyl ring are complexes **1** (-Me), **6** (-CHF_2), and **7** (-CF_3), and replacing the methyl group (**1**) for -CHF_2 (**6**), decreases the cytotoxicity further (**Figure S8**, IC_{50} >100 µM; MIA PaCa-2). Changing the functional group from -CHF_2 (**6**) to –CF_3 (**7**) did not increase the complexes cytotoxicity (IC_{50} > 100 [illegible]M). However, when comparing complexes **6** and **7** against HCT116 $p53^{+/+}$, the cytotoxicity increases by ~2-fold for complex **7**

(IC_{50} values = 95 ± 9 µM (**6**) and 51 ± 4 µM (**7**)). It should be noted that when comparing to the recent work of Manikandan et al., complexes **1** (CH_3) and **7** (CF_3) follow the same trend,[10] whereby the activity of **7** > **1**, however, their activities are significantly lower than what has been reported against HeLa (cervical), A2780 (ovarian) and A2780cisR (cisplatin resistant ovarian). This highlights potential selectivity towards these cell lines and highlights the need for further screening of our library against a wider range of cancerous cells.

When comparing the IC_{50} values of the mono-*meta* (3') and di-*meta* (3',5') substituted compounds (**Figure S9**). In the case of the methyl complexes **8** (3'-Me) and **9** (3',5'-diMe), and chloro complexes **18** (3'-Cl) and **19** (3',5'-diCl), the di-*meta* substituted compounds (**9** and **19**) are more cytotoxic than the mono-*meta* substituted compounds (**8** and **18**) against MIA PaCa-2. Complexes **9** and **19** are ~4x more cytotoxic than the both mono-*meta* complexes **8** and **18** (**Figure S10**). Upon comparison of the compounds substituted in the *meta* and *para* positions, the cytotoxicity of the methyl, fluoro and chloro compounds increase when substituted in the *para* position (**Figure S11**). The most significant increase in potency is observed for the fluoro complexes **15** (3'-F) *cf.* **17** (4'-F) and chloro complexes **18** (3'-Cl) *cf.* **20** (4'-Cl) when tested against MIA PaCa-2, whereby the cytotoxicity increases by >3-fold for the *para* substituted compounds (**Figure S12**). The same trend is not observed when tested against the HCT116 $p53^{+/+}$ cell line, and the cytotoxicity generally decreases when comparing substitution in the *meta* and *para* position.

Table S 13: IC_{50} values (µM ± SD) for complexes 1-24 against, cisplatin (CDDP), carboplatin (CARB) and oxaliplatin (OXA) against MIA-PaCa-2, HCT116 $p53^{+/+}$ and ARPE-19 cell lines after 96 h incubation. Selectivity index (SI) values shown in parenthesis.

Compound	MIA PaCa-2	HCT116 $p53^{+/+}$	ARPE-19
**1**	93 ± 7 (1.1*)	87.6 ± 0.3 (1.1*)	>100
**2**	50 ± 4 (2.0*)	84 ± 5 (1.2*)	>100
**3**	>100 (n.d.)	>100 (n.d.)	>100
**4**	8 ± 1 (12.7*)	72 ± 11 (1.4*)	>100
**5**	40 ± 5 (2.5*)	75 ± 6 (1.3*)	>100
**6**	>100 (n.d.)	95 ± 5 (1.1*)	>100
**7**	11 ± 1 (8.8*)	51 ± 2 (2.0*)	>100
**8**	>100 (n.d.)	84± 8 (1.2*)	>100
**9**	25 ± 2 (4.1*)	52 ± 7 (1.9*)	>100
**10**	75 ± 1 (1.3*)	>100 (n.d.)	>100
**11**	40 ± 6 (2.5*)	88 ± 10 (1.1*)	>100
**12**	65 ± 12 (1.5*)	>100 (n.d.)	>100
**13**	39 ± 5 (2.6*)	48 ± 5 (2.1*)	>100
**14**	>100 (n.d.)	89.4 ± 0.3 (1.1*)	>100
**15**	26 ± 1 (3.8*)	78 ± 4 (1.3*)	>100
**16**	>100 (n.d.)	76 ± 7 (1.3*)	>100
**17**	33 ± 2 (3.1*)	76 ± 7 (1.2*)	>100
**18**	>100 (n.d.)	75 ± 11 (1.3*)	>100
**19**	26 ± 5 (3.8*)	53 ± 9 (1.9*)	>100
**20**	29 ± 2 (3.5*)	78 ± 10 (1.3*)	>100
**21**	>100 (n.d.)	65 ± 11 (1.5*)	>100
**22**	>100 (n.d.)	>100 (n.d.)	>100
**23**	25 ± 4 (4.0*)	>100 (n.d.)	>100
**24**	>100 (n.d.)	>100 (n.d.)	>100
**CDDP**	3.6 ± 0.7 (1.7)	3.3 ± 0.4 (1.8)	6 ± 1
**CARB**	36 ± 3 (2.2)	32 ± 11 (2.4)	77 ± 10
**OXA**	6 ± 1 (0.5)	0.9 ± 0.1 (3.2)	3.0 ± 0.3

n.d. defines not determined

*** defines the minimum values, as at least one IC_{50} is > 100 µM**

Hydrophobicity versus Cytotoxicity

Table S 14: Experimental hydrophobicity values highlighting the most hydrophobic (red) and hydrophilic (blue) complex (excluding CDDP), alongside their cytotoxicity against MIA PaCa-2 (μM ± SD) after 96 h.

Compound	LogP	IC_{50} values (μM) ± SD	Compound	LogP	IC_{50} values (μM) ± SD
**1**	1.11 ± 0.09	93 ± 7	**14**	1.36 ± 0.16	>100
**2**	0.8 ± 0.1	50 ± 4	**15**	1.26 ± 0.05	26 ± 1
**3**	1.04 ± 0.2	>100	**16**	0.74 ± 0.05	>100
**4**	1.02 ± 0.11	8 ± 1	**17**	0.79 ± 0.09	33 ± 2
**5**	0.97 ± 0.1	40 ± 6	**18**	0.42 ± 0.19	>100
**6**	1.13 ± 0.09	>100	**19**	1.86 ± 0.22	26 ± 5
**7**	1.76 ± 0.18	11 ± 2	**20**	0.69 ± 0.06	29 ± 2
**8**	0.64 ± 0.22	>100	**21**	1.09 ± 0.23	>100
**9**	0.55 ± 0.13	25 ± 2	**22**	1.55 ± 0.45	>100
**10**	0.33 ± 0.08	75 ± 1	**23**	1.17 ± 0.17	25 ± 4
**11**	1.22 ± 0.04	40 ± 6	**24**	1.40 ± 0.16	>100
**12**	1.08 ± 0.01	65 ± 12	**CDDP**	-2.20 ± 0.20	3.6 ± 0.7
**13**	1.22 ± 0.04	39 ± 5			

Table S 15: IC_{50} values (μM ± SD) for ligands L1, L2, L4 L7 and complexes 1, 2, 4, 7 and cisplatin (CDDP) against MIA PaCa-2 after 24 h and 48 h.

Compound	IC_{50} values ± SD		
	**24 h**	**48 h**	**96 h**
**L1**	76 ± 3	61 ± 1	59 ± 2
**1**	>100	>100	93 ± 7
**L2**	>100	68 ± 3	38.7 ± 0.8
**2**	>100	>100	50 ± 4
**L4**	>100	43 ± 1	42.1 ± 0.8
**4**	>100	23 ± 2	8 ± 1
**L7**	>100	20 ± 1	17 ± 1
**7**	>100	30 ± 1	11 ± 2
**CDDP**	>100	76 ± 3	3.6 ± 0.7

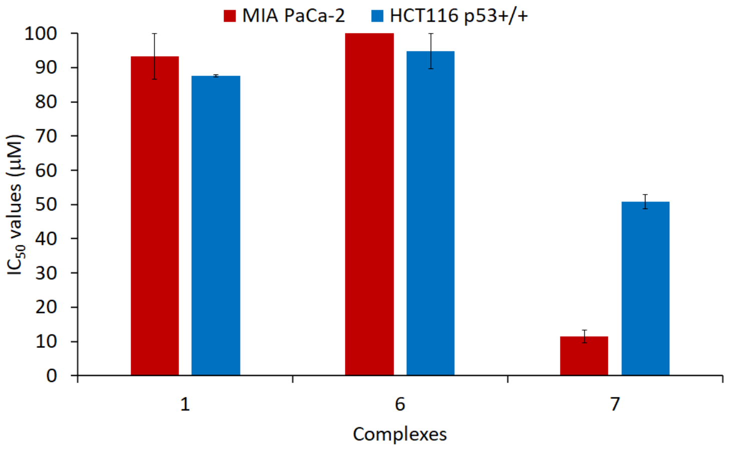

Figure S 9: IC_{50} values of complexes 1, 6 and 7 when tested against MIA PaCa-2 and HCT116 $p53^{+/+}$.

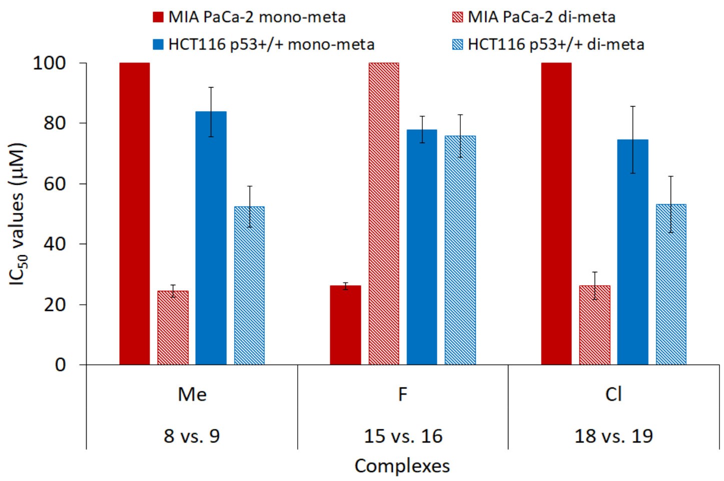

Figure S 10: IC_{50} values (µM) of mono-meta and di-meta substituted compounds when tested against MIA PaCa-2 and HCT116 $p53^{+/+}$.

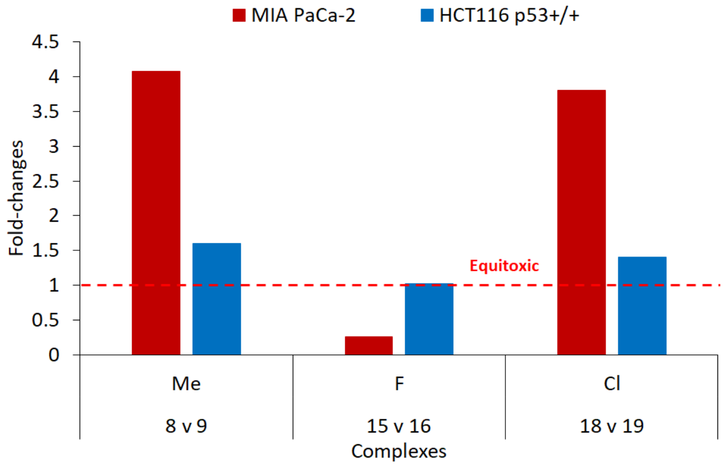

Figure S 11: Fold-changes of the mono-meta versus di-meta substituted compounds when tested against MIA PaCa-2 and HCT116 $p53^{+/+}$.

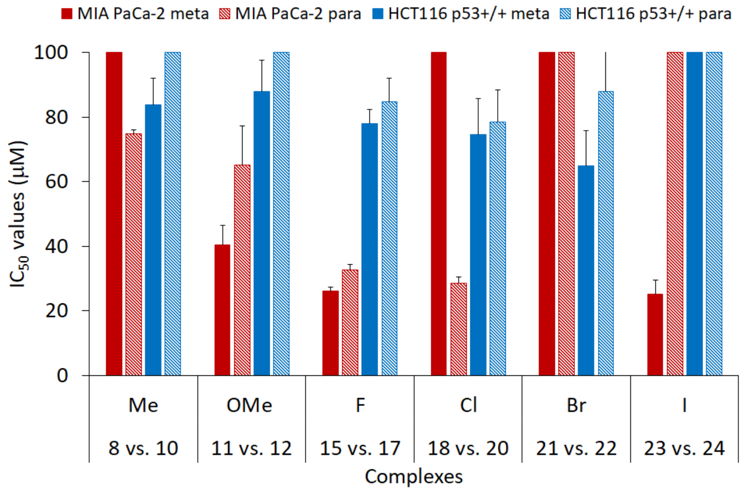

Figure S 12: IC_{50} values (µM) of meta and para substituted compounds when tested against MIA PaCa-2 and HCT116 $p53^{+/+}$.

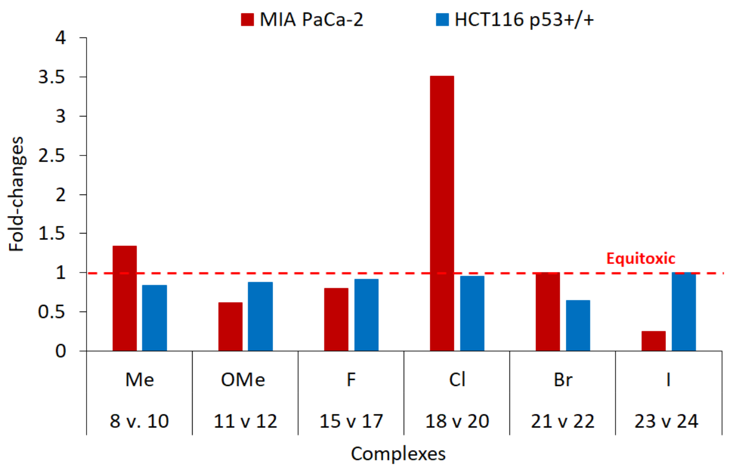

Figure S 13: Fold-changes of the mono-meta versus di-meta substituted compounds when tested against MIA PaCa-2 and HCT116 p53$^{+/+}$.

Hypoxia Studies

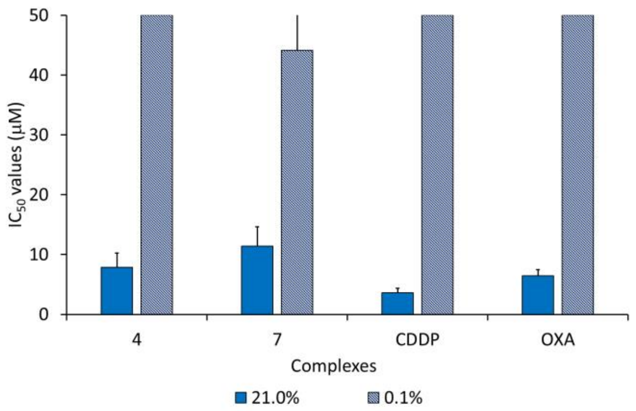

Figure S 14: IC_{50} values for complexes 4, 7, CDDP and OXA when screened against MIA PaCa-2 under normoxic (21%) and hypoxic (0.1%) conditions.

UV-vis Stability Studies

All spectra were obtained after **initial (dark blue)**, **24 h (light blue)**, **48 h (pink)**, **72 h (purple)** and **96 h (black)**. Similar trends are observed in most complexes, intense ligand-based absorbance (π-π*) can be observed at approximately 200 nm, followed by less intense metal-to-ligand charge transfer (MLCT) transitions, inter-ligand and ligand-centered transitions at 250-330 nm and a weak d-d transition at 450-550 nm; all of which experience a loss in intensity over the 4-day period.

Table S 16: Wavelengths of absorption bands of compounds 1-24 after initial and day 4.

Compound	Wavelength / nm	
	**Day 0**	**Day 4**
**1**	245(sh), 293, 348, 463	296, 348, 455
**2**	260, 309, 368, 489	264, 316(sh), 347
**3**	252, 308(sh), 318, 378(sh), 487	277(sh), 285, 347
**4**	274, 324, 387(sh), 492	221, 273, 347
**5**	255(sh), 310, 368, 487	274, 315(sh), 348
**6**	330, 373(sh)	345
**7**	337, 372(sh)	238(sh), 331
**8**	263, 310, 384(sh), 490	223(sh), 272, 347
**9**	266, 310, 368, 492	271, 347
**10**	267, 312, 377, 486	223, 266, 348
**11**	262, 309, 384(sh), 491	274, 347
**12**	278, 320, 374, 485	247(sh), 273(sh), 343
**13**	281, 321, 375, 483	245(sh), 273(sh), 346
**14**	256, 307, 365(sh), 495	219(sh), 271, 348
**15**	260, 311, 375(sh), 497	271, 348
**16**	263(sh), 312, 353(sh), 396(sh), 503	272, 347
**17**	261, 309, 369(sh), 494	269, 347
**18**	260, 311, 378(sh), 495	223(sh), 273, 348
**19**	261, 311, 391(sh), 505	273, 349
**20**	265, 312, 358, 497	272, 348
**21**	261, 311, 388(sh), 498	273, 351
**22**	267, 312, 385(sh), 497	269, 347
**23**	222(sh), 260, 311, 386(sh), 498	272, 347
**24**	275, 315, 383(sh), 493	243, 272(sh), 347

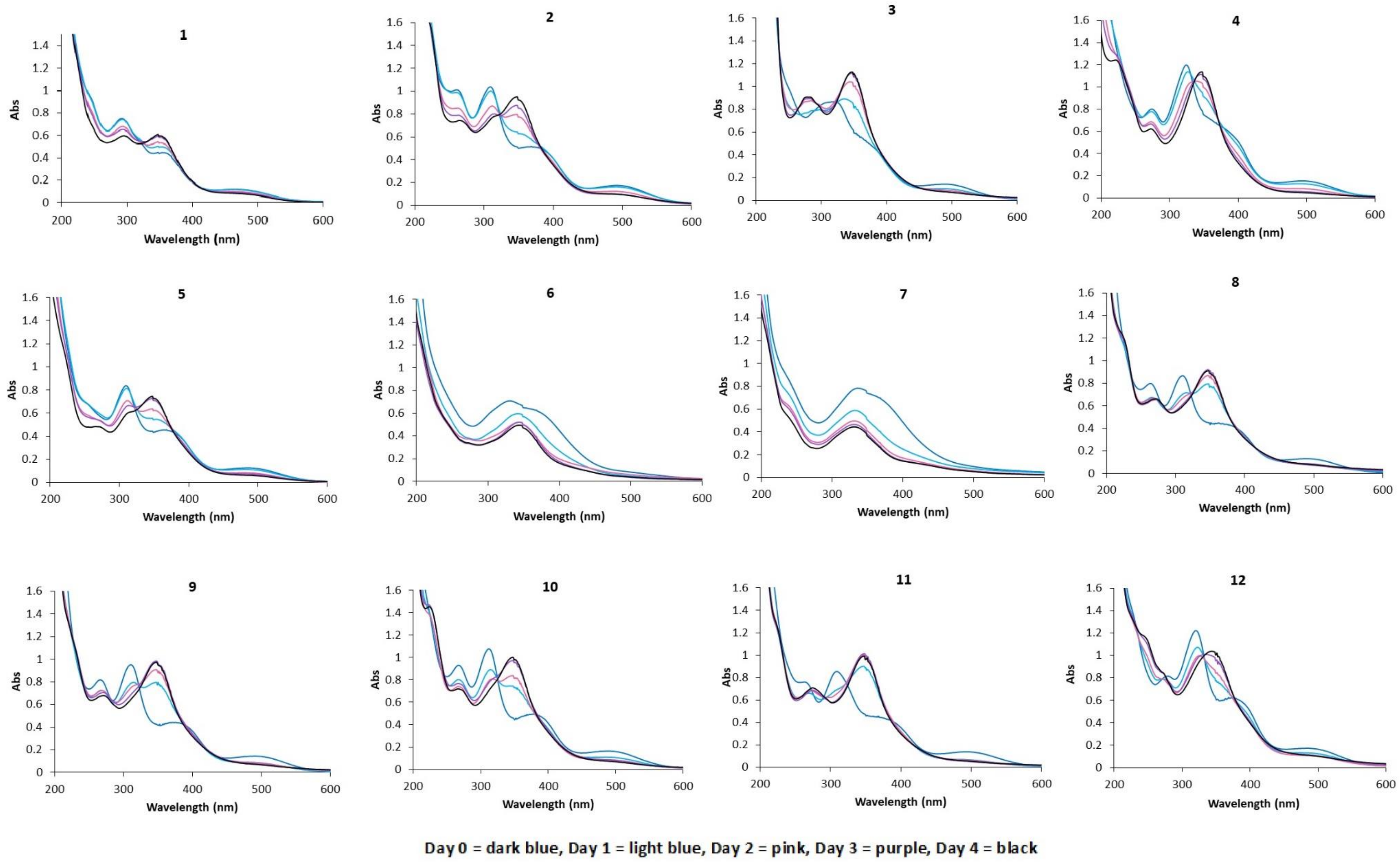

Figure S 15: UV-vis spectra for compounds 1-12 when measured after initial (dark blue), day 1 (light blue), day 2 (pink), day 3 (purple) and day 4 (black).

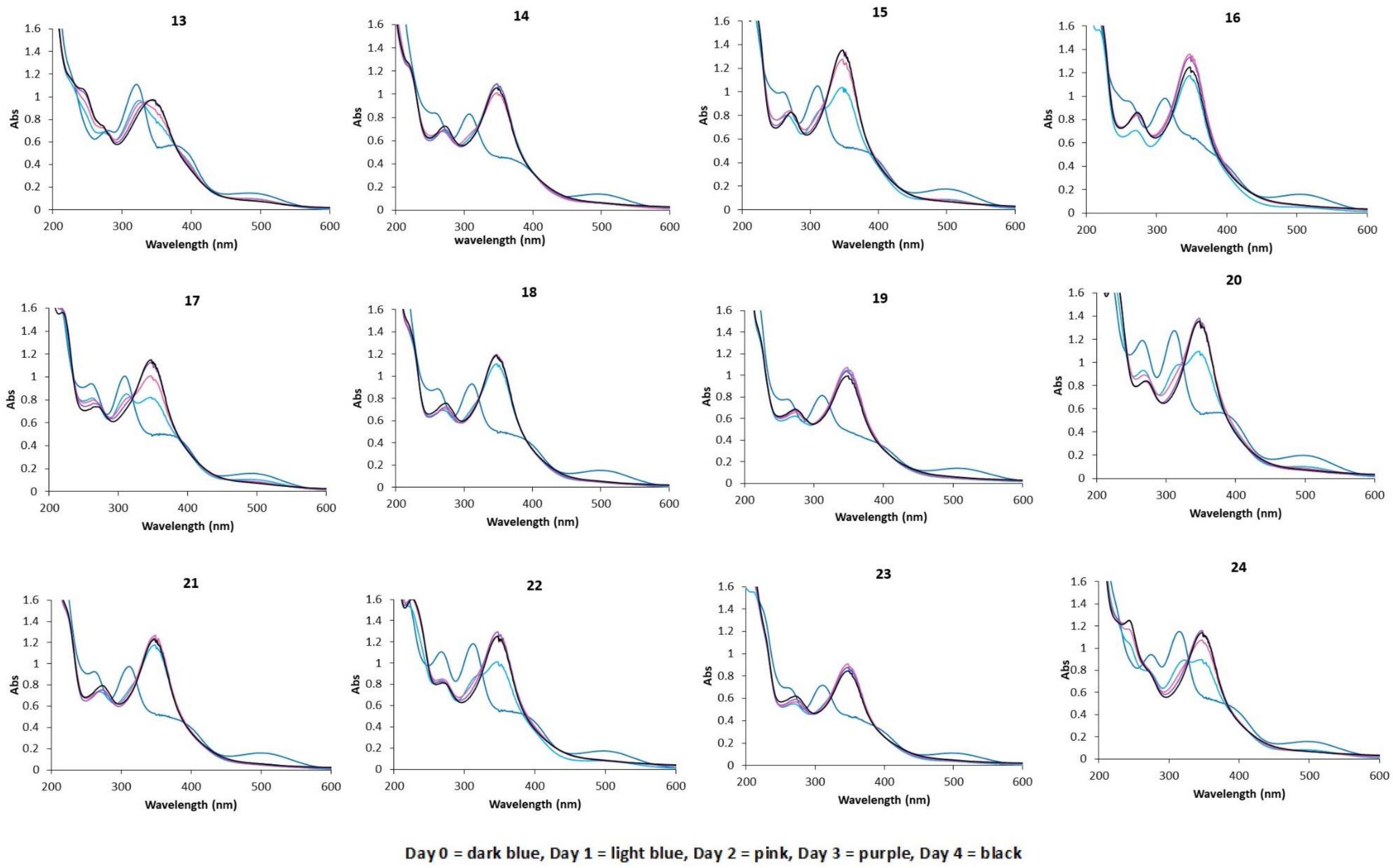

Figure S 16: UV-vis spectra for compounds 13-24 when measured after initial (dark blue), day 1 (light blue), day 2 (pink), day 3 (purple) and day 4 (black).

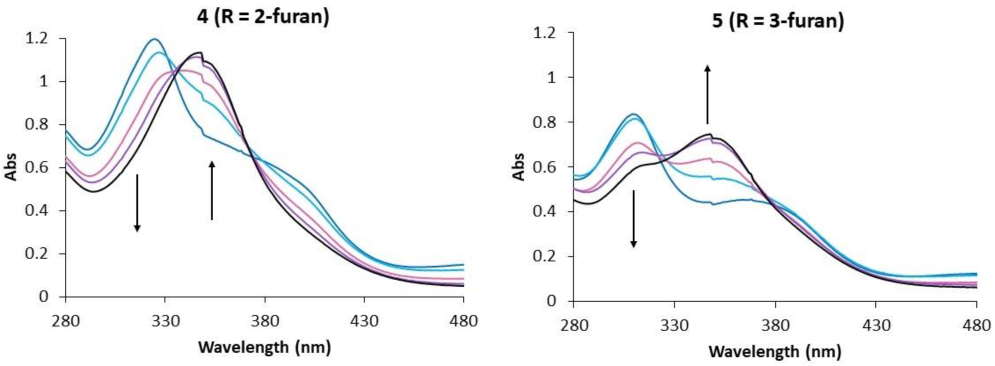

Figure S 17: UV-vis spectra for the hydrolysis of compounds 4 and 5.

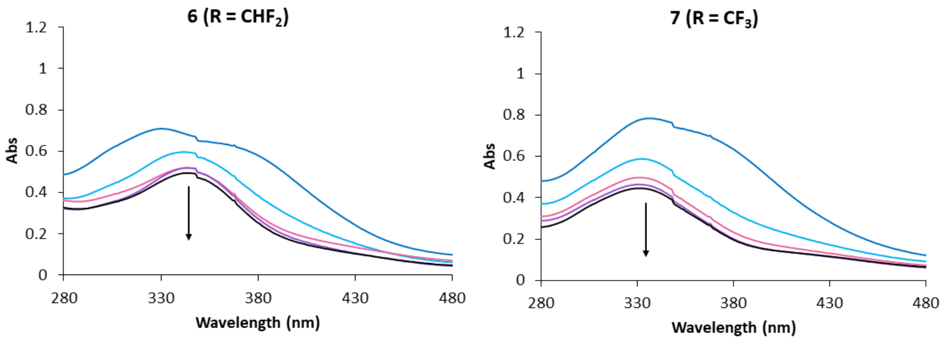

Figure S 18: UV-vis spectra for the hydrolysis of compounds 6 and 7.

NMR Stability Studies of Ferrocenyl Ligands

All spectra for ligands were obtained after **initial (black, 1)**, **20 mins (dark blue, 2)**, **40 mins (dark red, 3)**, 1 h (dark purple, 4), **2 h (dark green, 5)**, **6 h (brown, 6)**, **12 h (blue, 7)**, **24 h (red, 8)**, **48 h (purple, 9)**, **72 h (green, 10)** and **96 h (orange, 11)**.

Ligands in DMSO

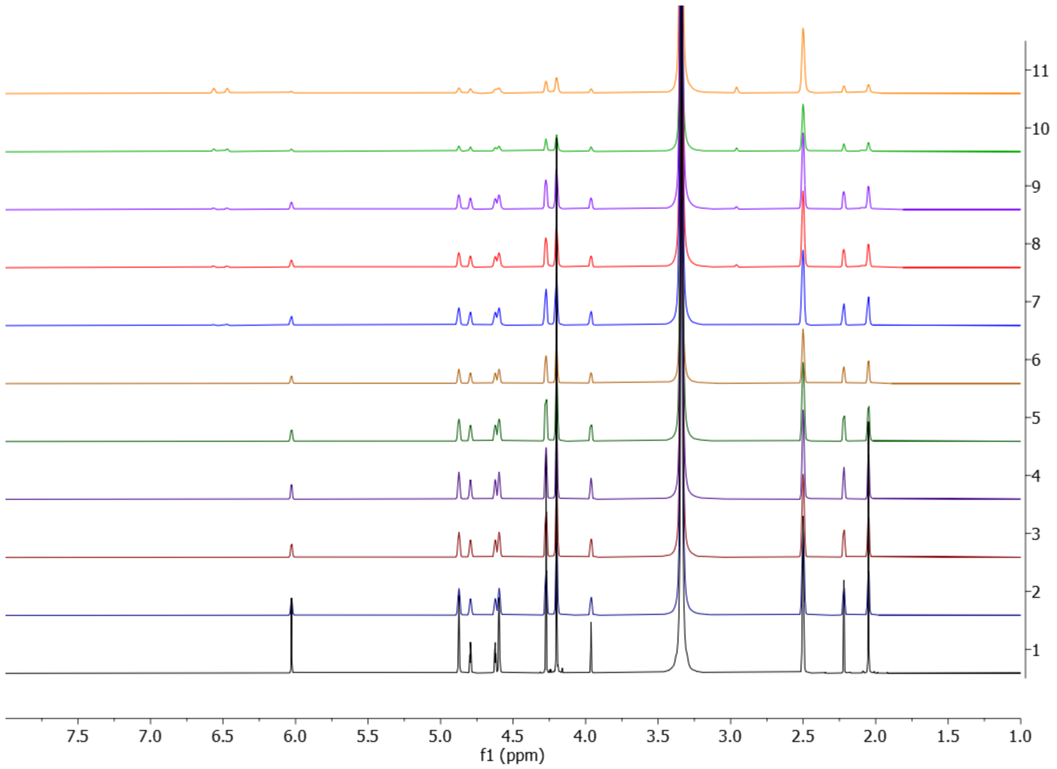

Figure S 19: Ligand L1 over 96 hours (DMSO-d$_6$, 500 MHz).

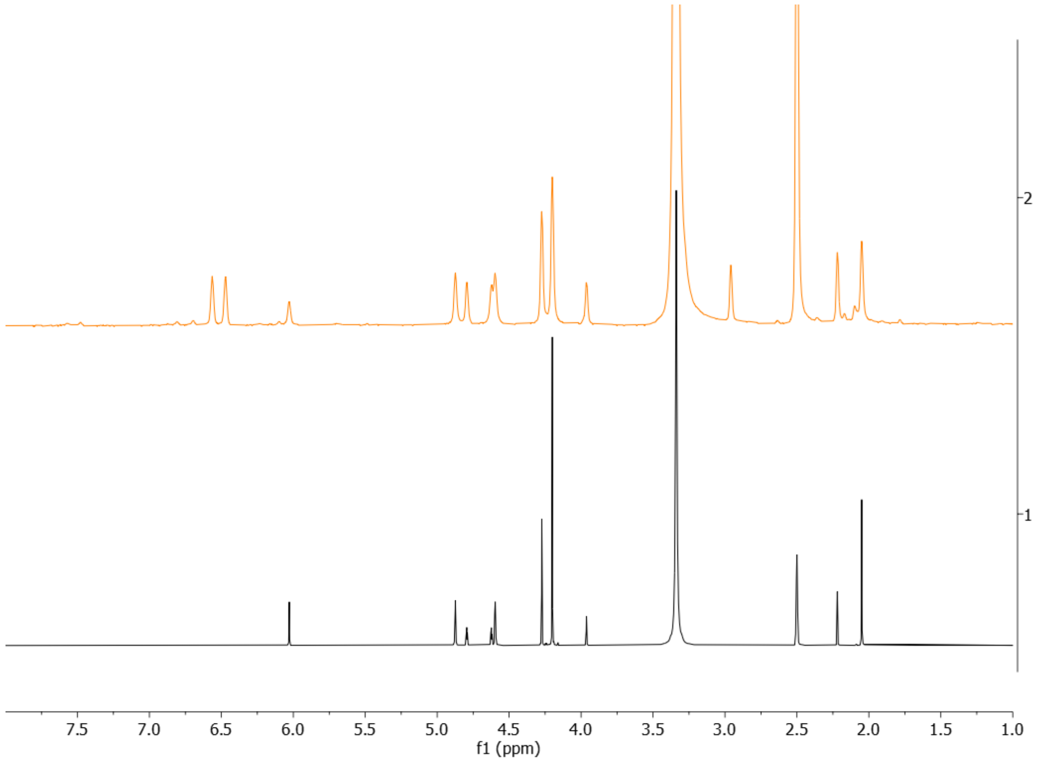

Figure S 20: Ligand L1 at initial (black) and 96 hours (orange) (DMSO-d$_6$, 500 MHz).

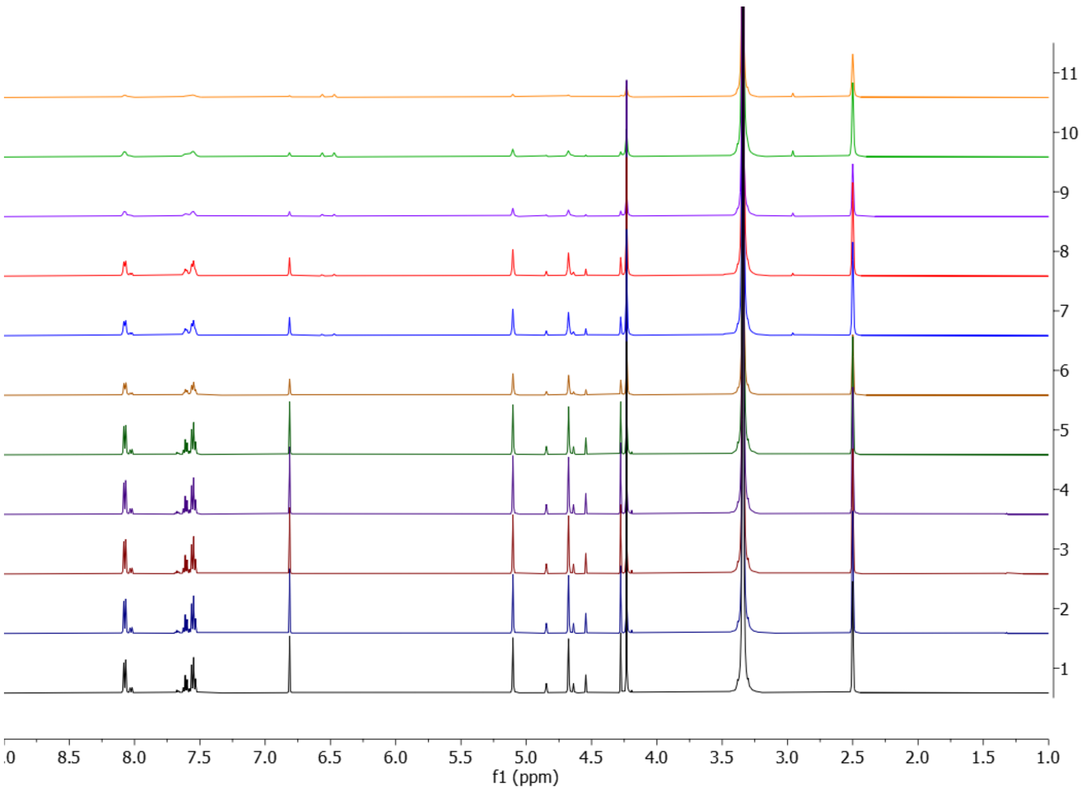

Figure S 21: Ligand L2 over 96 hours (DMSO-d$_6$, 500 MHz).

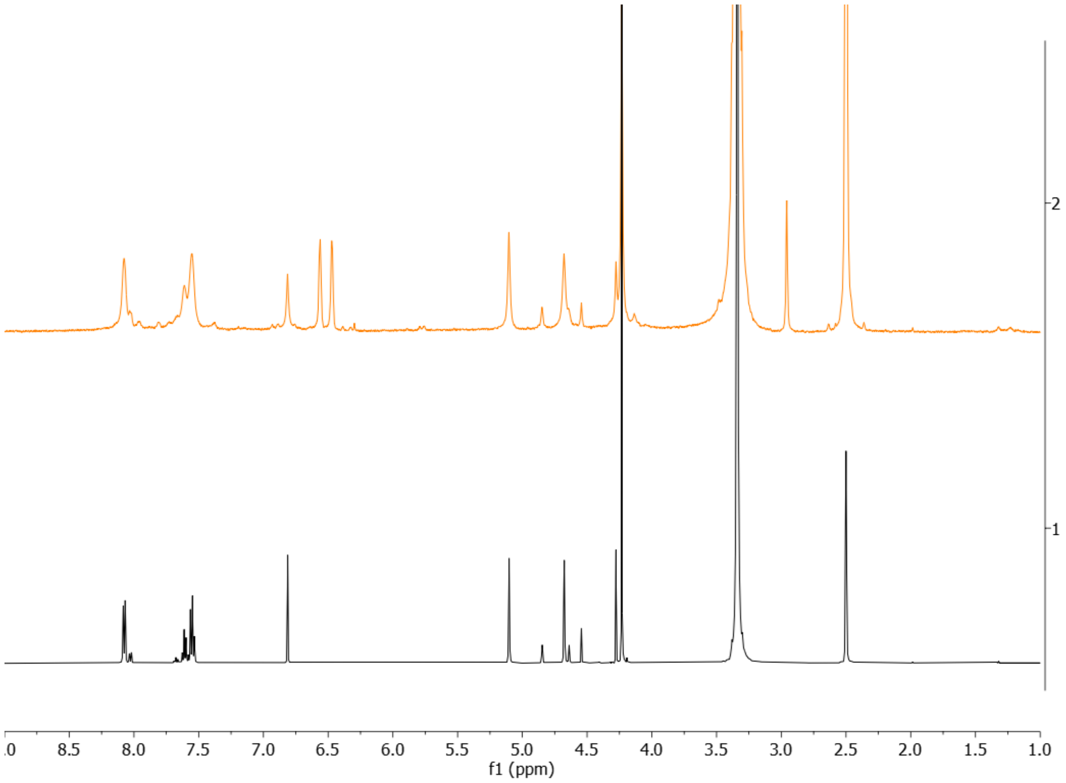

Figure S 22: Ligand L2 at initial (black) and 96 hours (orange) (DMSO-d$_6$, 500 MHz).

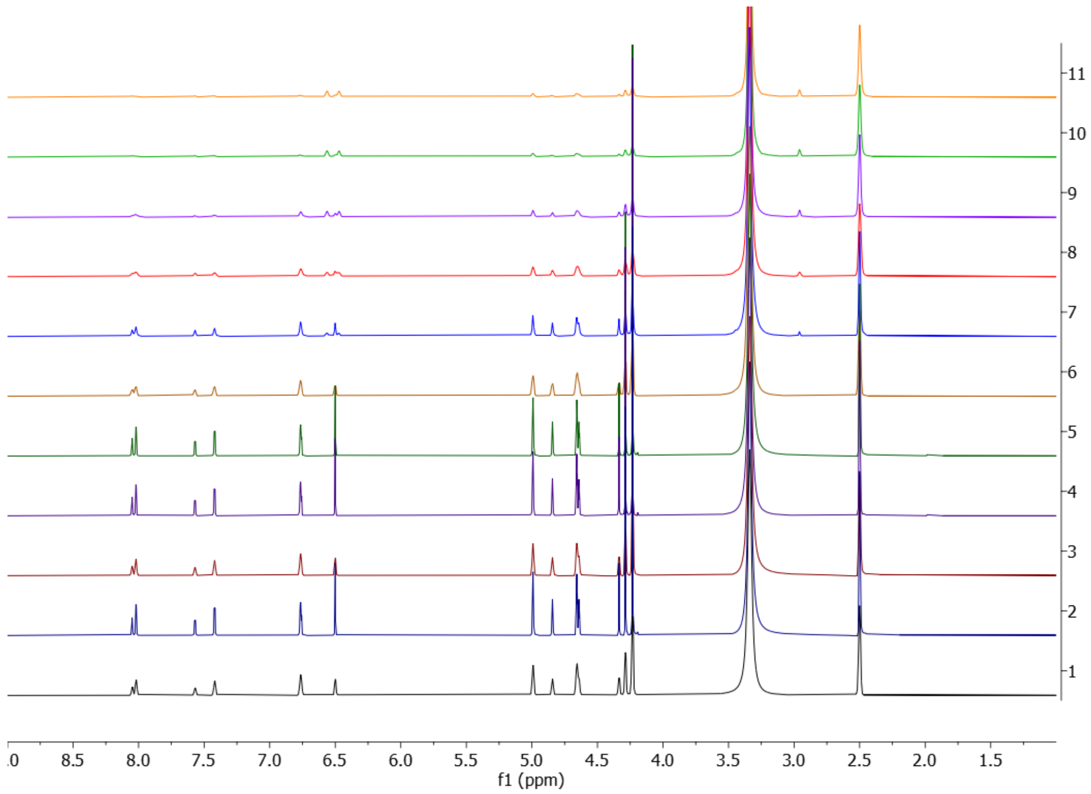

Figure S 23: Ligand L7 over 96 hours (DMSO-d₆, 500 MHz).

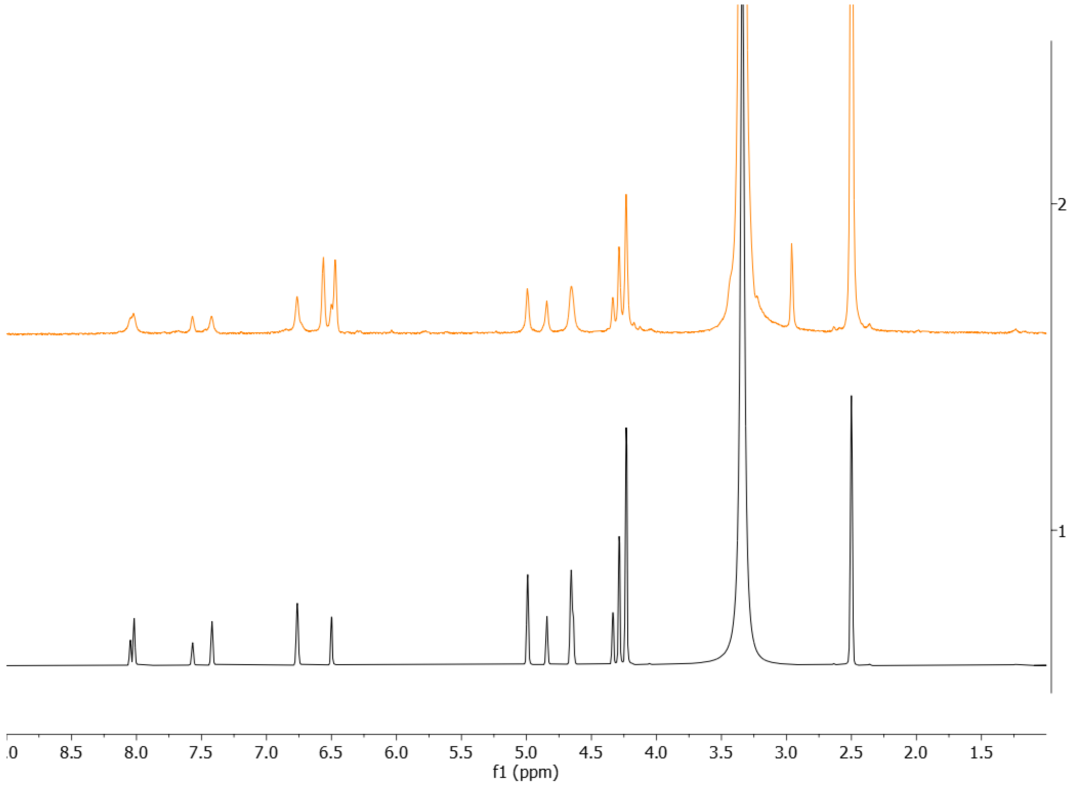

Figure S 24: Ligand L7 at initial (black) and 96 hours (orange) (DMSO-d₆, 500 MHz).

Ligands in DMSO + 10% D_2O

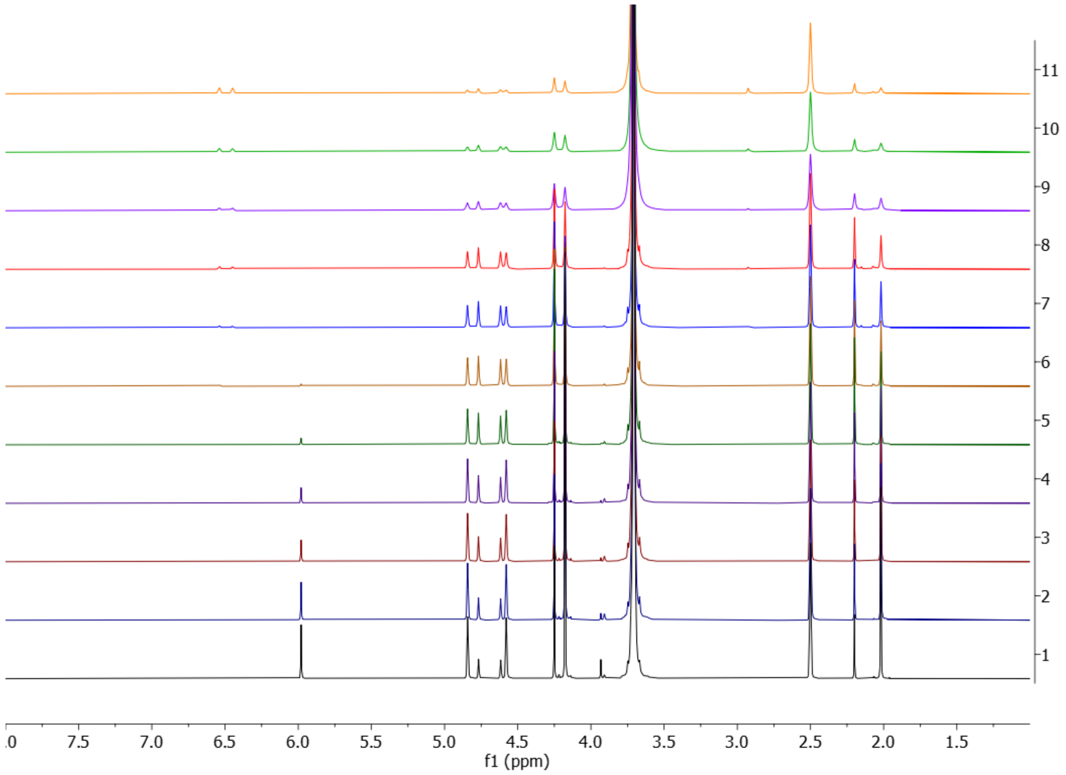

Figure S 25: Ligand L1 over 96 hours (90% DMSO-d_6 + 10% D_2O, 500 MHz).

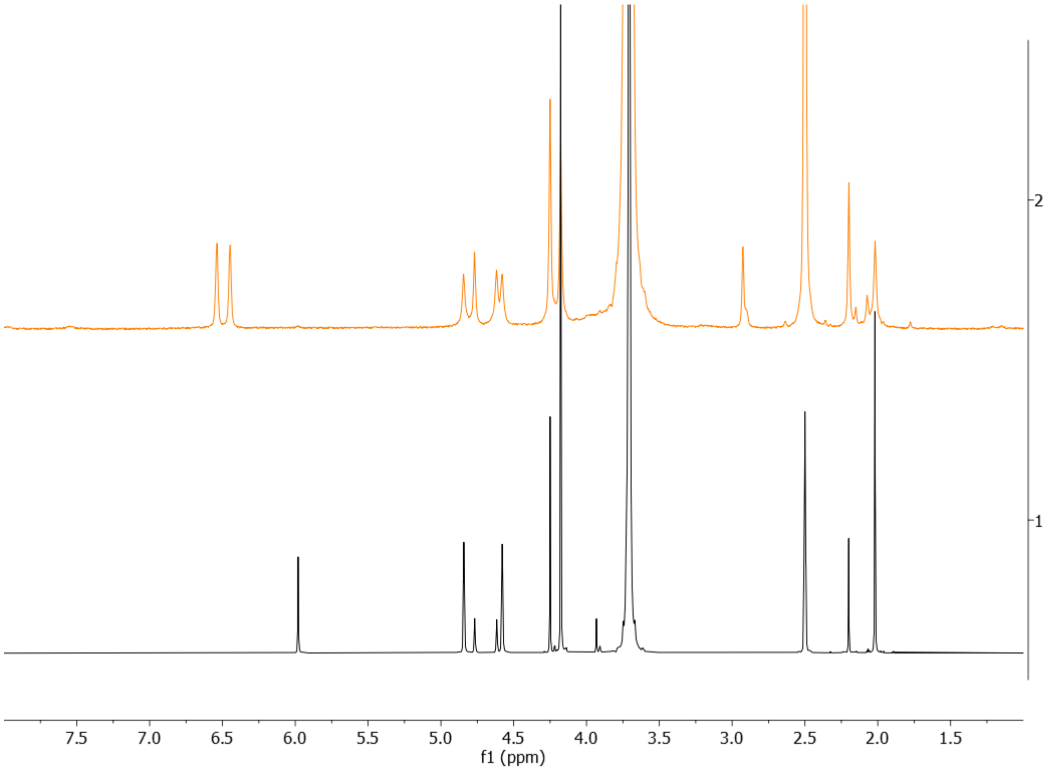

Figure S 26: Ligand L1 at initial (black) and 96 hours (orange) (90% DMSO-d_6 + 10% D_2O, 500 MHz).

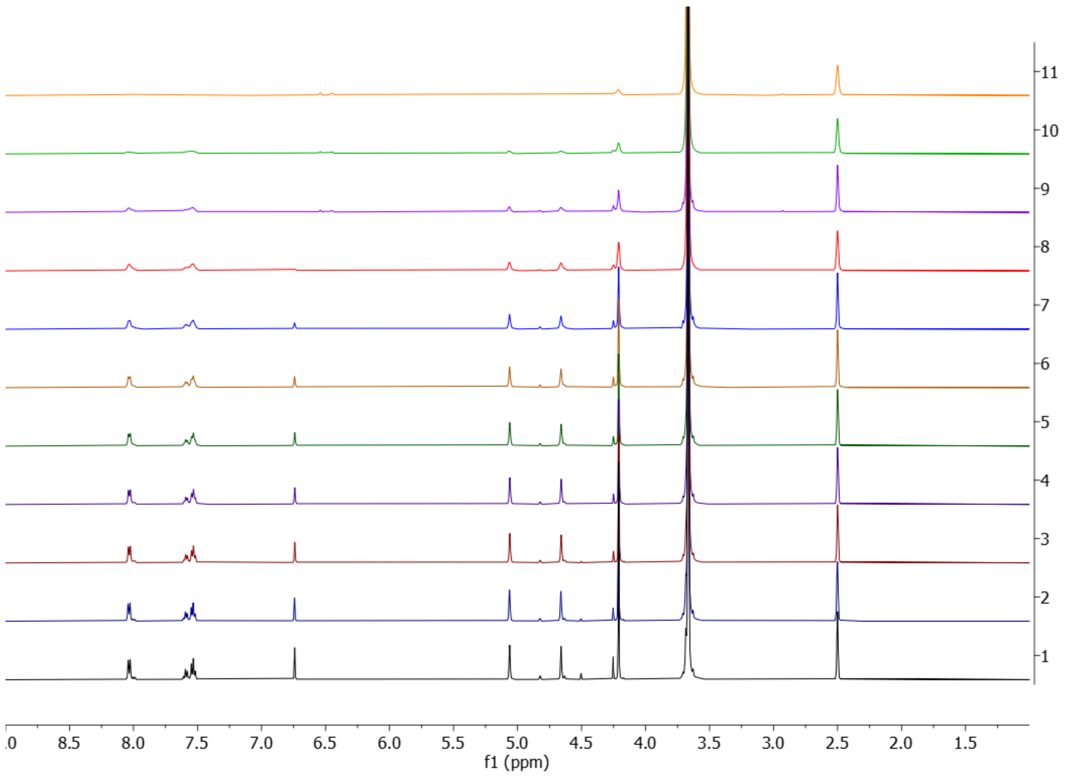

Figure S 27: Ligand L2 over 96 hours (90% DMSO-d_6 + 10% D_2O, 500 MHz).

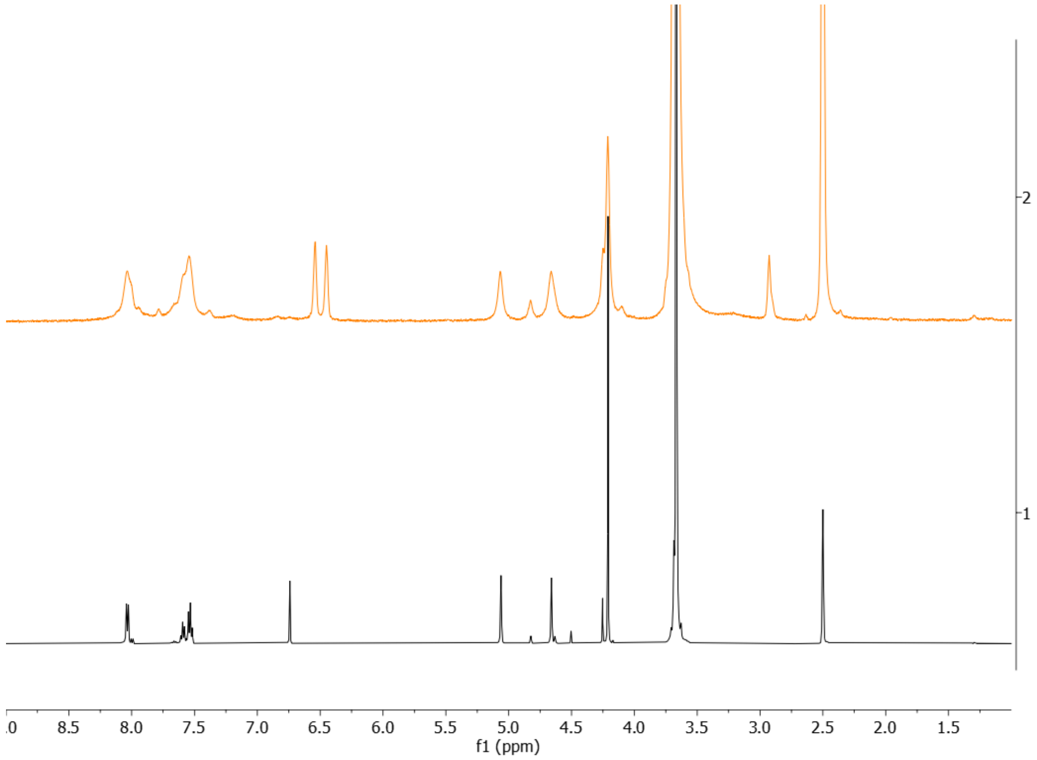

Figure S 28: Ligand L2 at initial (black) and 96 hours (orange) (90% DMSO-d_6 + 10% D_2O, 500 MHz).

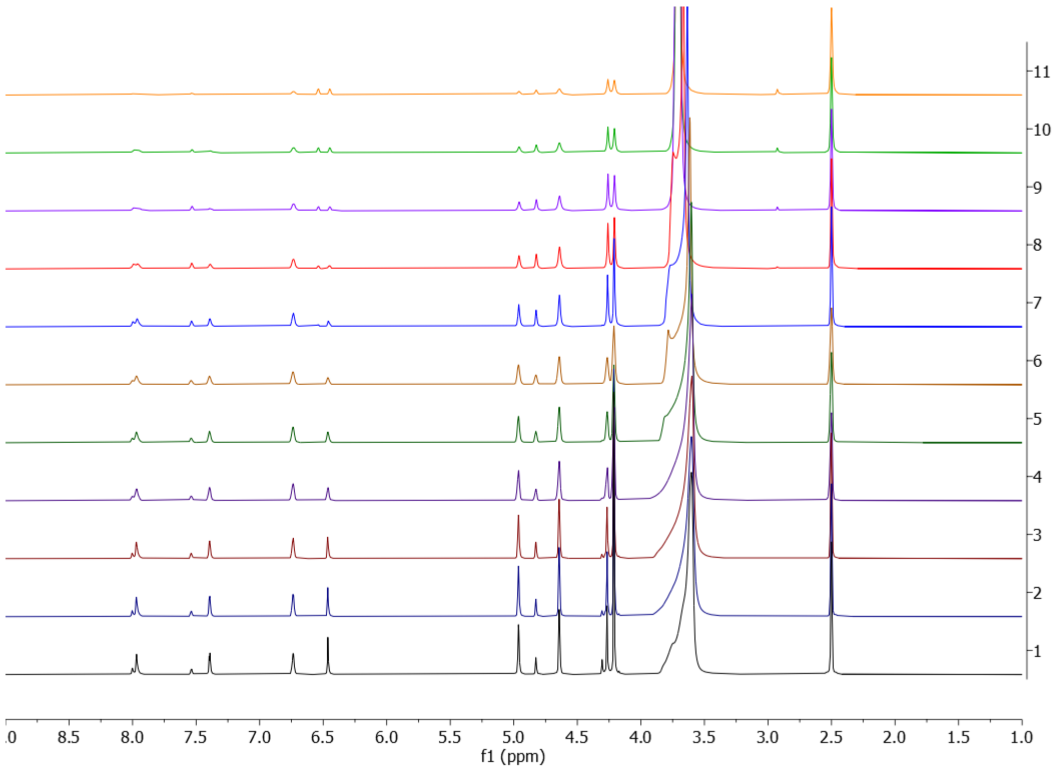

Figure S 29: Ligand L4 over 96 hours (90% DMSO-d_6 + 10% D_2O, 500 MHz).

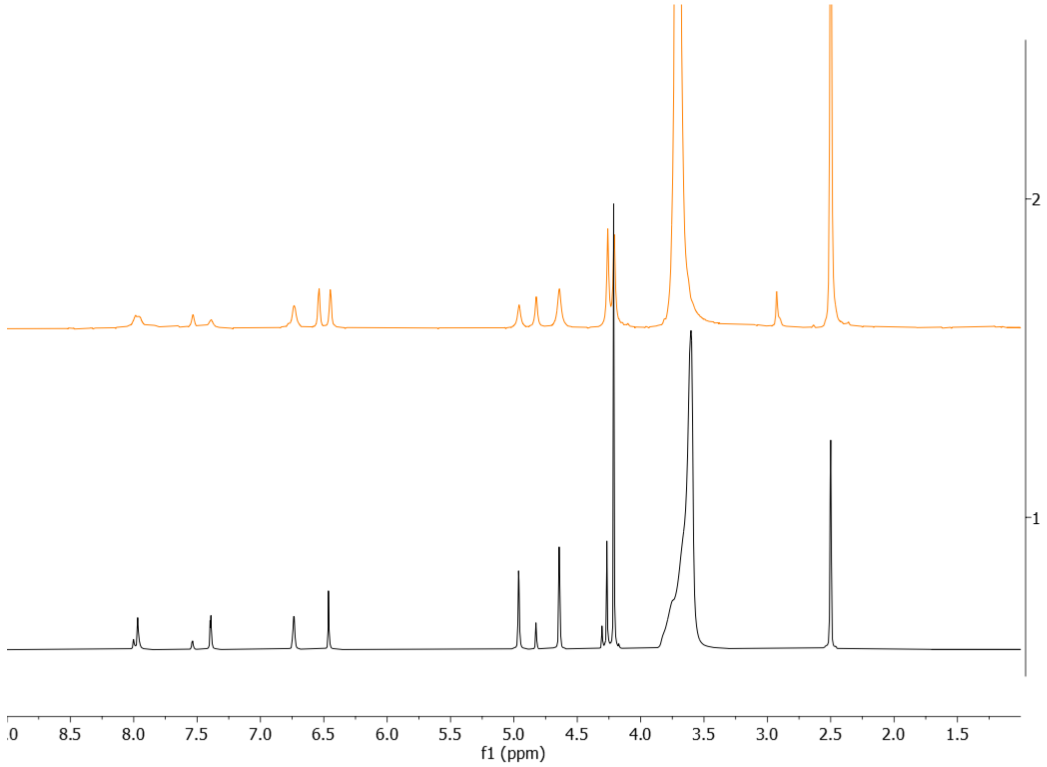

Figure S 30: Ligand L4 at initial (black) and 96 hours (orange) (90% DMSO-d_6 + 10% D_2O, 500 MHz).

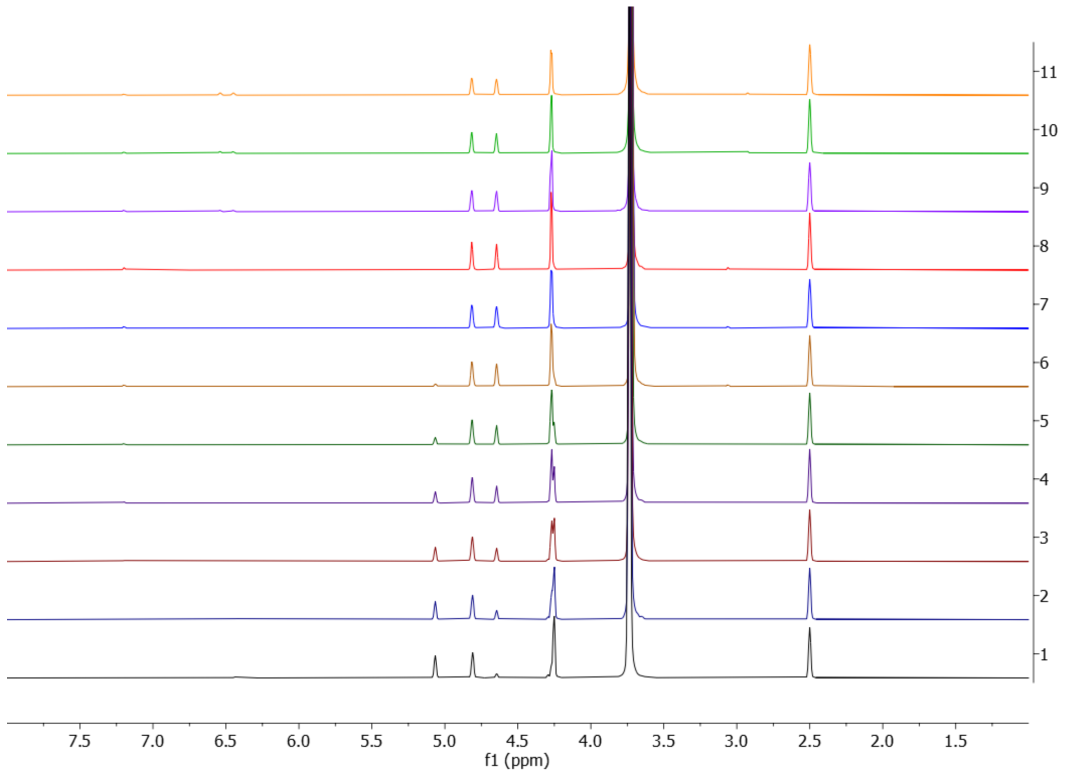

Figure S 31: Ligand L7 over 96 hours (90% DMSO-d_6 + 10% D_2O, 500 MHz).

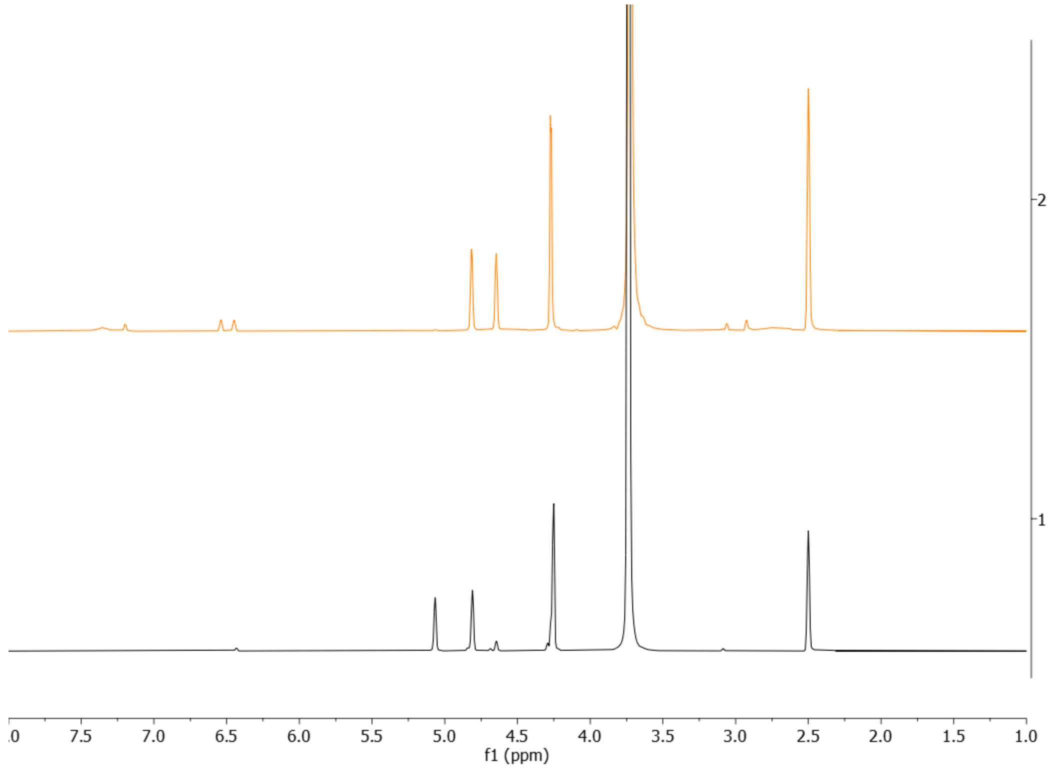

Figure S 32: Ligand L7 at initial (black) and 96 hours (orange) (90% DMSO-d_6 + 10% D_2O, 500 MHz).

NMR Stability Studies of Ruthenium Ferrocenyl Complexes

All spectra for complexes were obtained after **initial (black, 1)**, **20 mins (dark blue, 2)**, **40 mins (dark red, 3)**, 1 h (dark purple, 4), **2 h (dark green, 5)**, **6 h (brown, 6)**, **12 h (blue, 7)**, **24 h (red, 8)**, **48 h (purple, 9)**, **72 h (green, 10)** and **96 h (orange, 11)**.

Complexes in DMSO

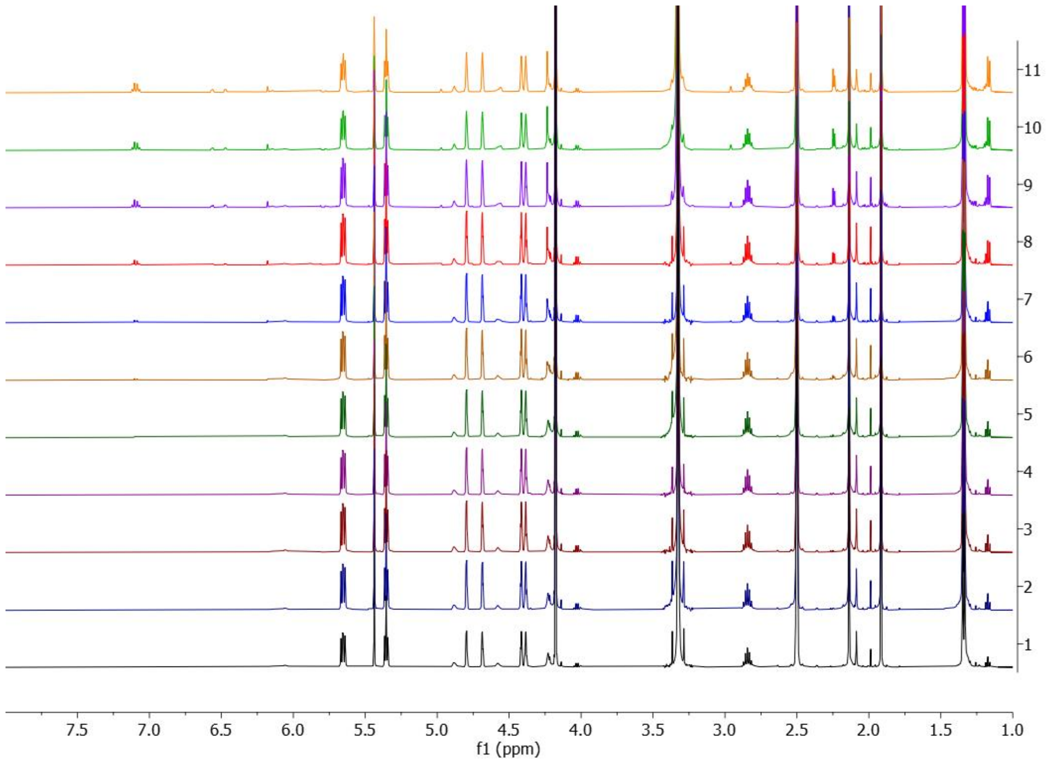

Figure S 33: Complex 1 over 96 hours (DMSO-d_6, 500 MHz).

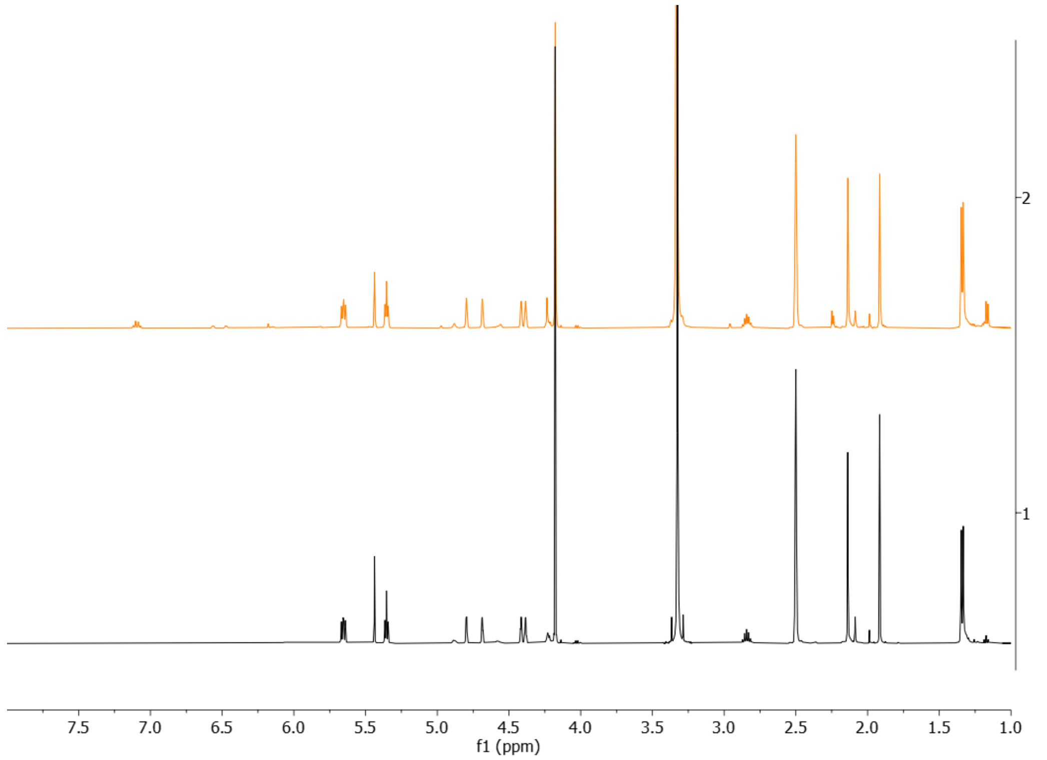

Figure S 34: Complex 1 at initial (black) and 96 hours (orange) (DMSO-d_6, 500 MHz).

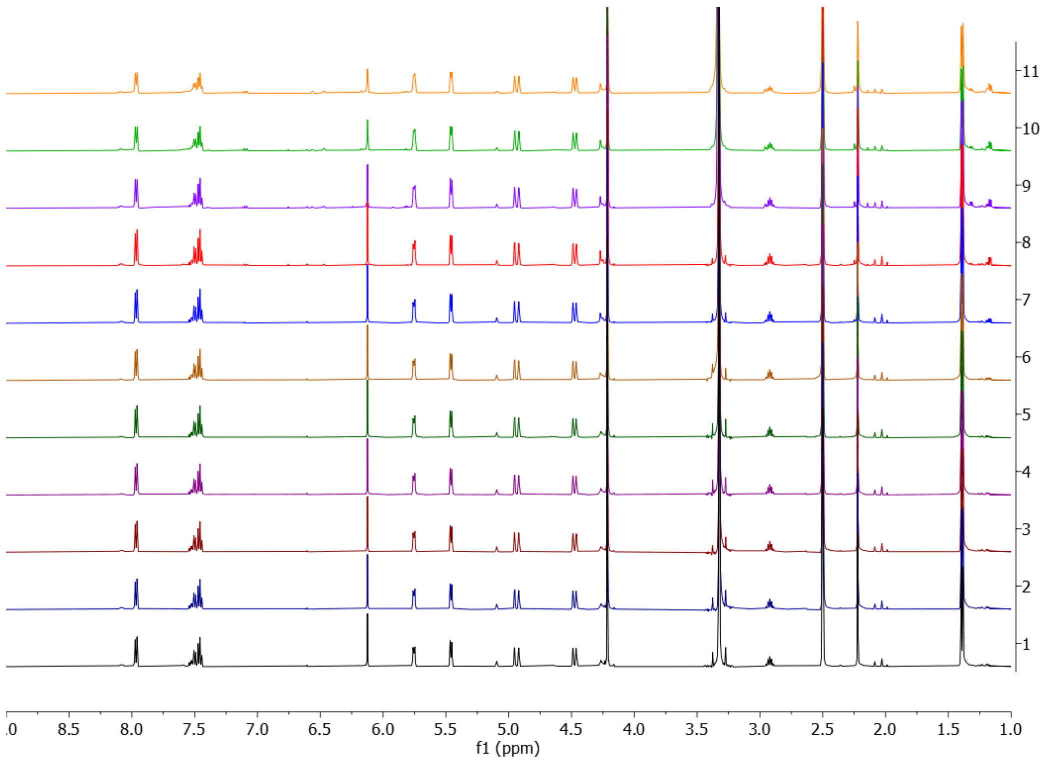

Figure S 35: Complex 2 over 96 hours (DMSO-d6, 500 MHz).

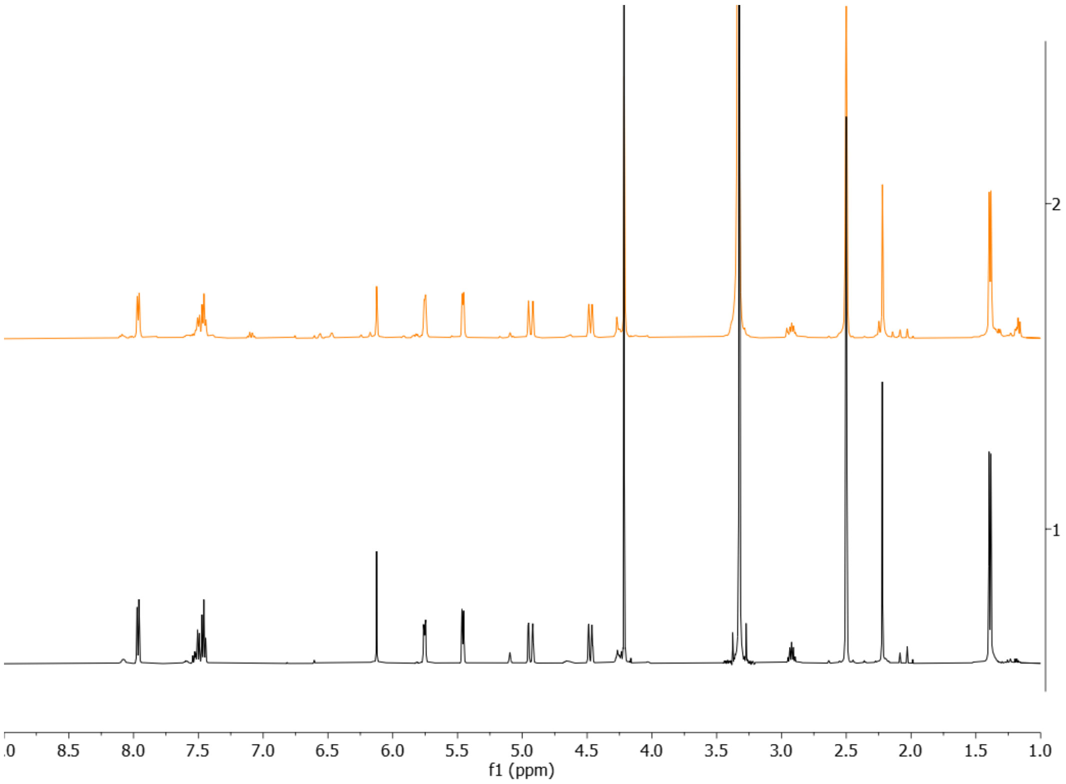

Figure S 36: Complex 2 at initial (black) and 96 hours (orange) (DMSO-d_6, 500 MHz).

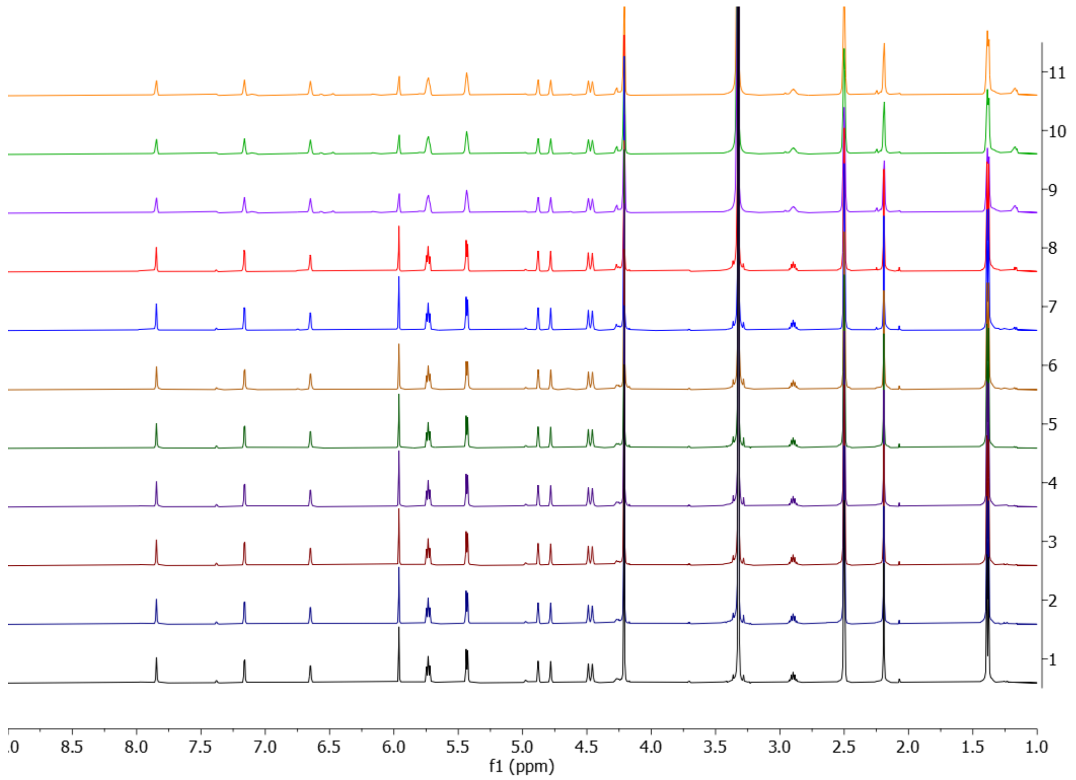

Figure S 37: Complex 4 over 96 hours (DMSO-d6, 500 MHz).

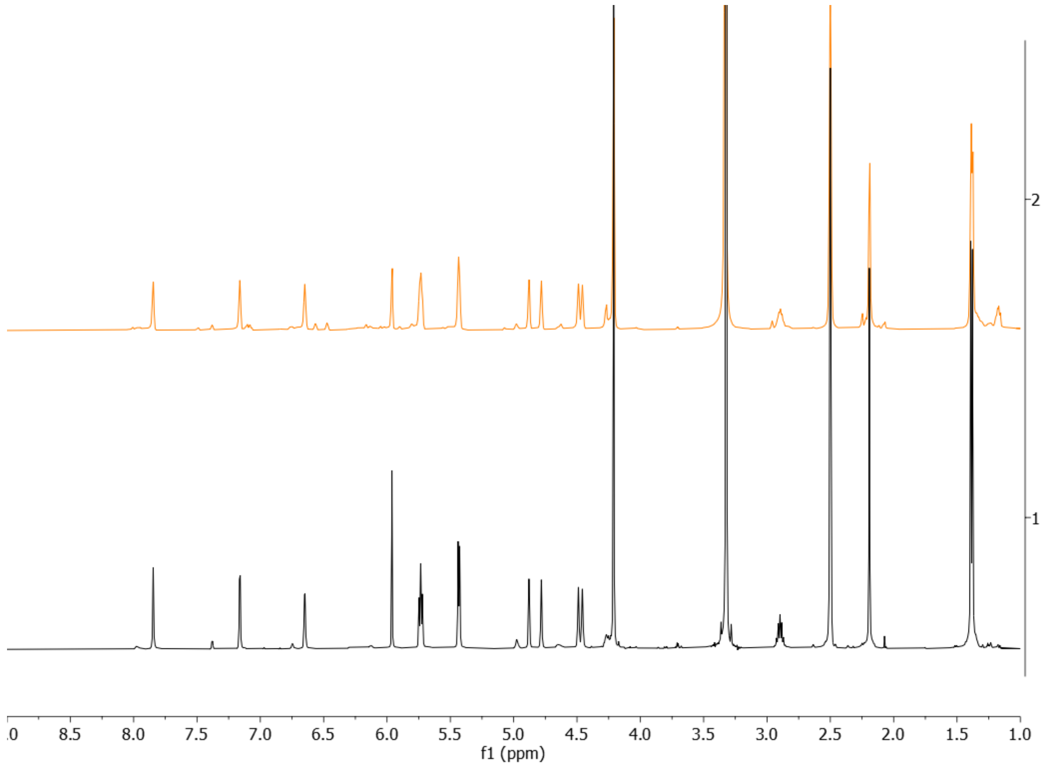

Figure S 38: Complex 4 at initial (black) and 96 hours (orange) (DMSO-d$_6$, 500 MHz).

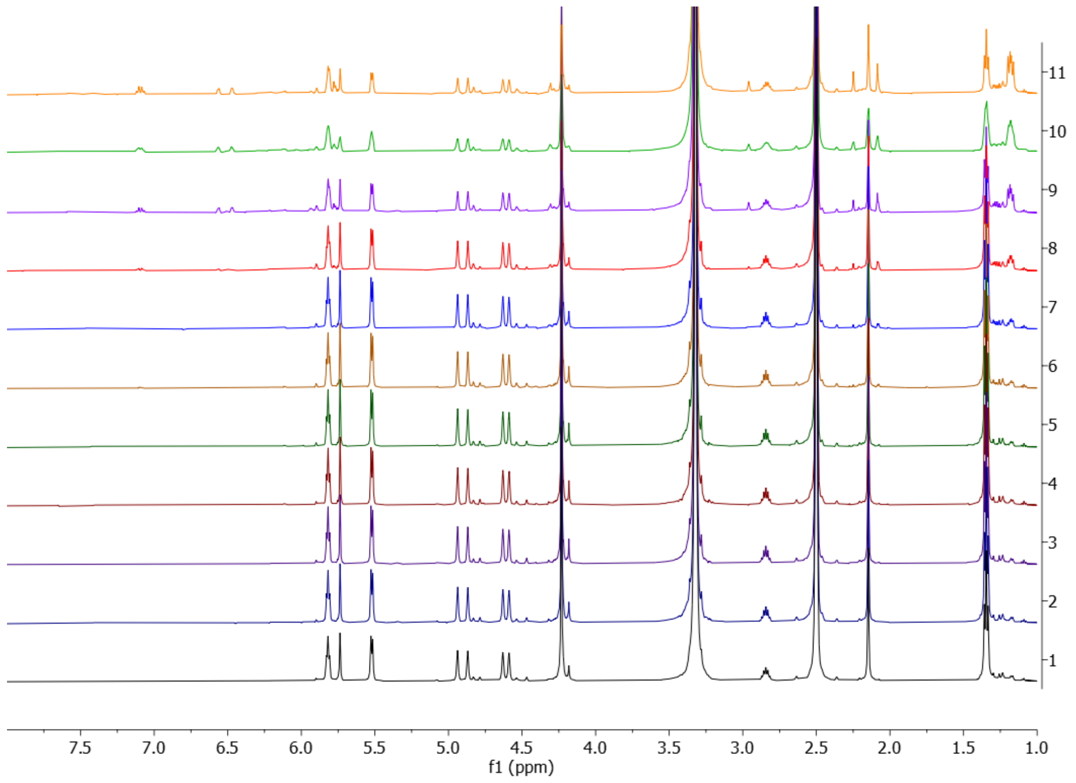

Figure S 39: Complex 7 over 96 hours (DMSO-d_6, 500 MHz).

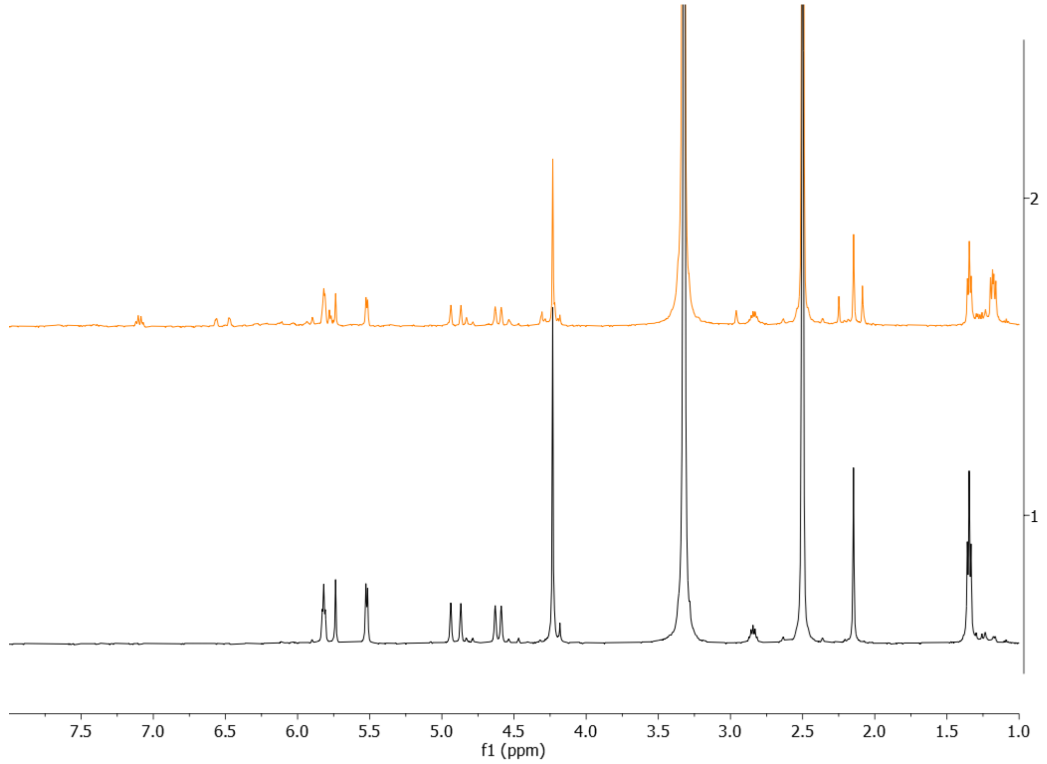

Figure S 40: Complex 7 at initial (black) and 96 hours (orange) (DMSO-d_6, 500 MHz).

Complexes in DMSO + 10% D_2O

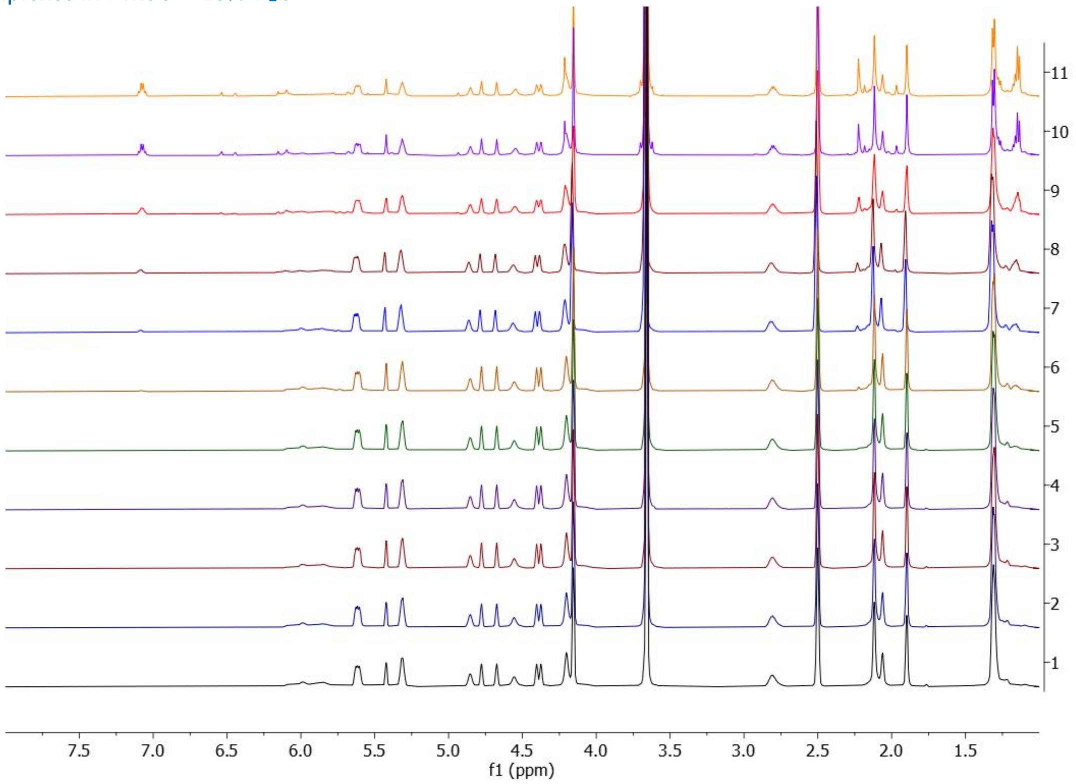

Figure S 41: Complex 1 over 96 hours (90% DMSO-d_6 + 10% D_2O, 500 MHz).

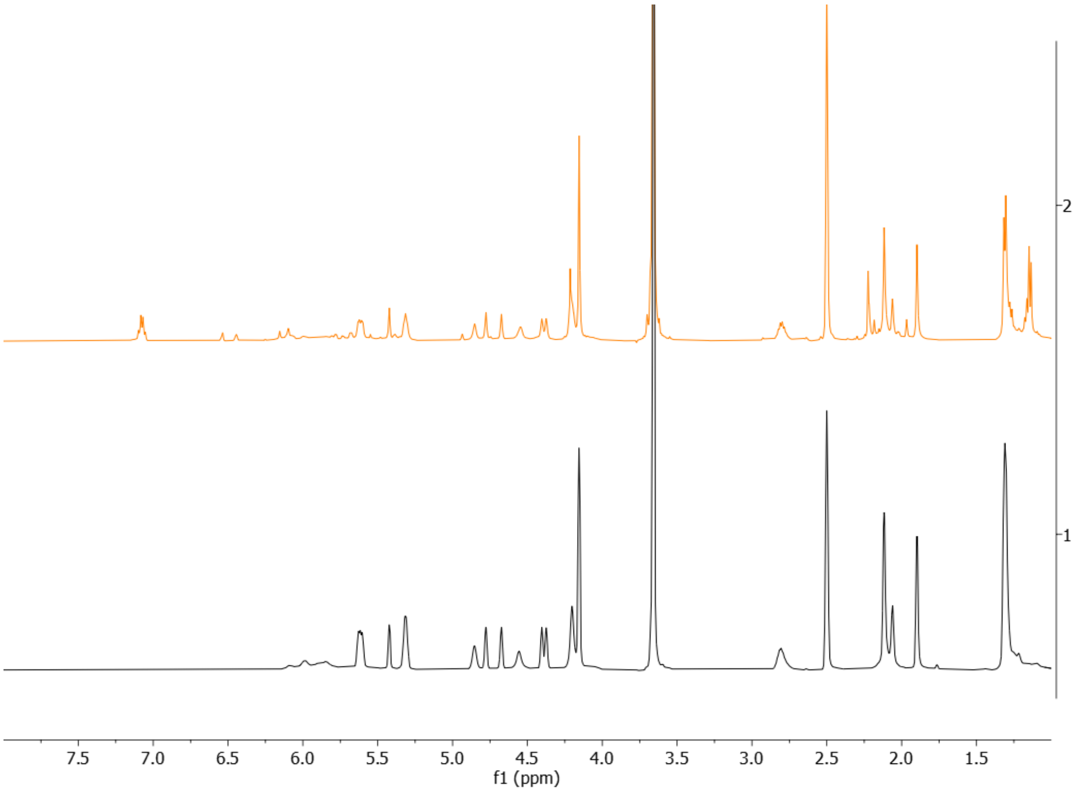

Figure S 42: Complex 1 at initial (black) and 96 hours (orange) (90% DMSO-d_6 + 10% D_2O, 500 MHz).

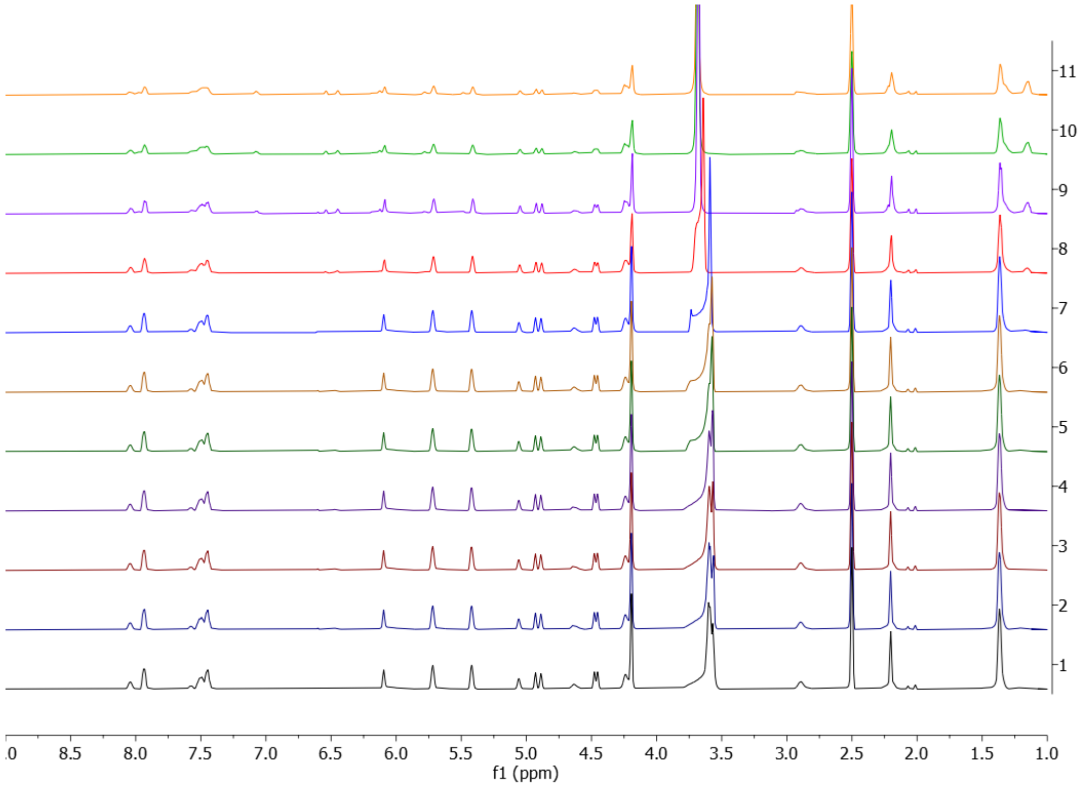

Figure S 43: Complex 2 over 96 hours (90% DMSO-d_6 + 10% D_2O, 500 MHz).

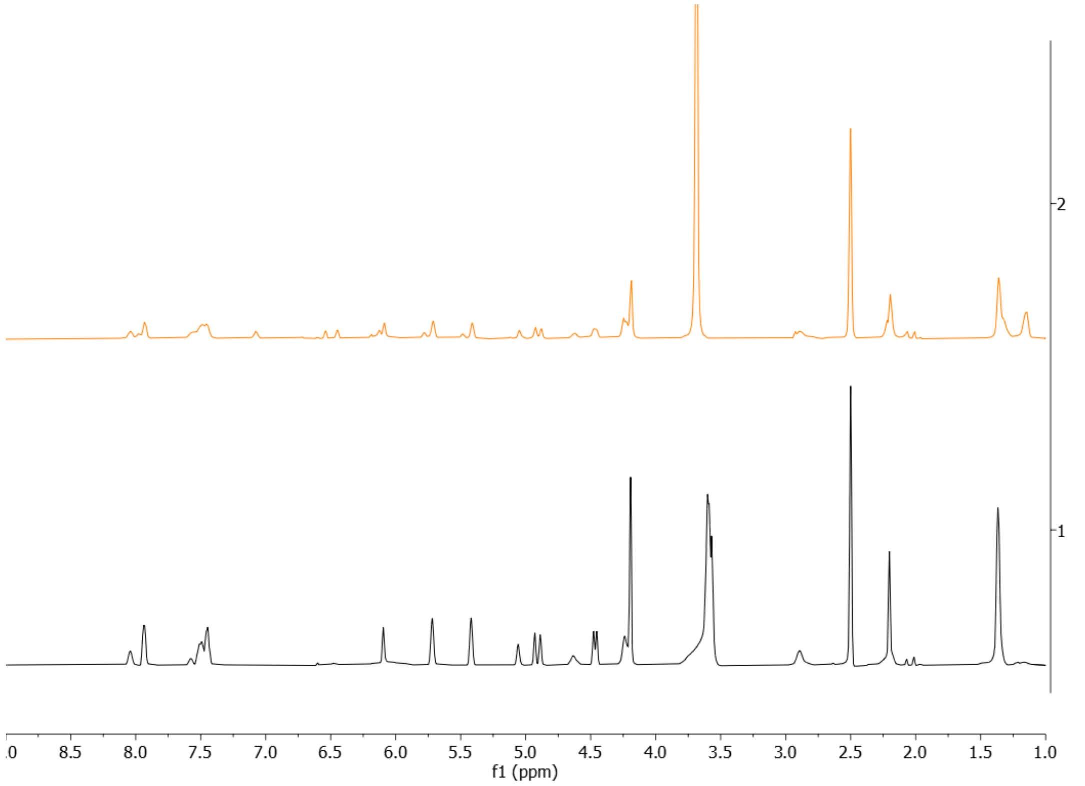

Figure S 44: Complex 2 at initial (black) and 96 hours (orange) (90% DMSO-d_6 + 10% D_2O, 500 MHz).

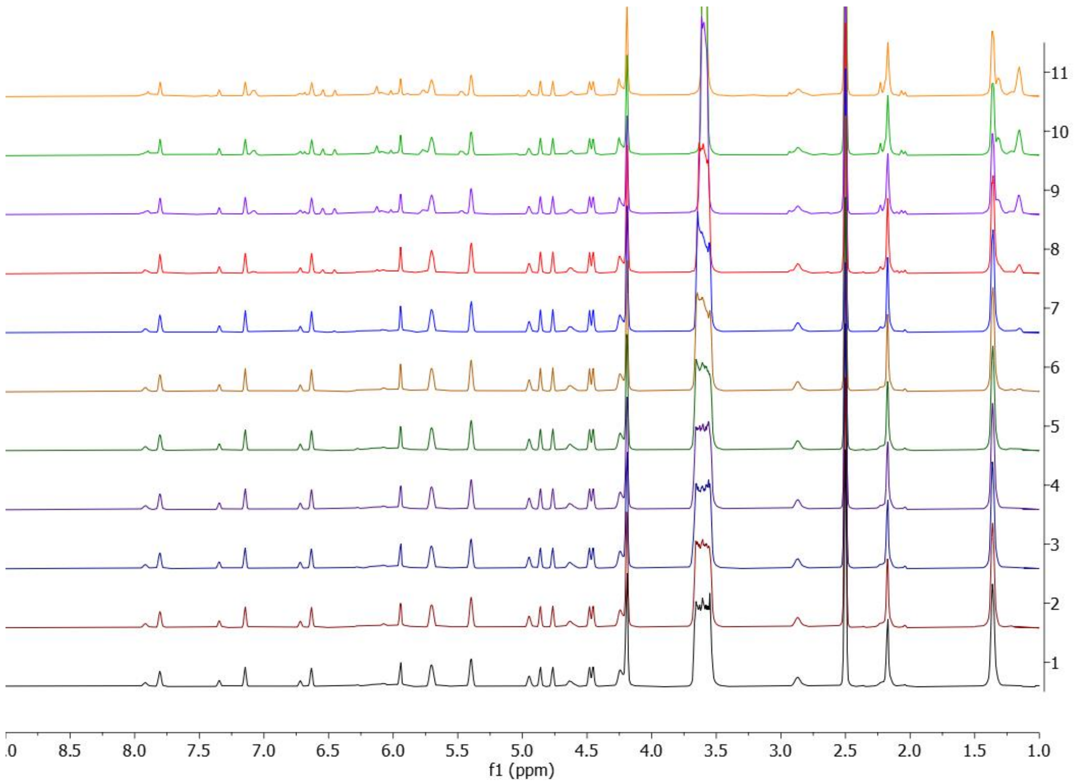

Figure S 45: Complex 4 over 96 hours (90% DMSO-d_6 + 10% D_2O, 500 MHz).

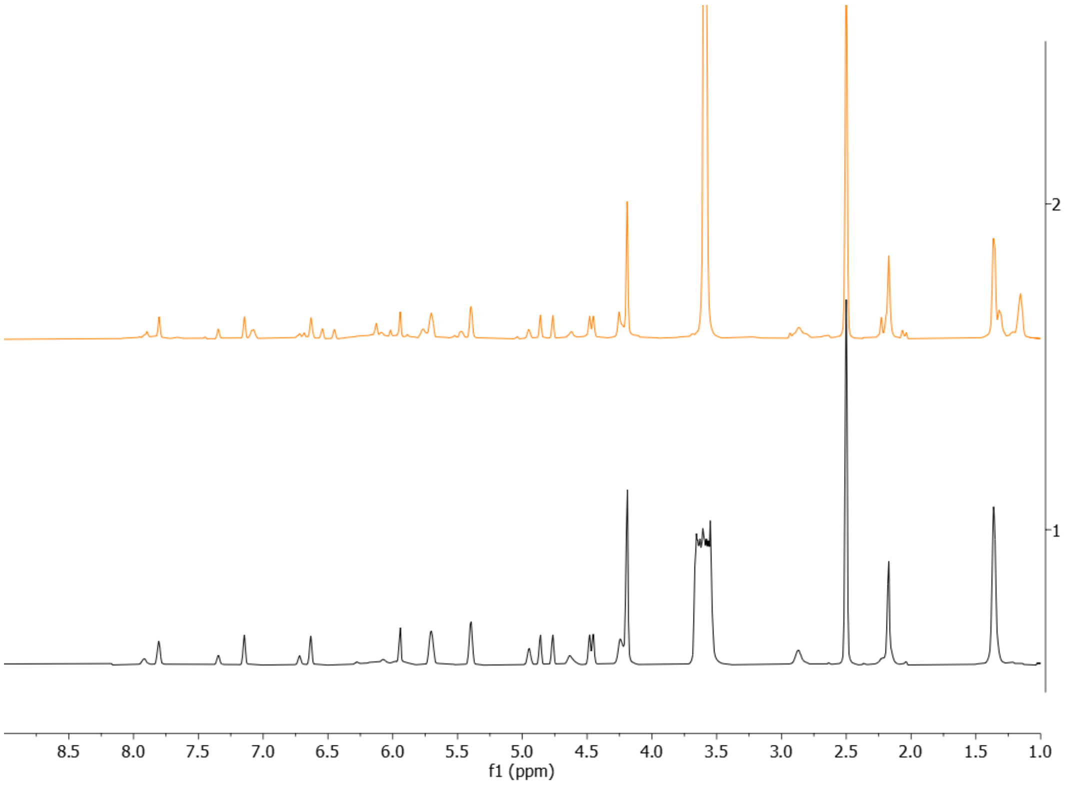

Figure S 46: Complex 4 at initial (black) and 96 hours (orange) (90% DMSO-d_6 + 10% D_2O, 500 MHz).

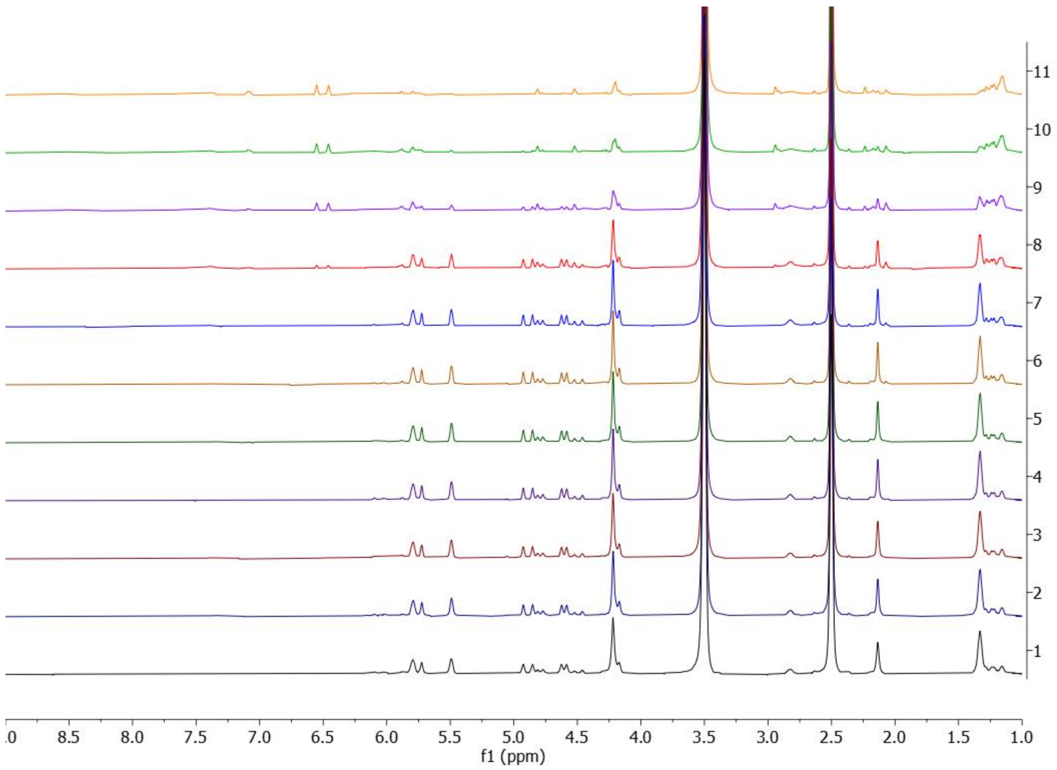

Figure S 47: Complex 7 over 96 hours (90% DMSO-d_6 + 10% D_2O, 500 MHz).

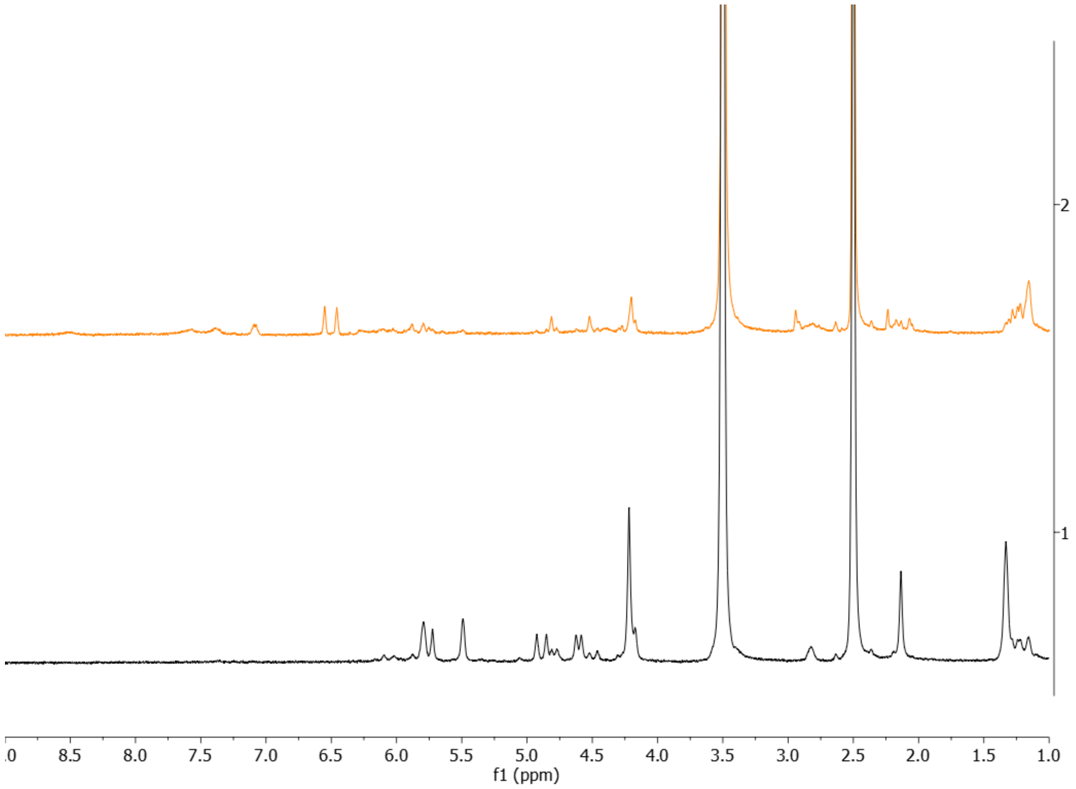

Figure S 48: Complex 7 at initial (black) and 96 hours (orange) (90% DMSO-d_6 + 10% D_2O, 500 MHz).

Complex 1 overlay

DMSO + 10% D_2O – showing decomposition

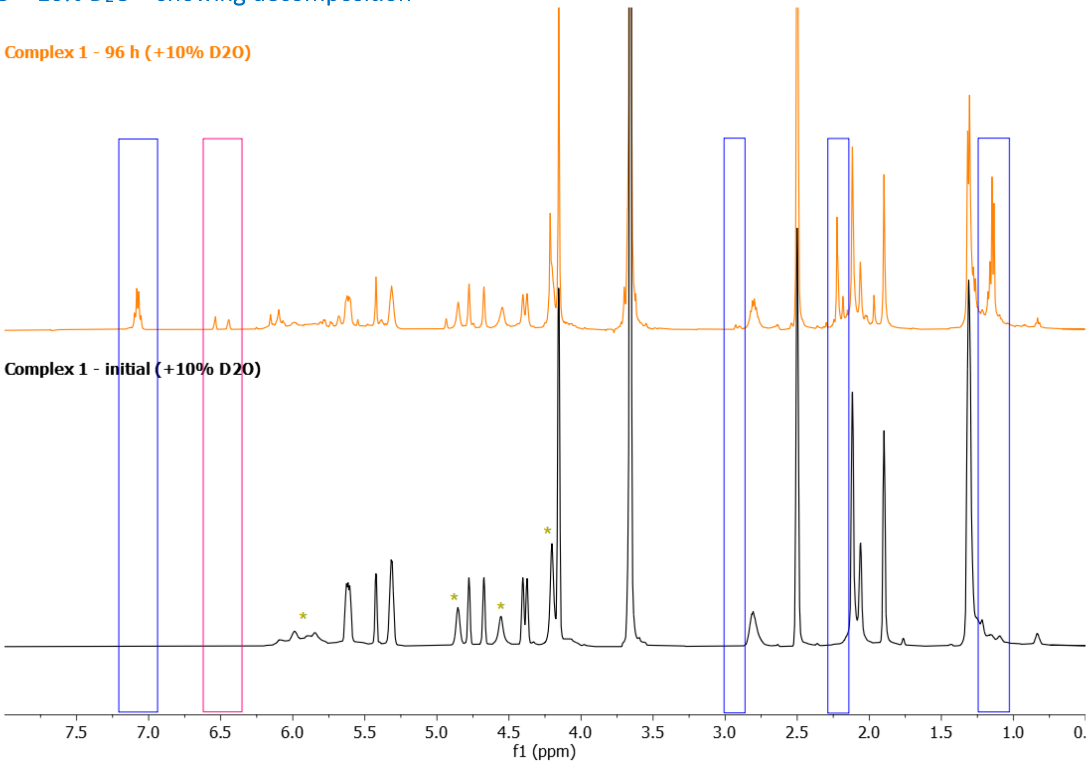

Figure S 49: Complex 7 at initial (black) and 96 hours (orange) (90% DMSO-d_6 + 10% D_2O, 500 MHz) – showing free p-cymene in blue boxes and free Cp in a pink box (* are also shown for the dimerization of the Ru complex, which is observed in highly polar solvents, e.g., DMSO).

DMSO + 10% D_2O + 100 mM NaCl

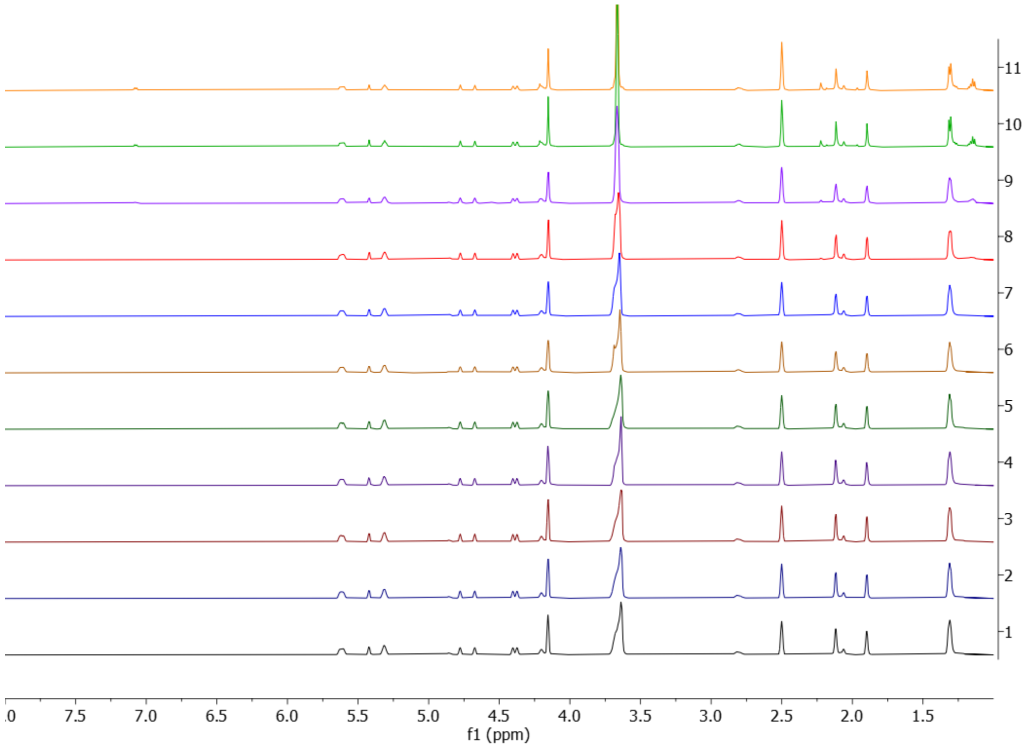

Figure S 50: Complex 1 over 96 hours + 100 mM NaCl (90% DMSO-d_6 + 10% D_2O, 500 MHz).

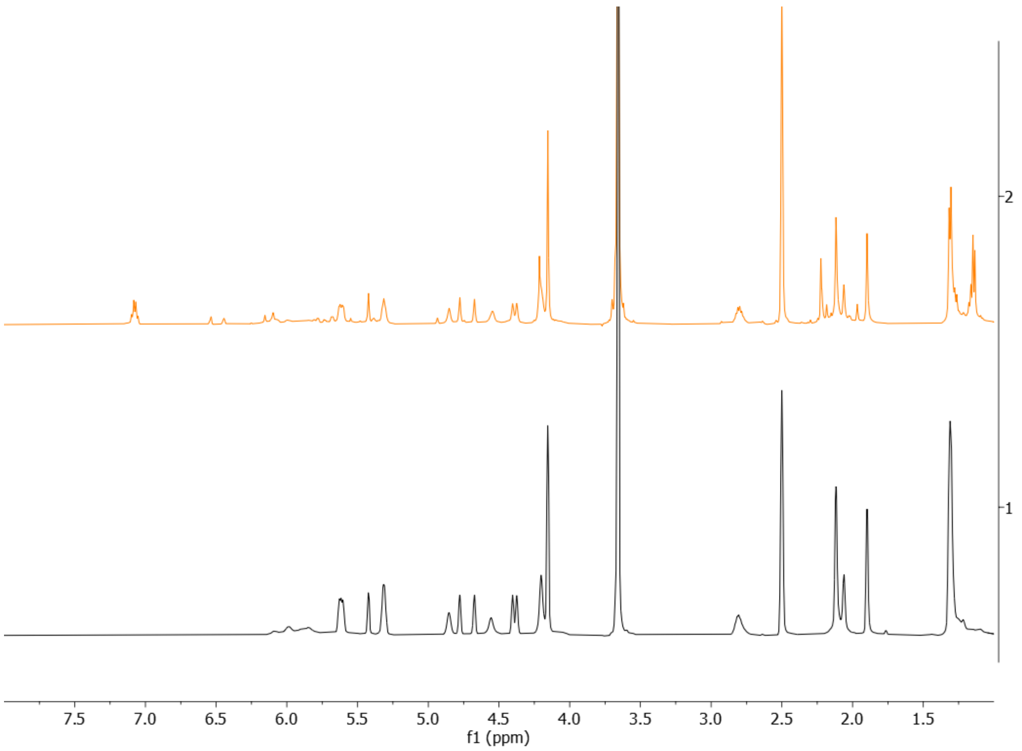

Figure S 51: Complex 1 at initial (black) and 96 hours (orange) + 100 mM NaCl (90% DMSO-d_6 + 10% D_2O, 500 MHz).

DMSO + 10% D_2O – comparison with and without 100 mM NaCl

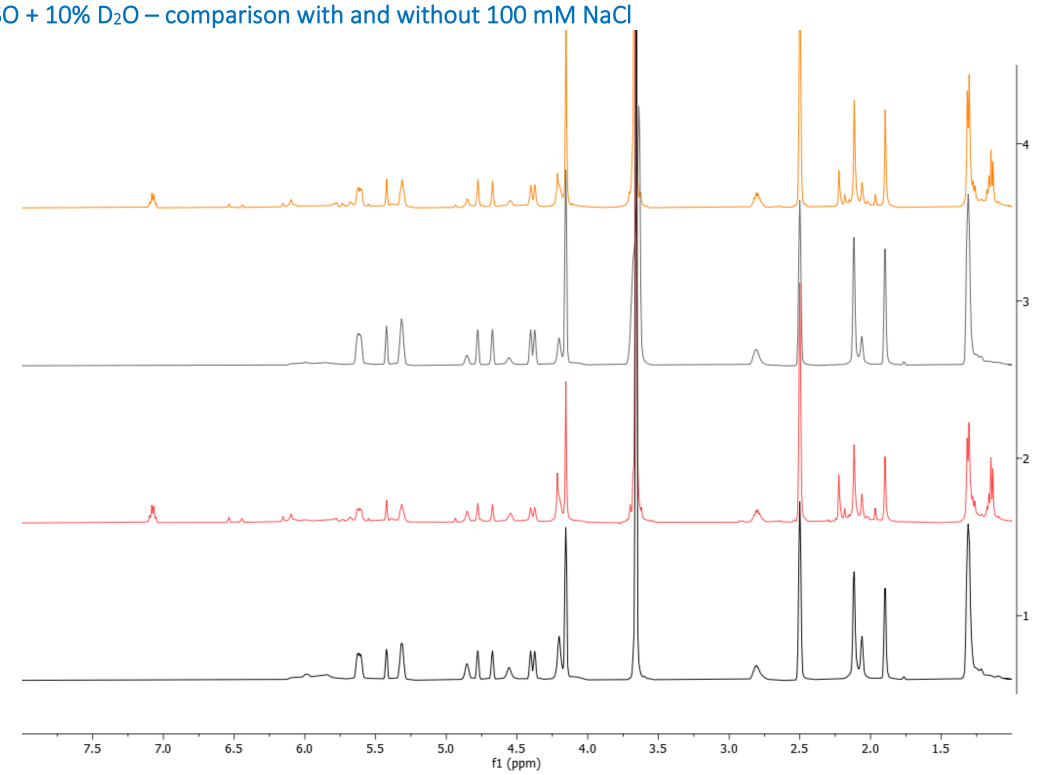

Figure S 52: Complex 1 at initial without NaCl (black) and 96 hours (red) and initial with 100 mM NaCl (black) and 96 hours (orange) (90% DMSO-d_6 + 10% D_2O, 500 MHz).

Cyclic Voltammetry

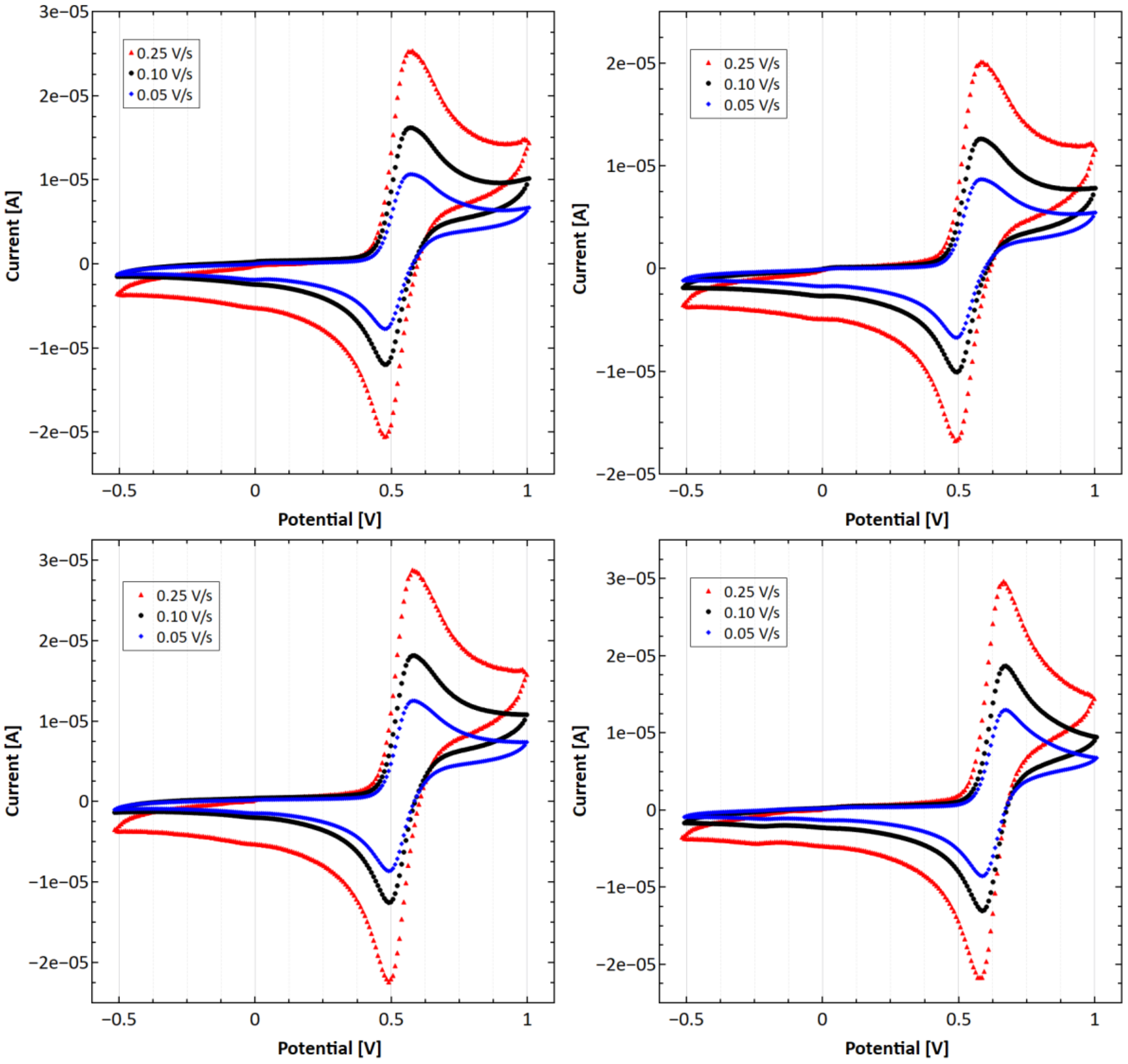

Figure S 53: Cyclic voltammograms of 1 (top left), 2 (top right), 4 (bottom left) and 7 (bottom right) in the electrochemical window between -0.5 – 1 V.

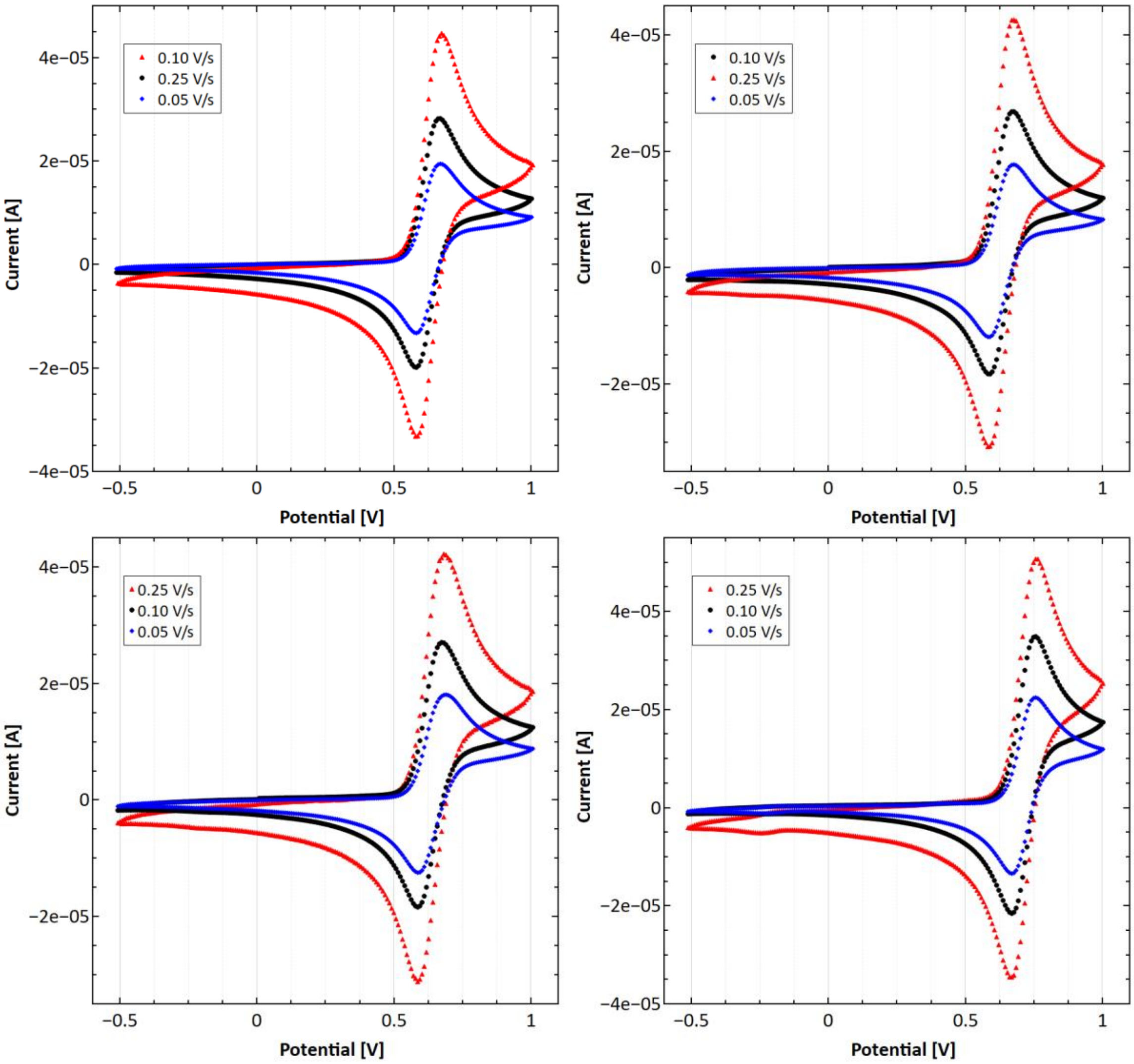

Figure S 54: Cyclic voltammograms of L1 (top left), L2 (top right), L4 (bottom left) and L7 (bottom right) in the electrochemical window between -0.5 – 1 V.

Table S 17: Redox potentials and peak separation ΔE_P for compounds 1, 2, 4 and 7 and their corresponding ligands.

	**0.05 V/s**		**0.10 V/s**		**0.25 V/s**	
**Compound**	$E^{0\prime}$ [V]	ΔE_P [10^1 mV]	$E^{0\prime}$ [V]	ΔE_P [10^1 mV]	$E^{0\prime}$ [V]	ΔE_P [10^1 mV]
**1**	0.53	9	0.53	9	0.53	8
**2**	0.54	8	0.54	8	0.54	9
**4**	0.54	8	0.54	8	0.54	9
**7**	0.63	8	0.63	8	0.62	9
**L1**	0.63	8	0.62	8	0.63	9
**L2**	0.63	9	0.63	8	0.63	8
**L4**	0.64	9	0.63	8	0.63	9
**L7**	0.71	8	0.71	8	0.71	9

All compounds exhibit a fully reversible single electron redox couple (assigned to the Fc^*/Fc^{*+}) with a peak separation (ΔE_P) between 8 – 9 x 10^1 mV, $i_{PA}/i_{PC} \approx 1$ and $i_{PA} \propto v^{1/2}$.

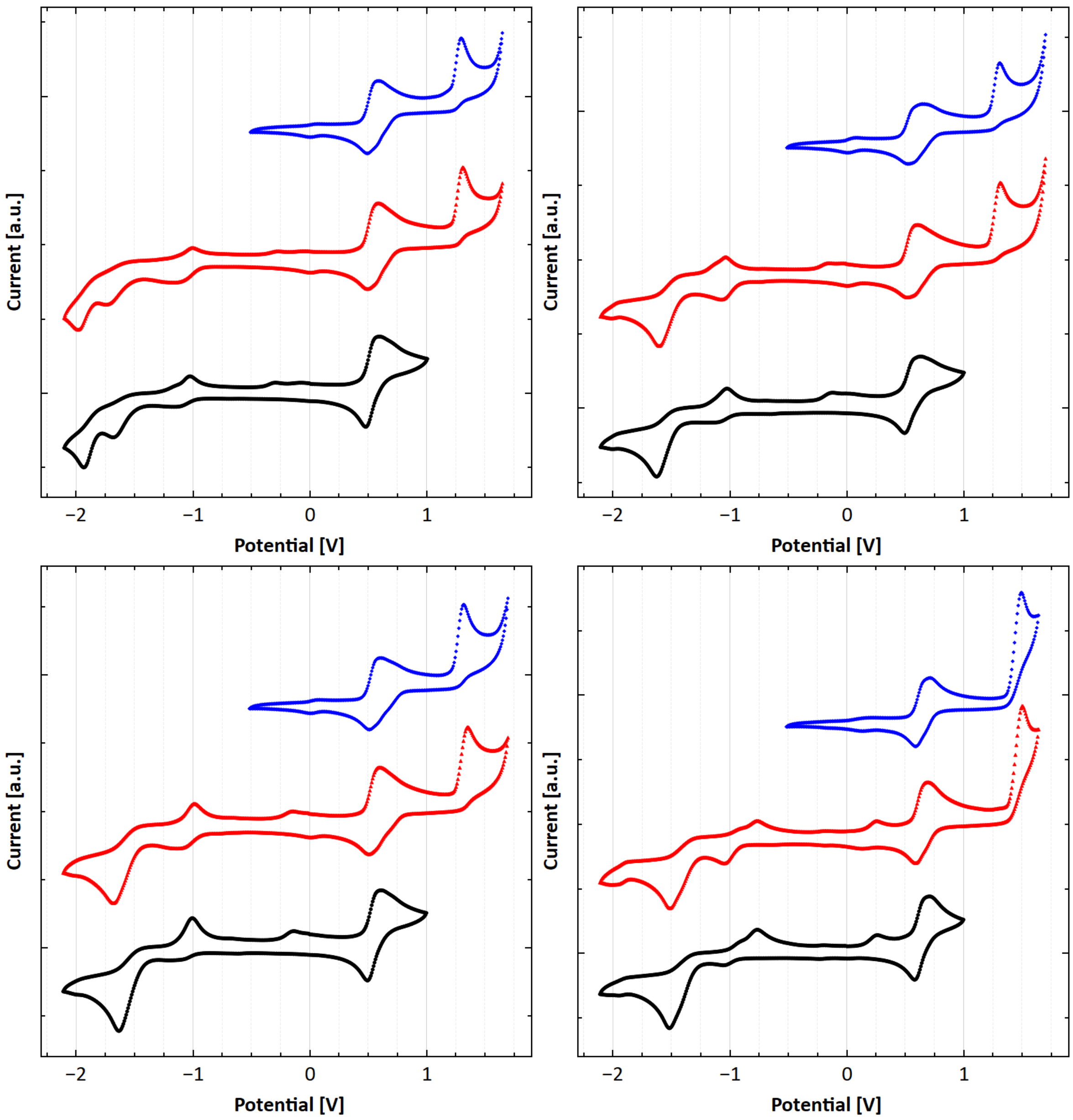

Figure S 55: Cyclic voltammograms of 1 (top left), 2 (top right), 4 (bottom left) and 7 (bottom right) in the electrochemical windows between -0.5 – 1.65 V, -2.1 – 1.65 V and -2.1 – 1 V (from top to bottom).

Table S 18: Redox potentials of significant reduction peaks excluding the Fc*/Fc*⁺ redox couple in the window from -2.1 – 1.65 V. P_C cathodic peak, n.o. = not observed, sh = shoulder.

Compound	P_{C1}	P_{C2}	P_{C3}
**1**	-1.13	-1.66	-1.93
**2**	-1.11	-1.62	n.o.
**4**	-1.10	-1.63	n.o.
**7**	-1.04	-1.40 (sh)	-1.52

The wide scan CVs for compounds **1**, **2**, **4** and **7** exhibit up to three significant reduction peaks (P_C) with a minor signal between -1.04 – -1.13 V and one to two major signals between -1.52 – -1.93 V (P_{C2} and P_{C3}). All three reductions are tentatively assigned to the irreversible reduction of the respective acac ligands with an overall count of two electrons (determined by i_{PC}). The reduction of the chloro complexes is observed in a single step (**2**, **4**) or two steps (**1**, **7**), while the peak separation is very small for **7** and only observed as a shoulder

around -1.40 V. P_{C1} is assigned to the respective solvolyzed complexes at much lower negative potentials between -1.04 – -1.13 V. This assignment is based on a cyclic voltammetry experiment where the concentration of **1** is changed and a chloride source is added (**Figure S 56**). After double dilution of the compound the ratio $i_{K(Fc^*)}/i_{k(PC1)}$ increases from 0.2 to 0.3, pointing to larger amount of a potentially solvolyzed complex. Its identity is proven by addition of a Cl^- source (TBACl, 0.2 mmol). After addition, the peak disappears and the main reduction peak increases so that $i_{PC2} \approx i_{P(Fc^*)}$. Furthermore, the experiment shows that the anodic peak at -1.03 V is linked to the reduction(s) assigned as P_{C2} and P_{C3}. Other minor cathodic signals in the area between -0.31 – 0.25 V arise only after the irreversible 2-e^- reduction (see **Figure S 55** and **S 56**). A similar signal is observed after the irreversible oxidation of Ru(II) → Ru(III) between 0.00 – 0.14 V. Both irreversible redox reactions also lead to a broadening of the signal assigned to the Fc*/Fc*$^+$ redox couple so that the full reversibility is only visible in the narrow range scans (**Figure S 54**).

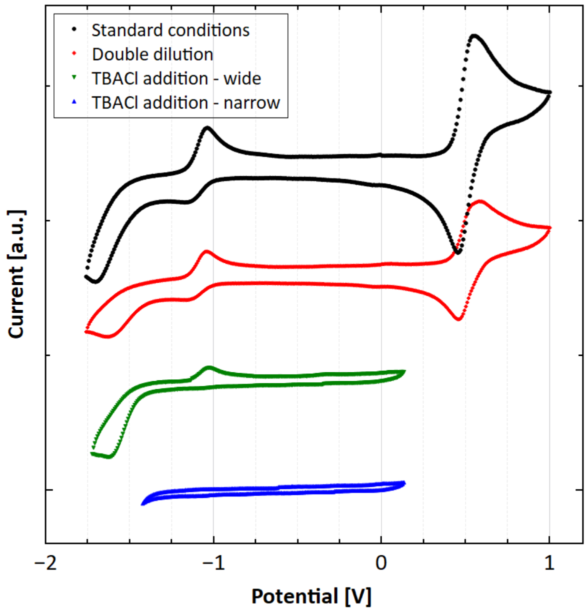

Figure S 56: Series of cyclic voltammogram experiments using compound 1 in order to identify the origin of the cathodic peak at -1.13 V. (black) CV using standard conditions (10 mL 0.1 M TBAPF$_6$), (red) addition of further 10 mL 0.1 M TBAPF$_6$; after addition of 2 mmol of TBACl scanned in a wide (green) and narrow (blue) window (all CVs are externally referenced to ferrocene).

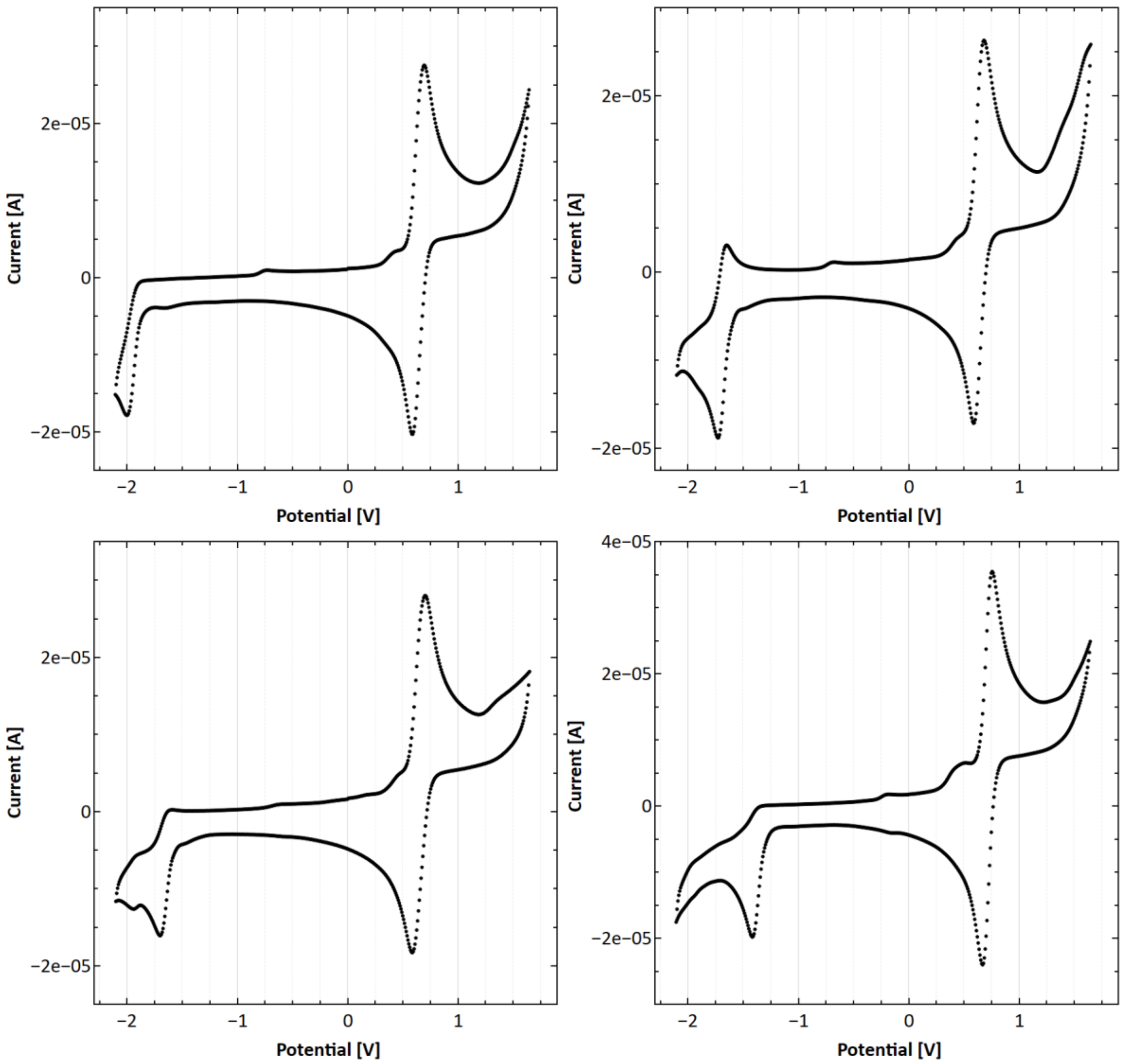

Figure S 57: Cyclic voltammograms of L1 (top left), L2 (top right), L4 (bottom left) and L7 (bottom right) in the electrochemical windows between -2.1 – 1.65 V.

Antimicrobial Studies

All complexes were screened against *S. aureus, Escherichia coli (E. coli), Klebsiella pneumoniae (K. pneumoniae), Pseudomonas aeruginosa (P. aeruginosa) and Acinetobacter baumannii (A. baumannii),* and antifungal activity against *Candida albicans (C. albicans) and Cryptococcus neoformans (C. neoformans)* (**Figure 6B**).

Although there are few distinct trends, generally, the most active complexes against *S. aureus* contain a Fc-acac ligand with neutral inductive aromatic ring systems (**2**, **3**, **8-10**). Also, the di-chloro complex **19** exhibits *ca.* 2-fold increase when compared to the mono-chloro β-diketonate complexes (**18** and **20**), which could suggest that increasing the number of chloride groups could affect the activity. A general trend of decreasing activity is observed when comparing the position and number of fluoride atoms around the ring of the ferrocenyl β-diketonate R substituent: **17** (*para*) > **15** (*meta*) > **14** (*ortho*) > **16** (di-*meta*). Interestingly, decreasing electronegativity of the halogen atoms F > Cl > Br > I cause a decrease in the activity of complexes when the halogen atom is located at the *para* position, yet an increase in activity of the complexes when the halogen atom is located at the *meta* position. The opposite observation is true for electron donating substituents (i.e., R = Me), and suggest that the inductive effects around the aromatic ring may be responsible to some degree in imparting the bacterial inhibition properties to the complexes. Complexes that were classified as active underwent HIT confirmation to determine their minimum inhibitory concentration (MIC, parentheses of **Table S17**), and complexes **2** and **9** are classified as active, with MIC values of 16 µg/mL. When addressing the fungal growth inhibition after incubation of complexes **1-24**, complexes **2** and **9** were found to be active against the *C. neoformans* strain, with inhibition concentrations of 116% and 120% respectively. Generally, these complexes exhibit significantly lower growth inhibition when compared to bis(bipyridine)ruthenium ferrocenyl β-diketonato complexes, which exhibited inhibitions of 89-100%. Interestingly, complexes **2** and **9** were the only complexes to be classed as active during the antibacterial HIT confirmation studies. Complexes **2**, **3**, **8**-**10**, **12** and **19** underwent additional hit confirmation to determine their MIC (parentheses of **Table S17**), yet they were all were inactive, with MIC values > 32 µg/mL.

To assess the complexes' potency towards normal cell types, screening was conducted against human embryonic kidney cell line, HEK293, after a 20 h incubation, and hemolysis assays conducted against human whole blood after a 1 h incubation period. The tested complexes showed varying degree of cytotoxicity towards HEK-293 cells, with complexes **9** and **12** exhibiting the highest (CC_{50} = 5.79 µg/mL) and lowest (CC_{50} = 28.37 µg/mL) cytotoxicity towards eukaryotic cells, respectively. These normal kidney results for compounds **1-24** are in contrast to the previous cytotoxicity studies performed on the ARPE-19 human retinal epithelial cells which showed no cytotoxicity at the maximum threshold of 100 µM. Hemolysis (Hm) results, on the other hand, were extremely positive and highlight that of the tested complexes, all exhibited no potency towards human blood at the maximum tested concentration of 32 µg/mL, which is important for the distribution of these complexes in the bloodstream.

Table S 19: Growth inhibition for compounds 1-24 against bacterial strains S. aureus (Sa), E. coli (Ec), K. pneumonia (Kp), P. aeruginosa (Pa) and A. baumannii (Ab) and antifungal strains C. albicans (Ca) and C. neoformans (Cn). The MIC (µg/mL) are shown in parentheses for selected complexes. CC_{50} against HEK-293 (human embryonic kidney, Hk) cell line and HC_{50} against human whole blood (Hm) are also presented.

	**Inhibition (%) against bacterial strains**					**Inhibition (%) against bacterial strains**		**CC_{50} µg/mL**	**HC_{10} µg/mL**
**Compound**	***Sa***	***Ec***	***Kp***	***Pa***	***Ab***	***Ca***	***Cn***	***Hk***	***Hm***
**1**	34.22	-29.09	10.46	-22.97	30.61	7.16	-18.91	--	--
	93.65	-25.98	7.11	-33.19	18.91	25.32	116.19	21.38	>32
**2**	(16)	(>32)	(>32)	(>32)	(>32)	(> 32)	(> 32)		
	83.43	-49.1	0.52	-33.93	11.05	20.22	59.6	11.94	>32
**3**	(>32)	(>32)	(>32)	(>32)	(>32)	(> 32)	(> 32)		
**4**	29.52	-24.87	-1.68	-13.98	11.61	46.13	-18.6	--	--
**5**	75.12	-20.02	0.46	-20.43	17.98	30.27	-19.96	--	--
**6**	23.53	-35.27	6.05	-16.69	13.58	11.06	-17.24	--	--
**7**	27.64	-15.35	8.87	-14.99	30.09	18.53	-21.78	--	--
	88.03	-22.03	2.70	-49.1	10.04	7.77	-6.5	14.79	>32
**8**	(>32)	(>32)	(>32)	(>32)	(>32)	(>32)	(>32)		
	85.24	-22.37	4.43	-33.05	22.24	12.81	119.73	5.79	>32
**9**	(16)	(>32)	(>32)	(>32)	(>32)	(>32)	(>32)		
	94.64	-13.00	5.99	-24.77	7.47	4.27	-11.64	20.45	>32
**10**	(>32)	(>32)	(>32)	(>32)	(>32)	(> 32)	(> 32)		
**11**	29.91	-23.77	-0.71	-40.74	9.38	8.57	-14.67	--	--
	90.91	-10.07	7.62	-22.57	28.15	7.7	-13.91	28.37	>32
**12**	(>32)	(>32)	(>32)	(>32)	(>32)	(>32)	(>32)		
**13**	69.07	-22.29	0.38	-25.37	22.4	10	-13.61	--	--
**14**	12.06	-20.48	4.71	-19.35	2.95	1.86	-6.95	--	--
**15**	31.77	-40.32	-1.28	-20.53	18.45	6.96	-21.63	--	--
**16**	11.5	-45.46	-1.28	-32.88	9.73	-0.84	-16.03	--	--
**17**	58.92	-37.82	2.56	-22.47	25.81	17.93	-19.81	--	--
**18**	49.6	-42.9	0.03	-29.13	15.11	2.67	-15.88	--	--
	86.29	-31.94	2.17	-32.12	8.22	8.98	-10.28	6.27	>32
**19**	(>32)	(>32)	(>32)	(>32)	(>32)	(>32)	(>32)		
**20**	51.92	-44.24	6.09	-23.9	12.63	9.99	-15.73	--	--
**21**	57.51	-34.73	0.92	-22.19	15.77	13.02	-16.49	--	--
**22**	43.52	-33.89	6.65	-40.78	21.16	2.74	-18.15	--	--
**23**	57.13	-27.9	6.54	-22.07	16.76	8.71	-18.15	--	--
**24**	24.07	-17.95	7.29	-38.57	18.12	3.39	-17.85	--	--

References

1. Cosier, J.; Glazer, A. M., A nitrogen-gas-stream cryostat for general X-ray diffraction studies. *Journal of Applied Crystallography* **1986,** *19* (2), 105-107.
2. CrysAlisPRO *CrysAlisPRO, Oxford Diffraction*, Agilent Technologies UK Ltd: Yarnton, England.
3. Sheldrick, G. M., SHELXT - Integrated space-group and crystal-structure determination. *Acta Cryst.* **2015,** *A71*, 3-8.
4. Sheldrick, G. M., Crystal structure refinement with SHELXL. *Acta Cryst. Section C* **2015,** *71* (1), 3-8.
5. O. V. Dolomanov; L. J. Bourhis; R. J. Gildea; Howard, J. A. K.; Puschmann, H., OLEX2: a complete structure solution, refinement and analysis program. *J. Appl. Cryst.* **2009,** *42*, 339-341.
6. Spek, A. L., PLATON SQUEEZE: a tool for the calculation of the disordered solvent contribution to the calculated structure factors. *Acta Crystallogr C Struct Chem* **2015,** *71* (Pt 1), 9-18.
7. Connelly, N. G.; Geiger, W. E., Chemical Redox Agents for Organometallic Chemistry. *Chemical Reviews* **1996,** *96* (2), 877-910.
8. Allison, M.; Caramés-Méndez, P.; Pask, C. M.; Phillips, R. M.; Lord, R. M.; McGowan, P. C., Bis(bipyridine)ruthenium(II) Ferrocenyl β-Diketonate Complexes: Exhibiting Nanomolar Potency against Human Cancer Cell Lines. *Chemistry – A European Journal* **2021,** *27* (11), 3737-3744.
9. Allison, M.; Wilson, D.; Pask, C. M.; McGowan, P. C.; Lord, R. M., β-Diketonate versus β-Ketoiminate: The Importance of a Ferrocenyl Moiety in Improving the Anticancer Potency. *ChemBioChem* **2020,** *21* (14), 1988-1996.
10. Manikandan, M.; Gadre, S.; Chhatar, S.; Chakraborty, G.; Ahmed, N.; Patra, C.; Patra, M., Potent Ruthenium–Ferrocene Bimetallic Antitumor Antiangiogenic Agent That Circumvents Platinum Resistance: From Synthesis and Mechanistic Studies to In Vivo Evaluation in Zebrafish. *Journal of Medicinal Chemistry* **2022,** *65* (24), 16353-16371.
